# Direct Construction of *C*‐Alkyl Glycosides from Non‐Activated Olefins via Nickel‐Catalyzed C(sp^3^)─C(sp^3^) Coupling Reaction

**DOI:** 10.1002/advs.202307226

**Published:** 2024-01-18

**Authors:** Changyue Yu, Yinghuan Xu, Mingjie Zeng, Jingjing Wang, Wenhao Dai, Jiang Wang, Hong Liu

**Affiliations:** ^1^ State Key Laboratory of Drug Research Shanghai Institute of Materia Medica Chinese Academy of Sciences Shanghai 201203 China; ^2^ School of Pharmacy University of Chinese Academy of Sciences Beijing 100049 China; ^3^ School of Pharmacy China Pharmaceutical University Nanjing 211198 China; ^4^ Lingang Laboratory Shanghai 200031 China

**Keywords:** *C*‐alkyl glycosides, C(sp3)─C(sp3) coupling, non‐activated olefins, nickel catalysis

## Abstract

Among *C*‐glycosides, *C*‐alkyl glycosides are significant building blocks for natural products and glycopeptides. However, research on efficient construction methods for *C*‐alkyl glycosides remains relatively limited. Compared with Michael acceptors, non‐activated olefins are more challenging substrates and have rarely been employed in the construction of *C*‐glycosides. Here, a highly efficient and convenient approach for the synthesis of *C*‐alkyl glycosides through a nickel‐catalyzed C(sp^3^)‐C(sp^3^) coupling reaction is presented. A distinctive feature of this method is its utilization of non‐activated olefins as the anomeric radical acceptors for hydroalkylation, allowing for the direct formation of *C*‐glycoside bonds in a single step. Furthermore, this method demonstrates excellent compatibility with a broad scope of highly reactive functional groups. Mechanistic investigations suggest that the reaction proceeds via a free radical pathway, leading predominantly to the formation of products with α‐configuration. Overall, this innovative methodology offers a versatile and practical approach for the synthesis of *C*‐alkyl glycosides, offering new avenues for the production of intricate glycosides with potential applications in drug discovery and chemical biology.

## Graphical abstract

The nickel‐catalyzed C(sp^3^)‐C(sp^3^) coupling hydroalkylation method is reported for the direct synthesis of *C*‐alkyl glycosides. This innovative approach involves the reaction between glycosyl bromides and readily available non‐activated olefins, including various natural products and peptides. These results demonstrate the versatility of the synthetic method, indicating its potential as a valuable tool for the efficient preparation of diverse *C*‐glycosides.

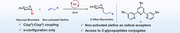

## 1 Introduction

**Figure 1:**
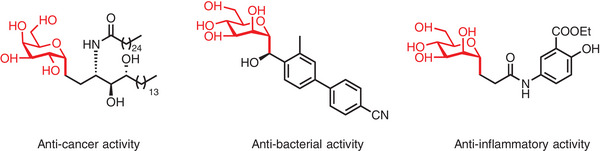
Representative structures of *C*‐alkyl glycosides.

*C*‐glycosides, abundant in both natural products and synthetic bioactive molecules, hold great promise as candidates for drug development. In particular, the structure of *C*‐alkly glycosides is present in a variety of important compounds with potential biological activities, such as anticancer, anti‐bacterial, or anti‐inflammatory (**Figure** **1**).^[^ ^1^, ^2^ ^]^ Considerable progress has been witnessed in the construction of *C*‐glycosides in recent decades.^[^ ^3^ ^]^ Novel and efficient synthetic techniques have displayed substantial potential in the synthesis of *C*‐glycosides, especially *C*‐aryl and *C*‐alkenyl glycosides, involving methods such as C─H activation and cross‐coupling.^[^ ^4^, ^5^ ^]^ However, despite the importance of *C*‐alkyl glycosides as building blocks for natural products and drugs, research on efficient construction methods for them remains limited.

**Scheme 1:**
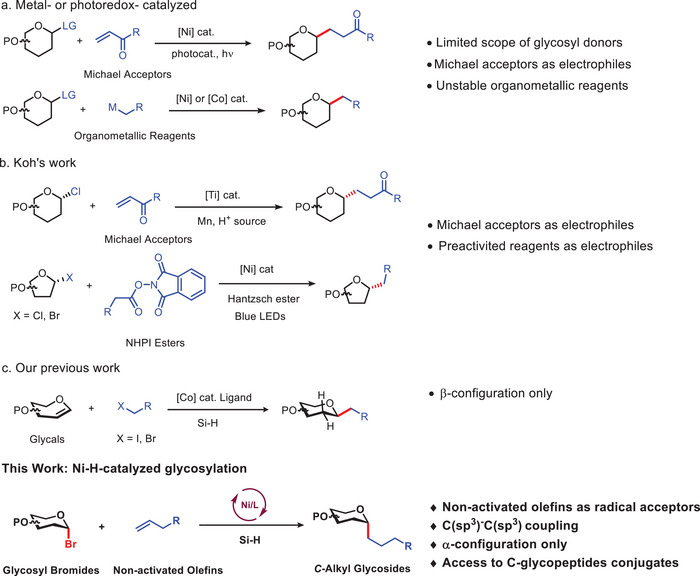
Metal‐catalyzed coupling reaction for the construction of *C*‐alkyl glycosides.

Current approaches for constructing *C*‐alkyl glycosides encompass non‐catalytic and catalytic methods. Non‐catalytic synthetic methods usually depend on a radical initiator in combination with stoichiometric amounts of a hydrogen atom donor.^[^ ^6^ ^]^ Nonetheless, the utilization of stoichiometric reagents and the introduction of toxic species, such as *n*‐Bu_3_SnH, significantly limit the utility of this approach. In contrast, catalytic approaches involving transition metal or photoredox catalysis have been developed for the synthesis of *C*‐alkyl glycosides.^[^ ^7^ ^]^ However, the availability of the glycosyl donor is typically limiting in the reaction, and the other reactants often involve activated olefins, such as Michael acceptors or highly reactive and unstable organometallic reagents, which are sensitive to air and moisture, with limited functional group compatibility (**Scheme** **1**a). Recently, Koh et al. reported a series of transition metal‐catalyzed approaches for synthesizing *C*‐alkyl glycosides (Scheme 1b).^[^ ^8^ ^]^ They employed a stereoselective Ti(III)‐catalyzed regime that effectively merged a diverse range of readily available glycosyl chlorides with activated olefins. Additionally, they developed a method for coupling with glycosyl halides and redox‐active electrophiles such as *N*‐(acyloxy)phthalimides (NHPI esters) or pyridinium salts. Although they utilized simple commercially available glycosyl halides as glycosyl donors, the other coupling reactants still included activated olefins, amines, or carboxylic acids. Therefore, it remains crucial to develop a reaction using conventional and straightforward reagents for coupling to obtain *C*‐alkyl glycosides. Compared with activated olefins, such as Micheal acceptors, aliphatic olefins, which are more abundant in nature and industry, are more challenging substrates and have rarely been employed in the construction of *C*‐glycosides, due to the low reactivity of both reactants and poor control of selectivity. Therefore, it is highly desirable to develop an alternative strategy to overcome the intrinsic limitations of aliphatic olefins substrates and enhance reactivity.^[^ ^9^ ^]^ Our group recently reported a ligand‐controlled cobalt(II)‐catalyzed hydroalkylation reaction for the β‐selective synthesis of 2‐deoxy‐*C*‐glycosides from glycals (Scheme 1c).^[^ ^10^ ^]^ Expanding upon our previous work, we initiated an investigation into a nickel‐catalyzed C(sp^3^)─C(sp^3^) coupling reaction to enable the direct synthesis of *C*‐alkyl glycosides. This innovative method utilizes readily available glycosyl bromides and non‐activated olefins as substrates, eliminating the necessity for directing or activating groups. The hydroalkylation reaction proceeds directly, demonstrating its versatility across a broad range of glycosyl donors and olefins. Remarkably, this method exhibits exceptional functional‐group compatibility, enabling the incorporation of natural products or peptides as substrates. One of the notable advantages of this method is its ability to offer new strategies for late‐stage *C*‐glycosylation of natural products, as well as the synthesis of *C*‐glycoamino acids or *C*‐glycopeptides.

## 2 Results and Discussion

### 2.1 Optimization of Reaction Conditions

To investigate the optimal reaction conditions for the direct synthesis of *C*‐alkyl glycosides via hydroalkylation, we selected mannosyl bromide **1a** and octene **2a** as the model substrate. In accordance with our previous research, a combination of NiBr_2_(diglyme) and **L1**, along with diethoxymethylsilane (DEMS) and CsF in 1,4‐dioxane, was crucial for achieving a 76% yield of *C*‐alkyl glycoside **3aa** with excellent diastereoselectivity (α‐configuration only) after the systematic screening of all reaction parameters (**Table** **1**, entry 1). The influence of ligands, Ni catalysts, hydride sources, bases, and solvents are described in Table S3 (Supporting Information). A key summary is shown in Table 1. Some Ni catalysts were also tested, and when NiBr_2_·DME or NiI_2_ was used instead of NiBr_2_(diglyme), the resulting yields decreased to 68% or 39%, respectively (Table 1, entries 2–3). Slight variations in the silane from DEMS to (EtO)_3_SiH or polymethylhydrosiloxane (PHMS) failed to improve the efficiency (Table 1, entries 4–5). Subsequently, we conducted an investigation into suitable bases and observed that utilizing K_3_PO_4_ or Cs_2_CO_3_ instead of CsF resulted in a decreased yield, although the reaction still proceeded (Table 1, entries 6–7). An optimization of the solvent demonstrated that 1,4‐dioxane was the optimal choice (see Supporting Information for details). Further examination of the ligand in the model reaction revealed that the selection of the tridentate *N*‐ligand was critical to the success of this reaction. Various ligands, such as bipyridine (**L2**), bioxazoline (**L3**), and pyridine‐oxazoline (**L4**), resulted in almost no corresponding product (Table 1, entries 8–10). However, the use of 2,2′:6′,2′“‐terpyridine as a tridentate ligand (**L5**) significantly improved the coupling efficiency (Table 1, entry 11), with further yield improvements observed when utilizing 4,4′,4′”‐*tri*‐*tert*‐butyl‐2,2′:6′,2′'‐terpyridine (**L1**). Finally, to illustrate the significance of the catalyst and ligand in the reaction, we conducted a reaction without the catalyst or ligand, which failed to produce the target compound **3aa** (see Supporting Information for details).

**Table 1:** Optimization of reaction conditions.

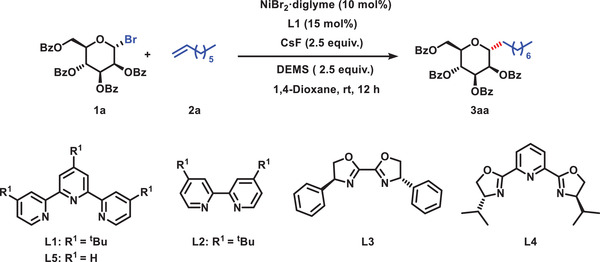			
Entry	Variants	Yield (%)^a)^	α: β
1	None	76%	α only
2	NiBr_2_·DME	68%	α only
3	NiI_2_	39%	α only
4	(EtO)_3_SiH	64%	α only
5	PHMS	58%	α only
6	K_3_PO_4_	70%	α only
7	Cs_2_CO_3_	26%	α only
8	L2	Trace	–
9	L3	Trace	–
10	L4	Trace	–
11	L5	42%	α only

^a)^ Isolated yield. Reactions were carried out under an argon atmosphere. Conditions: **1a** (2.0 equiv.), **2a** (1.0 equiv.), Ni catalyst (10 mol%), ligand (15 mol%), silane (2.5 equiv.), base (2.5 equiv.), solvent (1 mL), room temperature, 12 h. PMHS, polymethylhydrosiloxane. Diglyme, Bis(2‐methoxyethyl) ether. DME, 1,2‐Dimethoxyethane.

### 2.2 Substrate Scope

With the optimized reaction conditions in hand, our attention turned towards exploring the compatibility of non‐activated olefin coupling partners. As shown in **Table** **2**, the catalytic system was found to be compatible with various non‐activated olefin substrates, demonstrating its broad applicability. During our investigations, we assessed several linear aliphatic olefins, including hexene and heptene. Remarkably, these substrates readily underwent hydroalkylation coupling with glycosyl donors, consistently yielding the expected products in good to moderate yields (**3ab**‐**3ac**, 73−75%) with excellent diastereoselectivity (α‐configuration only). However, it is noteworthy that branched olefins showed lower yields, highlighting the influence of steric hindrance on the reaction outcome (**3ad**‐**3ae**, 52−56%). Furthermore, phenyl‐substituted olefins exhibited good yields, emphasizing the ability to accommodate a wide range of substrates (**3af**‐**3ah**, 64−70%). The mild reaction conditions allowed for compatibility with numerous valuable synthetic functional groups, including chlorine (**3al**), bromine (**3ai**‐**3ak**), cyano (**3am**), amide (**3an**), ester (**3ao**, **3aq**), carbonyl (**3ap**), and orthoester (**3ar**). Aryl ether olefin derivatives, whether containing electron‐donating (**3an**) or electron‐withdrawing (**3am**) groups, still underwent glycosylation reactions. Interestingly, acetamido groups introduced onto phenyl moieties proceeded smoothly, indicating tolerance toward the N‐H bond of the amide. The hydroalkylation reaction also accepted heterocyclic structures, such as furan, and demonstrated orthocompatibility with boronate derivatives (**3as**), opening avenues for further transition metal‐catalyzed transformations. Remarkably, the reaction exhibited resistance towards epoxide with highly strained configuration, maintaining the original configuration of epoxide and preventing the ring‐opening reactions (**3at**). Silicon‐containing substituent can also proceed smoothly in this reaction (**3au**). Even unprotected hydroxyl substituted substrate also yielded the desired product (**3av**), indicating the minimal impact of polar groups on the reaction. This remarkable insight indicates the potential elimination of protection and deprotection steps in the modification of natural products and drugs. Expanding the scope of application, we applied it to access *C*‐glycosides modified amino acid (**3aw**), providing an innovative route for synthesizing *C*‐alkyl glycoamino acids. Furthermore, cyclic alkene such as cyclohexene was also accommodated under identical conditions to give hydroalkylation product (**3ax**) in an acceptable yield with α‐configuration only. However, for terminal disubstituted alkene such as 3‐methylenepentane (**2y**), it is difficult to obtain the corresponding product (**3ay**) in this reaction system. We speculate that steric hindrance might be the reason for the lack of success in this particular reaction.

**Table 2:** Scope of non‐activated olefins and glycosyl donors.^a)^

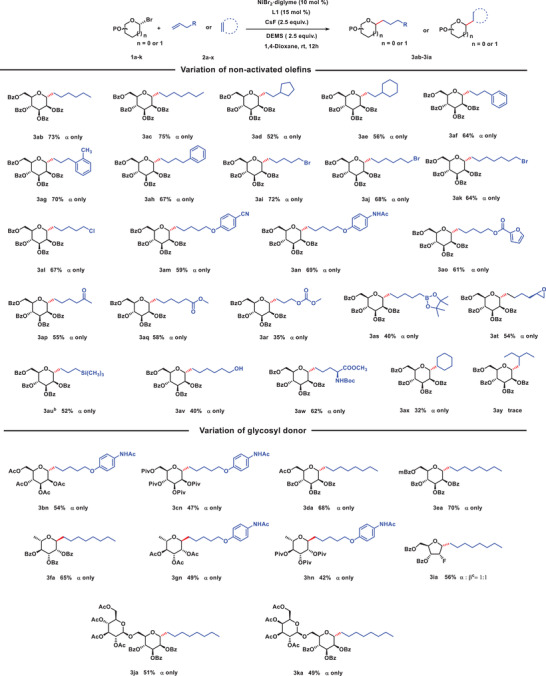

^a)^ Isolated yield, ^b^K_3_PO_4_ as the base, ^c^α: β was determined by ^1^H NMR.

In carbohydrate chemistry synthesis, the choice of carbohydrate groups and protecting groups plays a critical role in the reaction. Under our established catalytic conditions, a wide range of glycosyl bromide donors, each featuring distinct protective groups, effectively engaged in the catalytic system, resulting in medium to high yields of the corresponding *C*‐glycosyl arenes **3bn**–**3ka** as shown in Table 2. Interestingly, the incorporation of acetyl (**2b**, **2g**) or pivaloyl (**2c**, **2h**) moieties as protective groups led to comparable glycosylation reactions, albeit with slightly reduced yields (42−54%). The decrease in yield is likely attributed to the electronic or steric hindrance introduced by the protective group. We further explored the versatility of this method by introducing various substituents, such as acetyl (**2d**) or 4‐methoxybenzoyl (**2e**), at the 6‐position of d‐mannose. Remarkably, the glycosylation reaction proceeded smoothly, underscoring the adaptability of this method to accommodate various modifications at this site. These modifications could include the conjugation of natural products or drugs, expanding the range of potential applications. Additionally, we investigated the utilization of various types of carbohydrate groups as glycosyl donor. Notably, when l‐bromorhamnose was employed as the glycosyl donor, the desired products (**3fa**, **3gn**, and **3hn**) were obtained in moderate yields with exclusive α‐configuration. Moreover, the utilization of acetyl and pivaloyl‐protected l‐rhamnose still delivered C‐alkyl glycosides derivatives with acceptable yields. Regrettably, when bromofuranose (**2i**) was employed as the glycosyl donor, although the intended *C*‐alkyl glycosides product was obtained, the α and β configuration products were nearly indistinguishable with a mixture of the two configurations in a ratio of ≈1:1. In addition, this reaction is not only applicable to monosaccharides, but also enables the synthesis of the target products (**3ja** and **3ka**) in moderate yields (51% and 49%, respectively) with α‐configuration only for disaccharides.

### 2.3 Synthetic Application

**Scheme 2:**
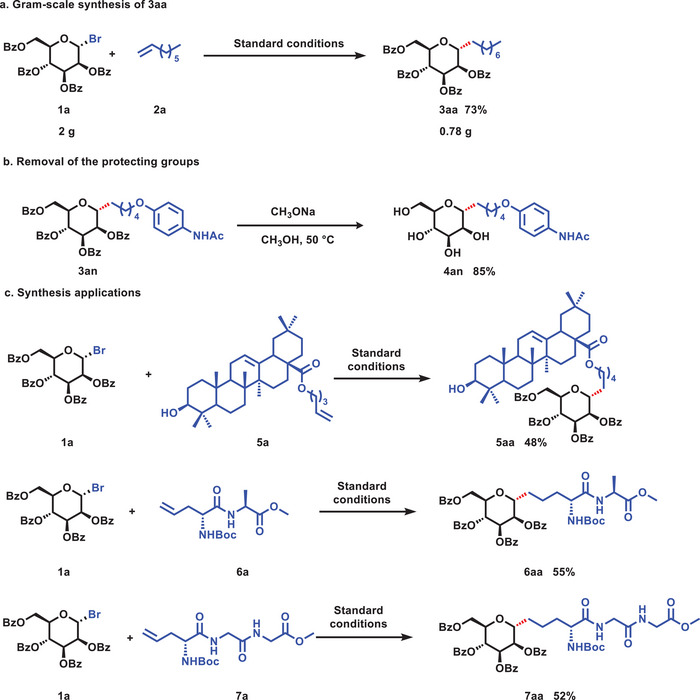
Gram‐scale synthesis, removal of the protecting groups, and synthetic applications.

To expand the potential applicability of this synthetic method, a series of experiments were conducted. In the gram‐scale preparation, compound **1a** as the starting material was reacted with non‐activated olefin **2a** under standard conditions, resulting in the formation of the desired *C*‐glycoside product **3aa** in a yield of 73% with excellent diastereoselectivity (α‐configuration only). This gram‐scale experiment illustrated the scalability of this reaction, enhancing the practicality of this method. Subsequently, we performed a deprotection experiment on the target product **3an**. Specifically, the *C*‐glycoside product **3an** was reacted with sodium methoxide in a methanol solution at 50°C for 4 hours, leading to the formation of the desired product **4an** with an impressive isolated yield of 85%. To underscore the exceptional functional group compatibility of the reductive olefin hydroalkylation reaction, its potential as a versatile tool for the modification of complex, biologically relevant molecules and peptides was leveraged. These findings demonstrate the broad scope of this synthetic method and its potential applications in diverse fields, including medicinal chemistry and drug discovery. As an example, the *C*‐glycoside experiment was performed on a conjugate of the natural product oleanolic acid. Specifically, the treatment of a derivative of oleanolic acid (**5a**), resulted in the chemo‐selective formation of **5aa** without affecting the internal olefin or hydroxyl groups. In addition, *C*‐alkyl glycosides and glycoproteins are widely found in nature and are renowned for their diverse biological activities. For instance, peptidyl nucleoside antibiotics, such as Nikkomycin Z^[^ ^11^ ^]^ and Amipurimycin,^[^ ^12^ ^]^ exhibit exceptional antimycotic properties. Moreover, glycosylation serves as an effective synthetic strategy that can substantially enhance the bioavailability of endogenous peptides, such as, *C*‐linked glycosyl decapeptide,^[^ ^13^ ^]^ [(αGal)Ala^7^]dermorphin,^[^ ^14^ ^]^ and galactosylated‐NAPamide.^[^ ^15^ ^]^ We have also investigated the synthesis of glycopeptide derivatives using the reductive olefin hydroalkylation reaction. Remarkably, we were able to obtain the corresponding glycopeptide derivatives of both dipeptide (**6aa**) and tripeptide (**7aa**) with yields of 55% and 52%, respectively. This successful synthesis of glycopeptide derivatives represents a significant advancement in peptide synthesis, offering a more convenient route for preparing these crucial compounds. These results highlight the substantial potential of our synthetic method for creating complex molecules, particularly those with biological relevance. Through enabling selective modifications of multiple functional groups in a convergent manner, our approach provides a valuable strategy for the synthesis of a wide range of bioactive compounds. **Scheme** **2**

### 2.4 Mechanistic Studies

To gain a deeper insight in the mechanism underlying the reductive olefin hydroalkylation reaction, we conducted a radical trapping experiment using 2,2,6,6‐tetramethylpiperidinooxy (TEMPO). Remarkably, this experiment resulted in a complete inhibition of the reaction, with the observation of the addition product of olefin and TEMPO through LC‐MS, identified as **3an’**. Furthermore, deuterium‐labeling experiments were carried out using Ph_2_SiD_2_ to investigate the source of hydrogen in this hydroalkylation reaction. Significantly, under standard conditions with Ph_2_SiD_2_, we observed deuterium incorporation in chain alkanes, indicating that silane serves as the hydrogen source in this reaction. These results provide valuable insights into the mechanism of the reductive olefin hydroalkylation reaction, illuminating the roles of radicals and hydrogen sources in this process.

**Scheme 3:**
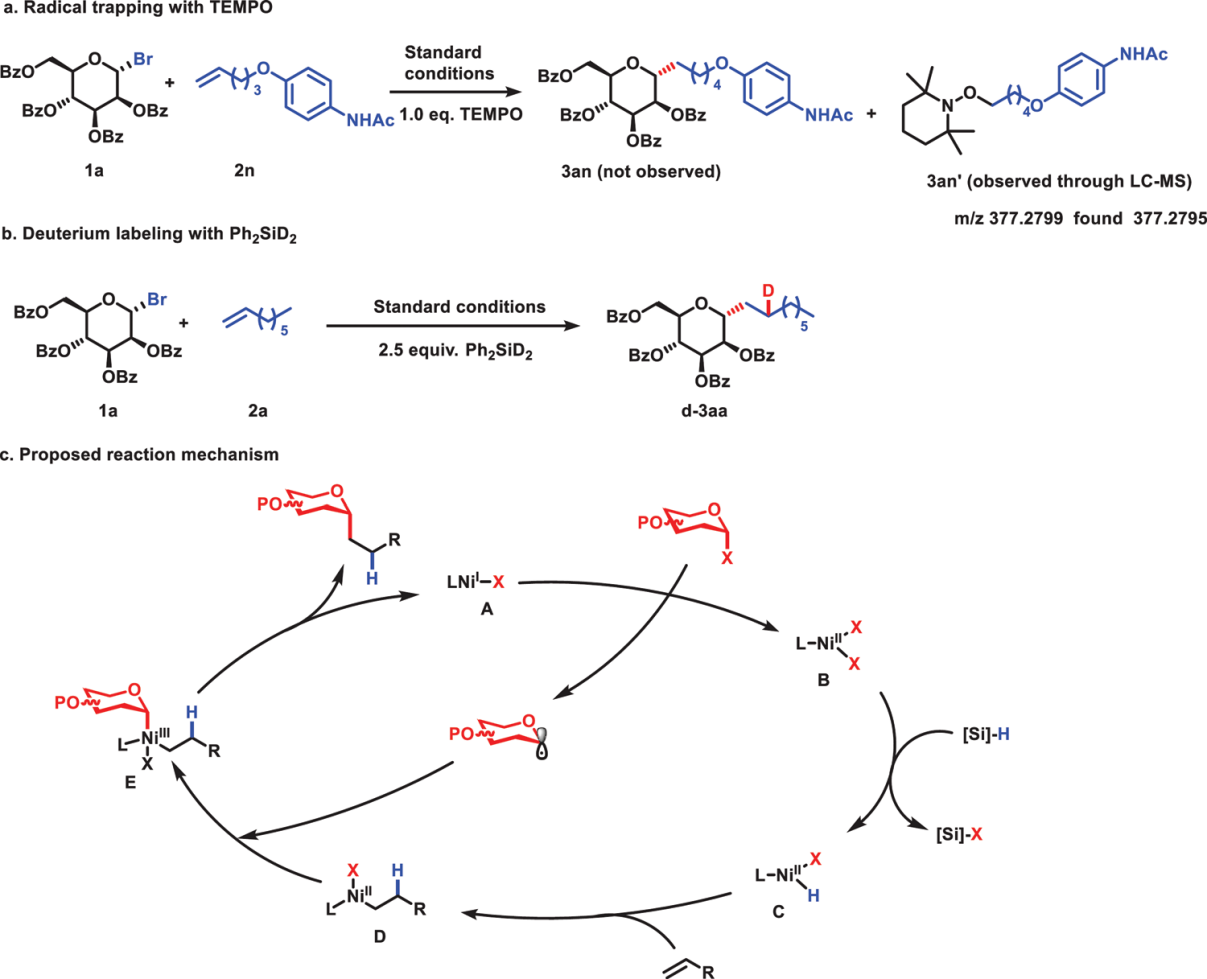
Mechanistic studies and proposed reaction mechanism.

On the basis of previous studies and the above results,^[^ ^16^ ^]^ we have proposed a plausible mechanism for the reductive olefin hydroalkylation reaction with glycosyl participation, involving a Ni(I)/Ni(II)/Ni(III) catalytic cycle, as depicted in **Scheme** **3**c. Initially, Ni(I) species **A** is proposed to activate a glycosyl halide to give Ni(II) species **B** and a glycosyl radical. The Ni(II) species **B** undergoes transmetalation with the silane, generating the Ni‐H species **C**. Subsequently, the species **C** is inserted into the C‐C double bond, leading to an addition reaction and the formation of an alkyl nickel species **D**, which can quickly capture the glycosyl radical and undergo a radical addition reaction, resulting in the formation of the Ni(III) species **E**. Finally, reductive elimination from **E** produces the desired *C*‐alkyl glycoside product and Ni(I) species **A**, which regenerates the catalyst Ni(II) complex **B** and the glycosyl radical by reacting with a glycosyl halide.

## 3 Conclusion

In summary, we present a highly efficient and convenient method for synthesizing *C*‐alkyl glycosides using a nickel‐catalyzed C(sp^3^)‐C(sp^3^) coupling reaction with non‐activated olefins. The strategy enables the direct formation of C‐C bonds, facilitating the synthesis of *C*‐glycosides in a highly efficient manner through the hydroalkylation of non‐activated olefins. To the best of our knowledge, this is the first report of synthesizing *C*‐alkyl glycosides using non‐activated olefins as radical acceptors, overcoming the challenge of low reactivity and poor control of selectivity. Additionally, this reaction exhibits remarkable compatibility with a wide range of highly reactive functional groups, including esters, epoxides, borate esters, and hydroxyl groups. This broad compatibility and excellent stereoselectivity enhances the versatility and applicability of this method. We also elucidate the free radical pathway underlying the formation of α‐configured products in a versatile reaction for *C*‐glycosides synthesis. These results demonstrate the flexibility of this synthetic method and suggest its potential as a valuable tool for the preparation of diverse *C*‐glycosides. Further optimization of this approach may lead to the development of new strategies for selectively synthesizing specific glycoside configurations.

## Conflict of Interest

The authors declare no conflict of interest

## Supporting information

Supporting Information

## Data Availability

The data that support the findings of this study are available from the corresponding author upon reasonable request.
