# Supplementary material for: Direct Construction of C‐Alkyl Glycosides from Non‐Activated Olefins via Nickel‐Catalyzed C(sp3)─C(sp3) Coupling Reaction
Source: Adv Sci (Weinh). 2024 Jan 18;11(12):2307226. doi: 10.1002/advs.202307226 (PMC10966551; doi:10.1002/advs.202307226)

ADVANCED SCIENCE
Open Access

Supporting Information

for *Adv. Sci.*, DOI 10.1002/advs.202307226

Direct Construction of *C*-Alkyl Glycosides from Non-Activated Olefins via Nickel-Catalyzed $C(sp^3)$—$C(sp^3)$ Coupling Reaction

Changyue Yu, Yinghuan Xu, Mingjie Zeng, Jingjing Wang, Wenhao Dai, Jiang Wang and Hong Liu**

Supporting Information

Direct Construction of *C*-Alkyl Glycosides from Non-activated Olefins via Nickel-Catalyzed $C(sp^3)$-$C(sp^3)$ Coupling Reaction

Changyue Yu,[+] Yinghuan Xu,[+] Mingjie Zeng, Jingjing Wang, Wenhao Dai, Jiang Wang,[*] and Hong Liu[*]

Table of Contents

I. General Information.......... S1

II. General Procedures for the Preparation of Substrates.......... S2

III. Supplementary Table of Optimization Studies.......... S6

IV. General Procedures for the Preparation of C-alkyl Glycosides.......... S8

V. Scale-up Reaction and Further Transformation.......... S35

VI. Mechanistic Investigation.......... S37

VII. References.......... S39

VII. NMR Spectra.......... S40

I. General Information

General procedures of the synthesis route were described as below. Reagents (chemicals) were purchased from J&K®, bidepharm, leyan, Energy, and Adamas, and used without further purification. Analytical thin-layer chromatography (TLC) was performed on HSGF 254 (150–200 μm thickness, leyan Company, China). All products were characterized by their NMR, LRMS and HRMS spectra. Nuclear magnetic resonance (NMR) spectra were performed on a Brucker AMX-400, AMX-500, and AMX-600 NMR (TMS as IS). Chemical shifts were reported in parts per million (ppm, δ) downfield from tetramethylsilane. Proton coupling patterns were described as singlet (s), doublet (d), doublet of doublets (dd), triplet (t), triplet of doublets (td), quartet (q), multiplet (m), etc. Low-resolution mass spectra (LRMS) data was obtained on a Thermo Fisher Finnigan LEQ with electrospray ionization (ESI). High resolution mass spectra (HRMS) was measured on a Micromass Ultra Q-TOF spectrometer. Column chromatography was performed on silica gel (300-400 mesh) using *n*-hexane/ethyl acetate. All of the heating reactions are carried out in oil bath.

II. General Procedures for the Preparation of Substrates

Table S1. Non-activated Olefins 2a–2y, 5a, 6a, 7a

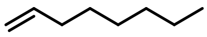
2a

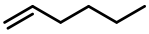
2b

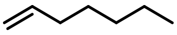
2c

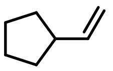
2d

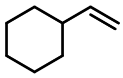
2e

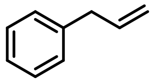

2f

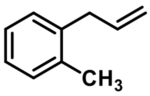

2g

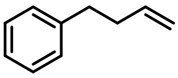
2h

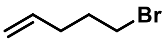

2i

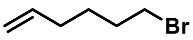

2j

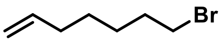

2k

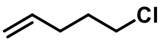

2l

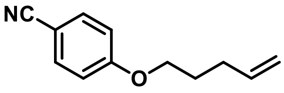

2m

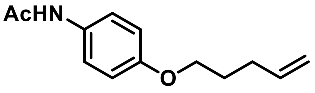

2n

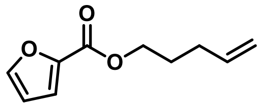

2o

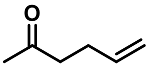

2p

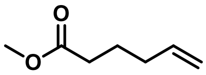

2q

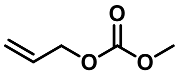

2r

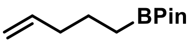

2s

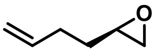

2t

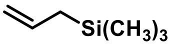

2u

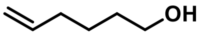

2v

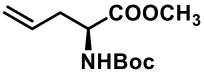

2w

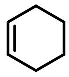
2x

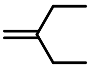
2y

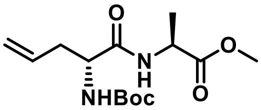

6a

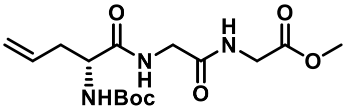

7a

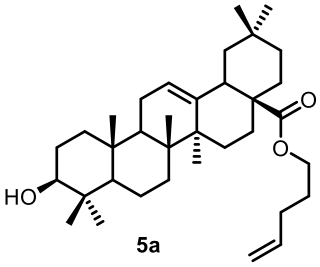

5a

Alkyl halides **2a-2y** were commercially available.

General Procedures for Preparation of 5a

Scheme S1. Procedures for the preparation of 5a

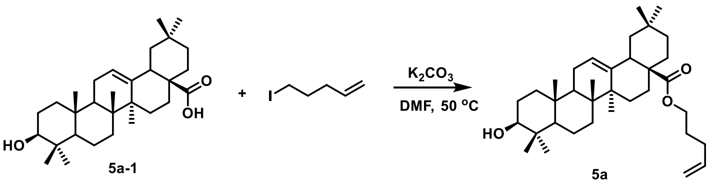

A mixture of **5a-1** (3.0 g, 6.57 mmol), 5-iodopent-1-ene (1.93 g, 9.85 mmol), and K_2CO_3 (2.72 g, 19.71 mmol) in DMF (20 mL) was stirred at 50 °C overnight. After the reaction is completed, water was added and the mixture was extracted with dichloromethane. The combined organic layer was washed with saturated sodium thiosulfate, water, and saturated sodium chloride, dried over anhydrous Na_2SO_4, and concentrated in vacuo. The resulting residue was purified by silica gel flash chromatography to give the desired product **5a** (3.2 g, 93%).

5a: ^{1}H NMR (600 MHz, Chloroform-*d*) δ 5.83 (ddt, *J* = 16.9, 10.2, 6.6 Hz, 1H), 5.31 (t, *J* = 3.7 Hz, 1H), 5.06 (dq, *J* = 17.2, 1.7 Hz, 1H), 5.01 (dq, *J* = 10.2, 1.5 Hz, 1H), 4.05 (t, *J* = 6.5 Hz, 2H), 3.23 (dd, *J* = 11.4, 4.4 Hz, 1H), 2.90 (dd, *J* = 13.8, 4.7 Hz, 1H), 2.16 (q, *J* = 7.1 Hz, 2H), 1.98 (td, *J* = 14.9, 4.0 Hz, 1H), 1.90 (dt, *J* = 11.2, 3.8 Hz, 2H), 1.77 – 1.26 (m, 17H), 1.24 – 1.17 (m, 2H), 1.16 (s, 3H), 1.11 – 1.05 (m, 1H), 1.01 (s, 3H), 0.95 (s, 3H), 0.93 (s, 3H), 0.92 (s, 3H), 0.80 (s, 3H), 0.76 (s, 3H), 0.75 – 0.73 (m, 1H). **LRMS**(ESI): 525.8 $[M+H]^+$

General Procedures for Preparation of 6a and 7a

Scheme S2. Procedures for the preparation of 6a

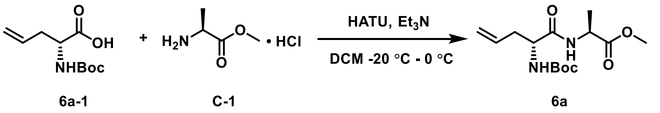

A mixture of **6a-1** (1.0 g, 4.65 mmol) and HATU (2.65 g, 6.97 mmol) was stirred at -20 °C for 1 h, followed by Et_3N (1.41 g, 13.94 mmol) and **C-1** (0.778 mg, 5.57 mmol). The mixture was allowed to stir at 0 °C for 12 h. After the reaction is completed, water was added and the mixture was extracted with dichloromethane. The combined organic layer was washed with saturated sodium thiosulfate, water, and saturated sodium chloride, dried over anhydrous Na_2SO_4, and concentrated in vacuo. The resulting residue was purified by silica gel flash chromatography to give the desired product **6a**

(1.20g, 85%).

6a:**^{1}H NMR** (600 MHz, Chloroform-*d*) δ 6.68 (s, 1H), 5.88 – 5.67 (m, 1H), 5.19 (d, *J* = 5.0 Hz, 1H), 5.16 (s, 1H), 5.01 (s, 1H), 4.60 (p, *J* = 7.2 Hz, 1H), 4.21 (s, 1H), 3.77 (s, 3H), 2.60 – 2.44 (m, 2H), 1.47 (s, 9H), 1.42 (d, *J* = 7.2 Hz, 3H).

LRMS(ESI): 301.3 $[M+H]^+$

Scheme S3. Procedures for the preparation of 7a

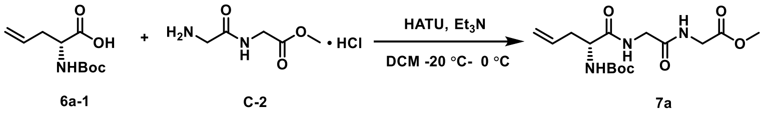

Following the General Procedure above, compound **C-2** was used instead of **C-1**, and the specific operation steps are the same as above. The resulting residue was purified by silica gel flash chromatography to give the desired product **7a** (1.35g, 84%).

7a:^{1}H NMR (600 MHz, Chloroform-*d*) δ 7.18 (s, 1H), 7.11 (s, 1H), 5.88 – 5.65 (m, 1H), 5.25 – 5.09 (m, 3H), 4.15 (q, *J* = 6.6 Hz, 1H), 4.09 – 3.96 (m, 4H), 3.75 (s, 3H), 2.64 – 2.53 (m, 1H), 2.53 – 2.42 (m, 1H), 1.44 (s, 9H).

LRMS(ESI): 343.4 $[M+H]^+$

Table S2. Glycosyl Donors 1a-1k

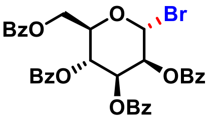

1a

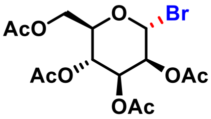

1b

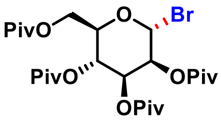

1c

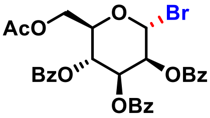

1d

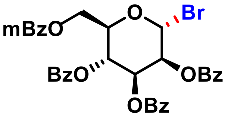

1e

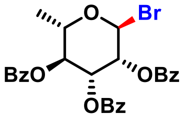

1f

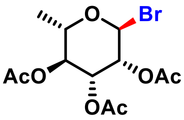

1g

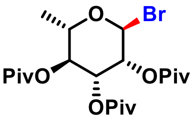

1h

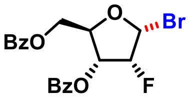

1i

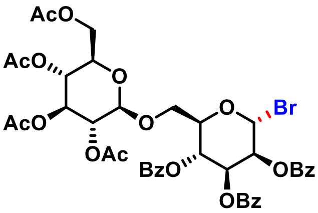

1j

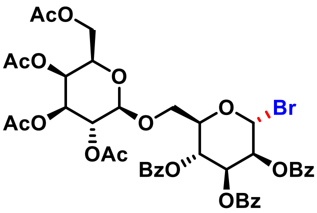

1k

Compounds **1a-1h, 1j-1k** were prepared according to literature procedures.[1] Compound **1i** were commercially available.

III. Supplementary Table of Optimization Studies

Table S3. Optimization of Reaction Conditions[a]

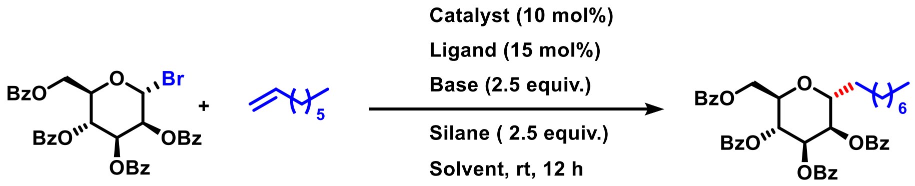

1a 2a 3aa

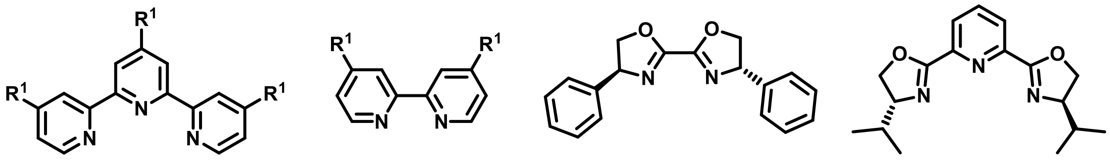

L1: R^1 = ^{t}Bu
L5: R^1 = H

L2: R^1 = ^{t}Bu

L3

L4

Entry	Catalyst	Base	Silane	Solvent	Ligand	Temp	Yield[b] (%)	α : β
1	$CoBr_2$(DME)	CsF	DEMS	1,4-Dioxane	**L1**	r.t	Trace	--
2	$CoBr_2$(DME)	CsF	DEMS	1,4-Dioxane	**L2**	r.t	Trace	--
3	$CoBr_2$(DME)	CsF	DEMS	1,4-Dioxane	**L3**	r.t	Trace	--
4	$CoBr_2$(DME)	CsF	DEMS	1,4-Dioxane	**L4**	r.t	Trace	--
5	$CoBr_2$(DME)	CsF	DEMS	1,4-Dioxane	**L5**	r.t	Trace	--
6	$NiBr_2$·diglyme	CsF	DEMS	1,4-Dioxane	**L1**	r.t.	76%	α
7	$NiBr_2$	CsF	DEMS	1,4-Dioxane	**L1**	r.t.	71%	α
8	NiI_2	CsF	DEMS	1,4-Dioxane	**L1**	r.t.	39%	α
9	$NiBr_2$·DME	CsF	DEMS	1,4-Dioxane	**L1**	r.t.	68%	α
10	$NiCl_2$	CsF	DEMS	1,4-Dioxane	**L1**	r.t.	Trace	--
11	$Cu(OAc)_2$	CsF	DEMS	1,4-Dioxane	**L1**	r.t.	Trace	--
12	$NiBr_2$·diglyme	Na_2CO_3	DEMS	1,4-Dioxane	**L1**	r.t.	16%	α
13	$NiBr_2$·diglyme	K_2CO_3	DEMS	1,4-Dioxane	**L1**	r.t.	N.R	--
14	$NiBr_2$·diglyme	K_3PO_4	DEMS	1,4-Dioxane	**L1**	r.t.	70%	α
15	$NiBr_2$·diglyme	Cs_2CO_3	DEMS	1,4-Dioxane	**L1**	r.t.	26%	α
16	$NiBr_2$·diglyme	KF	DEMS	1,4-Dioxane	**L1**	r.t.	N.R	--
17	$NiBr_2$·diglyme	K_2HPO_4	DEMS	1,4-Dioxane	**L1**	r.t.	N.R	--
18	$NiBr_2$·diglyme	KH_2PO_4	DEMS	1,4-Dioxane	**L1**	r.t.	N.R	--

19	$NiBr_2$·diglyme	CsF	PMHS	1,4-Dioxane	**L1**	r.t.	58%	α
20	$NiBr_2$·diglyme	CsF	$(EtO)_3SiH$	1,4-Dioxane	**L1**	r.t.	64%	α
21	$NiBr_2$·diglyme	CsF	$(MeO)_3SiH$	1,4-Dioxane	**L1**	r.t.	52%	α
22	$NiBr_2$·diglyme	CsF	$(MeO)_2MeSiH$	1,4-Dioxane	**L1**	r.t.	57%	α
23	$NiBr_2$·diglyme	CsF	PhSiH3	1,4-Dioxane	**L1**	r.t.	24%	α
24	$NiBr_2$·diglyme	CsF	DEMS	$PhCH_3$	**L1**	r.t.	65%	α
25	$NiBr_2$·diglyme	CsF	DEMS	THF	**L1**	r.t.	54%	α
26	$NiBr_2$·diglyme	CsF	DEMS	CH_2Cl_2	**L1**	r.t.	N.R	--
27	$NiBr_2$·diglyme	CsF	DEMS	DMF	**L1**	r.t.	Trace	--
28	$NiBr_2$·diglyme	CsF	DEMS	DMAc	**L1**	r.t.	Trace	--
29	$NiBr_2$·diglyme	CsF	DEMS	DCE	**L1**	r.t.	N.R	--
30	$NiBr_2$·diglyme	CsF	DEMS	1,4-Dioxane	**L2**	r.t.	Trace	--
31	$NiBr_2$·diglyme	CsF	DEMS	1,4-Dioxane	**L3**	r.t.	Trace	--
32	$NiBr_2$·diglyme	CsF	DEMS	1,4-Dioxane	**L4**	r.t.	Trace	--
33	$NiBr_2$·diglyme	CsF	DEMS	1,4-Dioxane	**L5**	r.t.	42%	--
34	--	CsF	DEMS	1,4-Dioxane	**L1**	r.t.	N.R	--
35	$NiBr_2$·diglyme	--	DEMS	1,4-Dioxane	**L1**	r.t.	N.R	--
36	$NiBr_2$·diglyme	CsF	--	1,4-Dioxane	**L1**	r.t.	N.R	--
37	$NiBr_2$·diglyme	CsF	DEMS	--	**L1**	r.t.	N.R	--
38	$NiBr_2$·diglyme	CsF	DEMS		**--**	r.t.	N.R	--

[a]Reactions were carried out under an argon atmosphere. Conditions: **1a** (2.0 equiv.), **2a** (1.0 equiv.), catalyst (10 mol%), ligand (15 mol%), silane (2.5 equiv.), base (2.5 equiv.), solvent (1 mL), room temperature, 12 h. [b]Isolated yield. [c]The α : β ratio was determined by ^{1}H NMR. DME = 1,2-Dimethoxyethane. DEMS = Diethoxymethylsilane. PMHS = Polymethylhydrosiloxane. DMF = *N,N*-Dimethylformamide. DMAc = *N,N*-Dimethylacetamide. Diglyme = 2-Methoxyethyl ether.

IV. General Procedures for the Preparation of *C*-alkyl Glycosides

Scheme S4. General procedures for the preparation of 3aa

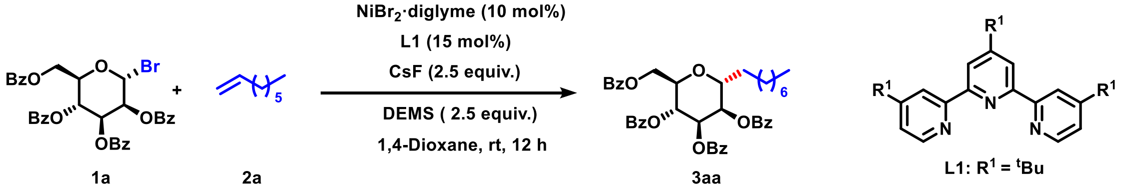

Mannosyl bromide **1a** (0.4 mmol, 2.0 equiv.), **L1** (15 mol%, 0.03 mmol), $NiBr_2$·diglyme (10 mol%, 0.02 mmol), and CsF (0.5 mmol, 2.5 equiv.) were sequentially added to a Schlenk tube under air, then the tube was evacuated and backfilled with argon three times. The mixture of 1,4-dioxane (1 mL), octene **2a** (0.2 mmol) and DEMS (0.5 mmol) was added subsequently. The resulting mixture was stirred at room temperature for 12 h. After completion of the reaction, the mixture was filtered through a celite pad and washed with DCM (2.0 mL × 3). The combined organic layer was concentrated under reduced pressure and the crude residue was purified by preparative TLC to give the desired product **3aa**.

Analytical Characterization Data of Products

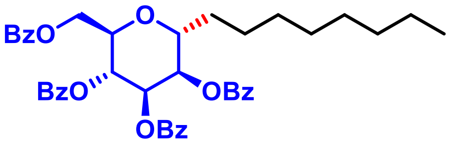

3aa

(2*R*,3*R*,4*R*,5*R*,6*R*)-2-((benzoyloxy)methyl)-6-octyltetrahydro-2*H*-pyran-3,4,5-triyl tribenzoate (3aa):

Compound **3aa** was obtained following the general procedure in 76% yield (103.2 mg) as colorless oil. (R_f = 0.6, PE/EA = 6 : 1 (v/v)).

^{1}H NMR (600 MHz, Chloroform-*d*) δ 8.13 (d, *J* = 7.9 Hz, 2H), 8.10 (d, *J* = 7.9 Hz, 2H), 8.01 (d, *J* = 7.9 Hz, 2H), 7.90 (d, *J* = 7.7 Hz, 2H), 7.60 (q, *J* = 8.3 Hz, 2H), 7.54 (t, *J* = 7.5 Hz, 1H), 7.49 – 7.38 (m, 7H), 7.32 (t, *J* = 7.7 Hz, 2H), 6.05 (t, *J* = 9.3 Hz, 1H), 5.86 (d, *J* = 9.7 Hz, 1H), 5.70 (s, 1H), 4.68 (d, *J* = 11.9 Hz, 1H), 4.62 (dd, *J* = 12.0, 5.6 Hz, 1H), 4.38 – 4.32 (m, 1H), 4.31 (d, *J* = 9.2 Hz, 1H), 2.15 – 2.04 (m, 1H), 1.86 – 1.77 (m, 1H), 1.66 – 1.55 (m, 1H), 1.55 – 1.37 (m, 3H), 1.35 – 1.25 (m, 8H), 0.92 (t, *J* = 6.9 Hz, 3H).

^{13}C NMR (150 MHz, Chloroform-*d*) δ 166.3, 165.7, 165.7, 165.5, 133.5, 133.3, 133.3, 133.1, 129.9, 129.8, 129.8, 129.7, 129.1, 128.6, 128.5, 128.4, 128.4, 75.8, 72.2, 70.3, 70.3, 67.8, 63.4, 31.9, 29.5, 29.3, 29.2, 28.6, 25.6, 22.7, 14.1.

LRMS(ESI): 715.3 $[M+Na]^+$

HRMS(ESI-TOF) m/z: $[M+H]^+$ Calcd for $C_{42}H_{45}O_9$ 693.3058; Found 693.3058.

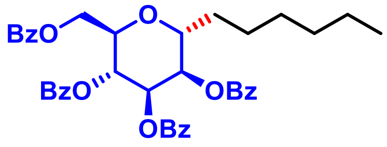

3ab

(2*R*,3*R*,4*R*,5*R*,6*R*)-2-((benzoyloxy)methyl)-6-hexyltetrahydro-2*H*-pyran-3,4,5-triyl tribenzoate (3ab)

Compound **3ab** was obtained following the general procedure in 73% yield (95.0 mg) as colorless oil. (R_f = 0.6, PE/EA = 6 : 1 (v/v)).

^{1}H NMR (600 MHz, Chloroform-*d*) δ 8.09 (d, *J* = 6.9 Hz, 2H), 8.06 (d, *J* = 6.9 Hz, 2H), 7.97 (d, *J* = 7.0 Hz, 2H), 7.86 (d, *J* = 7.0 Hz, 2H), 7.60 – 7.55 (m, 2H), 7.52 (t, *J* = 7.4 Hz, 1H), 7.45 (t, *J* = 7.5 Hz, 1H), 7.43 – 7.40 (m, 4H), 7.38 (t, *J* = 7.9 Hz, 2H), 7.29 (t, *J* = 7.9 Hz, 2H), 6.00 (t, *J* = 9.3 Hz, 1H), 5.81 (dd, *J* = 9.5, 3.3 Hz, 1H), 5.65 (t, *J* = 2.8 Hz, 1H), 4.63 (dd, *J* = 12.0, 2.8 Hz, 1H), 4.57 (dd, *J* = 12.0, 5.7 Hz, 1H), 4.32 – 4.28 (m, 1H), 4.28 – 4.24 (m, 1H), 2.09 – 2.01 (m, 1H), 1.82 – 1.74 (m, 1H), 1.50 – 1.44 (m, 1H), 1.43 – 1.34 (m, 2H), 1.32 – 1.24 (m, 5H), 0.88 (t, *J* = 6.9 Hz, 3H).

^{13}C NMR (150 MHz, Chloroform-*d*) δ 166.3, 165.7, 165.7, 165.5, 133.5, 133.3, 133.3, 133.1, 129.9, 129.8, 129.8, 129.7, 129.0, 128.5, 128.5, 128.4, 75.8, 72.1, 70.3, 67.8, 63.4, 31.7, 28.9, 28.6, 25.6, 22.5, 14.1.

LRMS(ESI): 665.3 $[M+H]^+$

HRMS(ESI-TOF) m/z: $[M+H]^+$ Calcd for $C_{40}H_{41}O_9$ 665.2745; Found 665.2742.

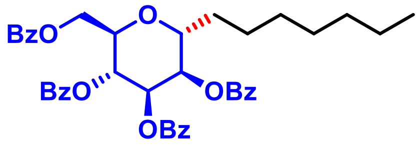

3ac

(2*R*,3*R*,4*R*,5*R*,6*R*)-2-((benzoyloxy)methyl)-6-heptyltetrahydro-2*H*-pyran-3,4,5-

triyl tribenzoate (3ac)

Compound **3ac** was obtained following the general procedure in 75% yield (99.7 mg) as colorless oil. (R_f = 0.6, PE/EA = 6 : 1 (v/v)).

^{1}H NMR (600 MHz, Chloroform-*d*) δ 8.09 (d, *J* = 7.9 Hz, 2H), 8.06 (d, *J* = 7.7 Hz, 2H), 7.98 (d, *J* = 8.0 Hz, 2H), 7.86 (d, *J* = 7.7 Hz, 2H), 7.61 – 7.55 (m, 2H), 7.52 (t, *J* = 7.7 Hz, 1H), 7.46 (t, *J* = 7.3 Hz, 1H), 7.44 – 7.40 (m, 4H), 7.38 (t, *J* = 7.8 Hz, 2H), 7.30 (t, *J* = 7.0 Hz, 2H), 6.00 (t, *J* = 9.9 Hz, 1H), 5.82 (dt, *J* = 9.6, 2.7 Hz, 1H), 5.66 (s, 1H), 4.64 (d, *J* = 12.1 Hz, 1H), 4.58 (dd, *J* = 12.4, 5.5 Hz, 1H), 4.33 – 4.29 (m, 1H), 4.29 – 4.25 (m, 1H), 2.09 – 2.01 (m, 1H), 1.82 – 1.75 (m, 1H), 1.65 – 1.53 (m, 2H), 1.51 – 1.44 (m, 1H), 1.44 – 1.34 (m, 2H), 1.31 – 1.27 (m, 5H), 0.88 (t, *J* = 7.0, 2.3 Hz, 3H).

^{13}C NMR (150 MHz, Chloroform-*d*) δ 166.2, 165.7, 165.7, 165.5, 133.5, 133.3, 133.3, 133.1, 129.9, 129.8, 129.8, 129.7, 129.0, 128.6, 128.5, 128.5, 128.4, 75.8, 72.1, 70.3, 67.8, 63.4, 31.7, 29.2, 28.6, 25.6, 22.6, 14.1.

LRMS(ESI): 679.7 $[M+H]^+$; 701.4 $[M+Na]^+$

HRMS(ESI-TOF) m/z: $[M+H]^+$ Calcd for $C_{41}H_{43}O_9$ 679.2902; Found 679.2904.

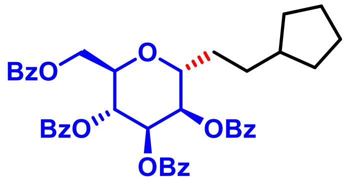

3ad

(2*R*,3*R*,4*R*,5*R*,6*R*)-2-((benzoyloxy)methyl)-6-(2-cyclopentylethyl)tetrahydro-2*H*-pyran-3,4,5-triyl tribenzoate (3ad)

Compound **3ad** was obtained following the general procedure in 52% yield (68.9 mg) as colorless oil. (R_f = 0.6, PE/EA = 6 : 1 (v/v)).

^{1}H NMR (600 MHz, Chloroform-*d*) δ 8.09 (d, *J* = 6.9 Hz, 2H), 8.06 (d, *J* = 6.9 Hz, 2H), 7.98 (d, *J* = 7.0 Hz, 2H), 7.86 (d, *J* = 6.9 Hz, 2H), 7.60 – 7.55 (m, 2H), 7.52 (t, *J* = 7.4 Hz, 1H), 7.45 (t, *J* = 7.5 Hz, 1H), 7.44 – 7.40 (m, 4H), 7.38 (t, *J* = 7.8 Hz, 2H), 7.29 (t, *J* = 7.9 Hz, 2H), 6.00 (t, *J* = 9.4 Hz, 1H), 5.82 (dd, *J* = 9.6, 3.3 Hz, 1H), 5.66 (dd, *J* = 3.4, 2.3 Hz, 1H), 4.64 (dd, *J* = 11.9, 2.8 Hz, 1H), 4.56 (dd, *J* = 12.0, 5.9 Hz, 1H), 4.32 – 4.28 (m, 1H), 4.27 – 4.23 (m, 1H), 2.09 – 2.02 (m, 1H), 1.88 – 1.83 (m, 1H), 1.82 – 1.76 (m, 3H), 1.60 – 1.56 (m, 3H), 1.55 – 1.50 (m, 2H), 1.50 – 1.44 (m, 1H), 1.13 –

1.05 (m, 2H).

^{13}C NMR (150 MHz, Chloroform-*d*) δ 166.2, 165.8, 165.7, 165.5, 133.5, 133.3, 133.3, 133.1, 129.9, 129.8, 129.8, 129.8, 129.7, 129.0, 128.5, 128.5, 128.4, 128.4, 76.1, 72.2, 70.3, 70.2, 67.8, 63.4, 39.7, 32.8, 32.6, 32.0, 27.7, 25.2.

LRMS(ESI): 699.3 $[M+Na]^+$

HRMS(ESI-TOF) m/z: $[M+H]^+$ Calcd for $C_{41}H_{41}O_9$ 677.2745; Found 677.2742.

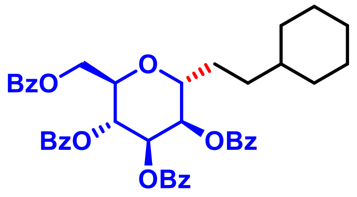

3ae

(2*R*,3*R*,4*R*,5*R*,6*R*)-2-((benzoyloxy)methyl)-6-(2-cyclohexylethyl)tetrahydro-2*H*-pyran-3,4,5-triyl tribenzoate (3ae)

Compound **3ae** was obtained following the general procedure in 56% yield (75.8 mg) as colorless oil. (R_f = 0.6, PE/EA = 6 : 1 (v/v)).

^{1}H NMR (600 MHz, Chloroform-*d*) δ 8.10 (d, *J* = 6.9 Hz, 2H), 8.06 (d, *J* = 6.9 Hz, 2H), 7.98 (d, *J* = 7.0 Hz, 2H), 7.86 (d, *J* = 7.0 Hz, 2H), 7.61 – 7.55 (m, 2H), 7.52 (t, *J* = 7.4 Hz, 1H), 7.47 – 7.36 (m, 7H), 7.29 (t, *J* = 7.8 Hz, 2H), 6.00 (t, *J* = 9.4 Hz, 1H), 5.81 (dd, *J* = 9.6, 3.3 Hz, 1H), 5.68 – 5.64 (m, 1H), 4.64 (dd, *J* = 11.9, 2.8 Hz, 1H), 4.56 (dd, *J* = 12.0, 5.8 Hz, 1H), 4.29 (ddd, *J* = 8.9, 5.8, 2.8 Hz, 1H), 4.22 (ddd, *J* = 10.6, 4.8, 2.3 Hz, 1H), 2.05 – 1.99 (m, 1H), 1.85 – 1.76 (m, 1H), 1.76 – 1.63 (m, 5H), 1.51 – 1.44 (m, 1H), 1.38 – 1.32 (m, 1H), 1.32 – 1.28 (m, 1H), 1.24 – 1.19 (m, 2H), 1.18 – 1.11 (m, 1H), 0.96 – 0.82 (m, 2H).

^{13}C NMR (150 MHz, Chloroform-*d*) δ 166.2, 165.8, 165.7, 165.5, 133.5, 133.3, 133.3, 133.1, 129.9, 129.8, 129.8, 129.8, 129.7, 129.0, 128.5, 128.5, 128.4, 128.4, 76.2, 72.2, 70.3, 70.2, 67.8, 63.4, 37.3, 33.4, 33.2, 33.1, 26.6, 26.3, 26.3, 25.9.

LRMS(ESI): 713.6 $[M+Na]^+$

HRMS(ESI-TOF) m/z: $[M+H]^+$ Calcd for $C_{42}H_{43}O_9$ 691.2902; Found 691.2901.

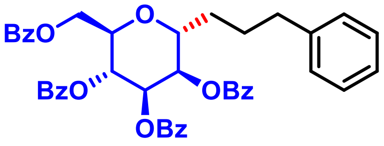

3af

(2*R*,3*R*,4*R*,5*R*,6*R*)-2-((benzoyloxy)methyl)-6-(3-phenylpropyl)tetrahydro-2*H*-pyran-3,4,5-triyl tribenzoate (3af)

Compound **3af** was obtained following the general procedure in 64% yield (87.6 mg) as colorless oil. (R_f= 0.6, PE/EA = 6 : 1 (v/v)).

^{1}H NMR (600 MHz, Chloroform-*d*) δ 8.08 (d, *J* = 7.8 Hz, 4H), 8.00 (d, *J* = 7.8 Hz, 2H), 7.89 (d, *J* = 7.8 Hz, 2H), 7.61 (t, *J* = 7.5 Hz, 1H), 7.59 – 7.53 (m, 2H), 7.48 (t, *J* = 7.6 Hz, 1H), 7.44 (t, *J* = 7.9 Hz, 2H), 7.42 – 7.37 (m, 4H), 7.32 (t, *J* = 8.1 Hz, 2H), 7.29 (d, *J* = 8.0 Hz, 2H), 7.22 (d, *J* = 7.4 Hz, 1H), 7.19 (d, *J* = 7.8 Hz, 2H), 6.01 (t, *J* = 9.1 Hz, 1H), 5.81 (dd, *J* = 9.4, 3.1 Hz, 1H), 5.67 (t, J = 2.9 Hz, 1H), 4.64 (dd, *J* = 12.0, 2.7 Hz, 1H), 4.60 (dd, *J* = 12.1, 5.6 Hz, 1H), 4.35 – 4.31 (m, 1H), 4.28 – 4.24 (m, 1H), 2.80 – 2.69 (m, 2H), 2.17 – 2.06 (m, 1H), 2.01 – 1.90 (m, 1H), 1.89 – 1.78 (m, 2H).

^{13}C NMR (150 MHz, Chloroform-*d*) δ 166.3, 165.7, 165.7, 165.5, 141.6, 133.5, 133.4, 133.3, 133.1, 129.8, 129.8, 129.7, 129.6, 129.0, 128.6, 128.5, 128.4, 128.4, 75.4, 72.0, 70.4, 70.2, 67.8, 63.3, 35.3, 28.0, 27.2.

LRMS(ESI):721.3 $[M+Na]^+$

HRMS(ESI-TOF) m/z: $[M+H]^+$ Calcd for $C_{43}H_{39}O_9$ 699.2589; Found 699.2589.

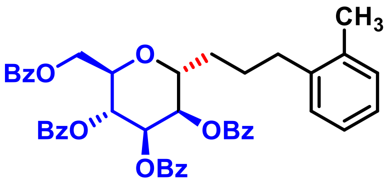

3ag

(2*R*,3*R*,4*R*,5*R*,6*R*)-2-((benzoyloxy)methyl)-6-(3-(o-tolyl)propyl)tetrahydro-2*H*-pyran-3,4,5-triyl tribenzoate (3ag)

Compound **3ag** was obtained following the general procedure in 70% yield (97.8 mg) as colorless oil. (R_f= 0.6, PE/EA = 6 : 1 (v/v)).

^{1}H NMR (600 MHz, Chloroform-*d*) δ 8.05 (dd, *J* = 11.1, 7.8 Hz, 4H), 7.99 (d, *J* = 7.8

Hz, 2H), 7.88 (d, *J* = 7.8 Hz, 2H), 7.59 (t, *J* = 7.5 Hz, 1H), 7.54 (q, *J* = 8.2 Hz, 2H), 7.46 (t, *J* = 7.5 Hz, 1H), δ 7.44 – 7.33 (m, 6H), 7.31 (t, *J* = 7.7 Hz, 2H), 7.12 (s, 4H), 6.00 (t, *J* = 9.1 Hz, 1H), 5.82 (dd, *J* = 9.5, 3.2 Hz, 1H), 5.67 (t, *J* = 2.9 Hz, 1H), 4.62 (dd, *J* = 12.1, 2.9 Hz, 1H), 4.58 (dd, *J* = 12.0, 5.5 Hz, 1H), 4.35 – 4.31 (m, 1H), 4.29 – 4.25 (m, 1H), 2.78 – 2.64 (m, 2H), 2.30 (s, 3H), 2.19 – 2.11 (m, 1H), 1.93 – 1.84 (m, 2H), 1.82 – 1.76 (m, 1H).

^{13}C NMR (150 MHz, Chloroform-*d*) δ 166.3, 165.7, 165.7, 165.5, 139.9, 135.9, 133.5, 133.4, 133.3, 133.0, 130.3, 129.8, 129.8, 129.7, 129.6, 129.0, 128.7, 128.6, 128.5, 128.4, 126.1, 126.0, 75.4, 72.1, 70.4, 70.2, 67.8, 63.3, 32.7, 28.4, 26.0, 19.3.

LRMS(ESI): 713.6 [M+H]$^+$; 735.3 [M+Na]$^+$

HRMS(ESI-TOF) m/z: [M+H]$^+$ Calcd for $C_{44}H_{41}O_9$ 713.2745; Found 713.2743.

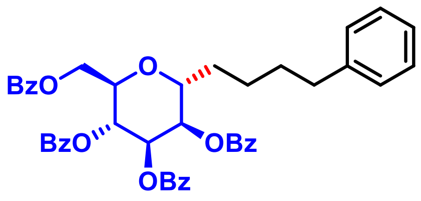

3ah

(2*R*,3*R*,4*R*,5*R*,6*R*)-2-((benzoyloxy)methyl)-6-(4-phenylbutyl)tetrahydro-2*H*-pyran-3,4,5-triyl tribenzoate (3ah)

Compound **3ah** was obtained following the general procedure in 67% yield (93.6 mg) as colorless oil. (R_f = 0.6, PE/EA = 6 : 1 (v/v)).

^{1}H NMR (600 MHz, Chloroform-*d*) δ 8.14 (d, *J* = 7.9 Hz, 2H), 8.09 (d, *J* = 7.9 Hz, 2H), 8.02 (d, *J* = 7.9 Hz, 2H), 7.91 (d, *J* = 7.8 Hz, 2H), 7.61 (q, *J* = 7.9 Hz, 2H), 7.55 (t, *J* = 7.4 Hz, 1H), 7.49 – 7.40 (m, 8H), 7.35 – 7.29 (m, 4H), 7.23 – 7.17 (m, 2H), 6.04 (t, *J* = 9.2 Hz, 1H), 5.85 (dd, *J* = 9.4, 3.2 Hz, 1H), 5.69 (t, *J* = 2.9 Hz, 1H), 4.66 (dd, *J* = 12.1, 3.2 Hz, 1H), 4.62 (dd, *J* = 12.1, 5.4 Hz, 1H), 4.34 – 4.28 (m, 2H), 2.68 – 2.60 (m, 2H), 2.15 – 2.09 (m, 1H), 1.87 – 1.71 (m, 3H), 1.70 – 1.62 (m, 1H), 1.60 – 1.52 (m, 1H).

^{13}C NMR (150 MHz, Chloroform-*d*) δ 166.2, 165.7, 165.7, 165.5, 142.2, 133.5, 133.4, 133.3, 133.1, 129.9, 129.8, 129.8, 129.8, 129.7, 129.0, 128.6, 128.5, 128.4, 128.4, 128.4, 125.8, 75.6, 72.1, 70.3, 70.2, 67.8, 63.3, 35.8, 31.0, 28.5, 25.3.

LRMS(ESI): 713.5 [M+H]$^+$; 735.3 [M+Na]$^+$

HRMS(ESI-TOF) m/z: $[M+H]^+$ Calcd for $C_{44}H_{41}O_9$ 713.2745; Found 713.2749.

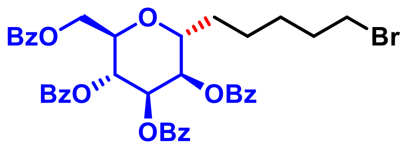

3ai

(2*R*,3*R*,4*R*,5*R*,6*R*)-2-((benzoyloxy)methyl)-6-(5-bromopentyl)tetrahydro-2*H*-pyran-3,4,5-triyl tribenzoate (3ai)

Compound **3ai** was obtained following the general procedure in 72% yield (103.0 mg) as colorless oil. (R_f = 0.6, PE/EA = 6 : 1 (v/v)).

^{1}H NMR (600 MHz, Chloroform-*d*) δ 8.09 (d, *J* = 7.8 Hz, 2H), 8.05 (d, *J* = 7.8 Hz, 2H), 7.99 (d, *J* = 7.8 Hz, 2H), 7.87 (d, *J* = 7.7 Hz, 2H), 7.58 (q, *J* = 7.5 Hz, 2H), 7.52 (t, *J* = 7.3 Hz, 1H), 7.46 (t, *J* = 7.5 Hz, 1H), 7.44 – 7.36 (m, 6H), 7.31 (t, *J* = 7.7 Hz, 2H), 5.98 (t, *J* = 9.1 Hz, 1H), 5.81 (dd, *J* = 9.3, 3.2 Hz, 1H), 5.64 (t, *J* = 2.9 Hz, 1H), 4.65 – 4.61 (m, 2H), 4.33 – 4.29 (m, 1H), 4.29 – 4.25 (m, 1H), 3.37 (t, *J* = 6.8 Hz, 2H), 2.10 – 2.01 (m, 1H), 1.86 – 1.76 (m, 3H), 1.58 – 1.45 (m, 4H).

^{13}C NMR (150 MHz, Chloroform-*d*) δ 166.2, 165.7, 165.7, 165.5, 133.5, 133.4, 133.4, 133.2, 129.8, 129.8, 129.7, 129.6, 129.0, 129.0, 128.6, 128.5, 128.5, 128.4, 75.3, 71.9, 70.5, 70.1, 67.8, 63.2, 33.5, 32.6, 28.6, 27.8, 24.8.

LRMS(ESI): 751.4 $[M+Na]^+$

HRMS(ESI-TOF) m/z: $[M+H]^+$ Calcd for $C_{39}H_{38}BrO_9$ 729.1694; Found 729.1694.

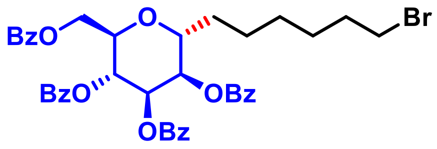

3aj

(2*R*,3*R*,4*R*,5*R*,6*R*)-2-((benzoyloxy)methyl)-6-(6-bromohexyl)tetrahydro-2*H*-pyran-3,4,5-triyl tribenzoate (3aj)

Compound **3aj** was obtained following the general procedure in 68% yield (99.2 mg) as colorless oil. (R_f = 0.6, PE/EA = 6 : 1 (v/v)).

^{1}H NMR (600 MHz, Chloroform-*d*) δ 8.09 (d, *J* = 7.4 Hz, 2H), 8.05 (d, *J* = 7.7 Hz, 2H), 7.98 (d, *J* = 7.4 Hz, 2H), 7.87 (d, *J* = 7.3 Hz, 2H), 7.58 (q, *J* = 7.5 Hz, 2H), 7.52 (t, *J* =

7.5 Hz, 1H), 7.46 (t, J = 7.5 Hz, 1H), 7.44 – 7.40 (m, 4H), 7.38 (t, J = 7.7 Hz, 2H), 7.30 (t, J = 7.7 Hz, 2H), 5.99 (t, J = 9.2 Hz, 1H), 5.80 (dd, J = 9.4, 3.3 Hz, 1H), 5.64 (t, J = 2.9 Hz, 1H), 4.63 (dd, J = 12.1, 3.3 Hz, 1H), 4.60 (dd, J = 12.5, 5.9 Hz, 1H), 4.32 – 4.29 (m, 1H), 4.28 – 4.24 (m, 1H), 3.38 (t, J = 6.8 Hz, 2H), 2.10 – 2.01 (m, 1H), 1.86 – 1.75 (m, 3H), 1.63 – 1.56 (m, 3H), 1.47 – 1.42 (m, 3H).

^{13}C NMR (150 MHz, Chloroform-*d*) δ 166.3, 165.7, 165.7, 165.5, 133.5, 133.4, 133.4, 133.2, 129.8, 129.8, 129.6, 129.0, 129.0, 128.6, 128.5, 128.5, 128.4, 75.5, 72.0, 70.4, 70.2, 67.7, 63.3, 33.9, 32.6, 28.5, 28.4, 28.1, 25.4.

LRMS(ESI): 765.2 $[M+Na]^+$

HRMS(ESI-TOF) m/z: $[M+H]^+$ Calcd for $C_{40}H_{40}BrO_9$ 743.185; Found 743.1846.

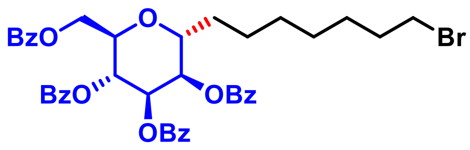

3ak

(2*R*,3*R*,4*R*,5*R*,6*R*)-2-((benzoyloxy)methyl)-6-(7-bromoheptyl)tetrahydro-2*H*-pyran-3,4,5-triyl tribenzoate (3ak)

Compound **3ak** was obtained following the general procedure in 64% yield (95.2 mg) as colorless oil. (R_f = 0.6, PE/EA = 6 : 1 (v/v)).

^{1}H NMR (600 MHz, Chloroform-*d*) δ 8.09 (d, J = 7.9 Hz, 2H), 8.06 (d, J = 7.8 Hz, 2H), 7.98 (d, J = 7.8 Hz, 2H), 7.87 (d, J = 7.8 Hz, 2H), 7.58 (q, J = 8.0 Hz, 2H), 7.52 (t, J = 7.5 Hz, 1H), 7.45 – 7.38 (m, 7H), 7.30 (t, J = 7.6 Hz, 2H), 6.00 (t, J = 9.2 Hz, 1H), 5.82 (dd, J = 9.5, 3.2 Hz, 1H), 5.65 (t, J = 2.9 Hz, 1H), 4.64 (dd, J = 12.0, 2.9 Hz, 1H), 4.60 (dd, J = 12.0, 5.6 Hz, 1H), 4.33 – 4.29 (m, 1H), 4.29 – 4.25 (m, 1H), 3.39 (t, J = 6.9 Hz, 2H), 2.08 – 2.01 (m, 1H), 1.87 – 1.74 (m, 3H), 1.61 – 1.54 (m, 1H), 1.49 – 1.39 (m, 5H), 1.33 – 1.28 (m, 2H).

^{13}C NMR (150 MHz, Chloroform-*d*) δ 166.2, 165.7, 165.7, 165.5, 133.5, 133.4, 133.3, 133.1, 129.9, 129.8, 129.8, 129.7, 129.0, 129.0, 128.6, 128.5, 128.4, 128.4, 75.6, 72.1, 70.3, 70.2, 67.8, 63.3, 33.9, 32.8, 29.0, 28.6, 28.6, 28.0, 25.5.

LRMS(ESI): 779.3 $[M+Na]^+$

HRMS(ESI-TOF) m/z: $[M+H]^+$ Calcd for $C_{41}H_{42}BrO_9$ 757.2007; Found 757.2004.

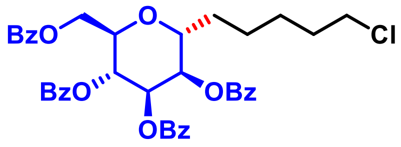

3al

(2*R*,3*R*,4*R*,5*R*,6*R*)-2-((benzoyloxy)methyl)-6-(5-chloropentyl)tetrahydro-2*H*-pyran-3,4,5-triyl tribenzoate (3al)

Compound **3al** was obtained following the general procedure in 67% yield (89.9 mg) as colorless oil. (R_f= 0.6, PE/EA = 6 : 1 (v/v)).

^{1}H NMR (500 MHz, Chloroform-*d*) δ 8.11 (d, *J* = 7.7 Hz, 2H), 8.08 (d, *J* = 7.8 Hz, 2H), 8.01 (d, *J* = 7.8 Hz, 2H), 7.90 (d, *J* = 7.7 Hz, 2H), 7.61 (q, *J* = 7.3 Hz, 2H), 7.55 (t, *J* = 7.4 Hz, 1H), 7.52 – 7.38 (m, 7H), 7.33 (t, *J* = 7.7 Hz, 2H), 6.01 (t, *J* = 9.1 Hz, 1H), 5.83 (dd, *J* = 9.3, 3.3 Hz, 1H), 5.67 (t, *J* = 2.7 Hz, 1H), 4.65 (d, *J* = 4.3 Hz, 2H), 4.36 – 4.27 (m, 2H), 3.52 (t, *J* = 6.6 Hz, 2H), 2.16 – 2.04 (m, 1H), 1.87 – 1.72 (m, 3H), 1.68 – 1.48 (m, 4H).

^{13}C NMR (125 MHz, Chloroform-*d*) δ 166.2, 165.7, 165.7, 165.5, 133.5, 133.4, 133.4, 133.2, 129.8, 129.8, 129.7, 129.6, 129.0, 128.6, 128.5, 128.5, 128.4, 75.4, 71.9, 70.4, 70.1, 67.8, 63.2, 44.8, 32.5, 28.6, 26.5, 24.9.

LRMS(ESI): 701.9 $[M+NH_4]^+$

HRMS(ESI-TOF) m/z: $[M+Na]^+$ Calcd for $C_{39}H_{37}ClNaO_9$ 707.2017; Found 707.2018.

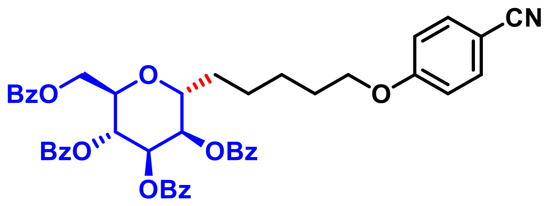

3am

(2*R*,3*R*,4*R*,5*R*,6*R*)-2-((benzoyloxy)methyl)-6-(5-(4-cyanophenoxy)pentyl)tetrahydro-2*H*-pyran-3,4,5-triyl tribenzoate (3am)

Compound **3am** was obtained following the general procedure in 59% yield (88.9 mg) as colorless oil. (R_f= 0.6, PE/EA = 1 : 1 (v/v)).

^{1}H NMR (500 MHz, Acetone-*d_6*) δ 8.11 (dd, *J* = 8.2, 1.4 Hz, 2H), 8.05 (dd, *J* = 8.3, 1.4 Hz, 2H), 8.00 (dd, *J* = 8.2, 1.4 Hz, 2H), 7.83 (dd, *J* = 8.3, 1.4 Hz, 2H), 7.71 – 7.63 (m,

4H), 7.62 – 7.55 (m, 1H), 7.56 – 7.46 (m, 5H), 7.44 (t, *J* = 7.8 Hz, 2H), 7.39 – 7.32 (m, 2H), 7.13 – 7.05 (m, 2H), 6.06 (t, *J* = 9.3 Hz, 1H), 5.92 (dd, *J* = 9.5, 3.3 Hz, 1H), 5.76 – 5.70 (m, 1H), 4.71 – 4.60 (m, 2H), 4.59 – 4.51 (m, 1H), 4.31 (ddd, *J* = 10.6, 4.6, 2.4 Hz, 1H), 4.12 (t, *J* = 6.5 Hz, 2H), 2.33 – 2.22 (m, 1H), 1.96 – 1.82 (m, 3H), 1.77 – 1.55 (m, 4H).

^{13}C NMR (125 MHz, Acetone- *d*$_6$) δ 165.1, 164.7, 164.7, 162.1, 133.5, 133.0, 133.0, 132.9, 132.7, 129.7, 129.5, 129.1, 129.0, 129.0, 128.9, 128.9, 128.2, 128.1, 128.1, 128.0, 118.3, 115.0, 103.0, 75.1, 71.7, 70.2, 69.6, 67.7, 67.2, 62.5, 27.6, 24.9, 24.8.

LRMS(ESI): 790.3 [M+Na]$^+$

HRMS(ESI-TOF) m/z: [M+H]$^+$ Calcd for $C_{46}H_{42}NO_{10}$ 768.2803; Found 768.2803.

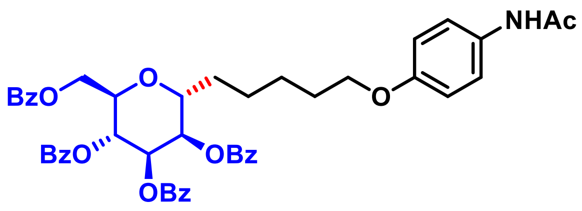

3an

(2*R*,3*R*,4*R*,5*R*,6*R*)-2-(5-(4-acetamidophenoxy)pentyl)-6-((benzoyloxy)methyl)tetrahydro-2*H*-pyran-3,4,5-triyl tribenzoate (3an)

Compound **3an** was obtained following the general procedure in 69% yield (108.4 mg) as colorless oil. (R_f = 0.5, PE/EA = 1 : 1 (v/v)).

^{1}H NMR (500 MHz, Acetone-*d*$_6$) δ 8.12 (d, *J* = 6.9 Hz, 2H), 8.06 (d, *J* = 6.9 Hz, 2H), 8.01 (d, *J* = 6.9 Hz, 2H), 7.84 (d, *J* = 7.0 Hz, 2H), 7.69 – 7.65 (m, 2H), 7.59 (t, *J* = 7.4 Hz, 1H), 7.55 – 7.48 (m, 7H), 7.45 (t, *J* = 7.8 Hz, 2H), 7.38 – 7.34 (m, 2H), 6.85 (d, *J* = 9.0 Hz, 2H), 6.08 (t, *J* = 9.3 Hz, 1H), 5.93 (dd, *J* = 9.5, 3.3 Hz, 1H), 5.75 (t, *J* = 2.8 Hz, 1H), 4.71 – 4.62 (m, 2H), 4.60 – 4.55 (m, 1H), 4.35 – 4.30 (m, 1H), 3.97 (t, *J* = 6.4 Hz, 2H), 2.33 – 2.24 (m, 1H), 2.03 (s, 3H), 1.95 – 1.88 (m, 1H), 1.84 – 1.77 (m, 2H), 1.74 – 1.69 (m, 1H), 1.67 – 1.60 (m, 2H), 1.60 – 1.54 (m, 1H).

^{13}C NMR (125 MHz, Acetone-*d*$_6$) δ 165.1, 164.7, 164.7, 164.6, 154.7, 133.0, 132.9, 132.9, 132.7, 129.7, 129.5, 129.1, 129.0, 129.0, 128.9, 128.8, 128.2, 128.1, 128.1, 128.0, 120.1, 120.0, 113.9, 75.2, 71.7, 70.2, 69.6, 67.2, 67.1, 62.5, 27.6, 25.0, 24.8, 22.7, 22.6.

LRMS(ESI): 800.2 [M+H]$^+$; 822.4 [M+Na]$^+$

HRMS(ESI-TOF) m/z: $[M+Na]^+$ Calcd for $C_{47}H_{45}NNaO_{11}$ 822.2885; Found 822.2885.

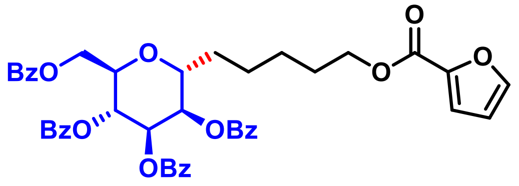

3ao

(2*R*,3*R*,4*R*,5*R*,6*R*)-2-((benzoyloxy)methyl)-6-(5-((furan-2-carbonyl)oxy)pentyl)tetrahydro-2*H*-pyran-3,4,5-triyl tribenzoate (3ao)

Compound **3ao** was obtained following the general procedure in 61% yield (91.1 mg) as colorless oil. (R_f = 0.4, PE/EA = 3 : 1 (v/v)).

^{1}H NMR (500 MHz, Chloroform-*d*) δ 8.08 (d, *J* = 7.2 Hz, 2H), 8.05 (d, *J* = 7.1 Hz, 2H), 7.98 (d, *J* = 7.1 Hz, 2H), 7.86 (d, *J* = 7.2 Hz, 2H), 7.62 – 7.49 (m, 4H), 7.49 – 7.35 (m, 7H), 7.30 (t, *J* = 7.7 Hz, 2H), 7.18 (d, *J* = 3.5 Hz, 1H), 6.50 (dd, *J* = 3.5, 1.7 Hz, 1H), 5.99 (t, *J* = 9.2 Hz, 1H), 5.80 (dd, *J* = 9.4, 3.3 Hz, 1H), 5.64 (t, *J* = 2.9 Hz, 1H), 4.65 – 4.58 (m, 2H), 4.33 – 4.24 (m, 4H), 2.12 – 2.01 (m, 1H), 1.85 – 1.76 (m, 1H), 1.77 – 1.69 (m, 2H), 1.59 – 1.47 (m, 4H).

^{13}C NMR (125 MHz, Chloroform-*d*) δ 165.8, 165.3, 165.0, 158.4, 145.8, 144.4, 133.0, 132.9, 132.9, 132.7, 129.4, 129.3, 129.3, 129.1, 128.5, 128.5, 128.1, 128.0, 128.0, 127.9, 117.4, 111.4, 75.0, 71.5, 69.9, 69.7, 67.3, 64.3, 62.7, 28.2, 28.1, 25.2, 24.9.

LRMS(ESI): 783.4 $[M+Na]^+$

HRMS(ESI-TOF) m/z: $[M+H]^+$ Calcd for $C_{44}H_{41}O_{12}$ 761.2593; Found 761.2596.

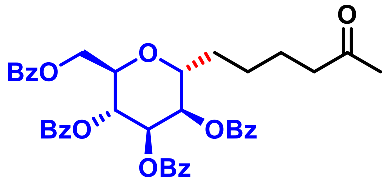

3ap

(2*R*,3*R*,4*R*,5*R*,6*R*)-2-((benzoyloxy)methyl)-6-(5-oxohexyl)tetrahydro-2*H*-pyran-3,4,5-triyl tribenzoate (3ap)

Compound **3ap** was obtained following the general procedure in 55% yield (73.1 mg) as colorless oil. (R_f = 0.4, PE/EA = 4 : 1 (v/v)).

^{1}H NMR (500 MHz, Chloroform-*d*) δ 8.11 (d, *J* = 7.3 Hz, 2H), 8.07 (d, *J* = 7.2 Hz, 2H), 8.01 (d, *J* = 7.3 Hz, 2H), 7.89 (d, *J* = 7.3 Hz, 2H), 7.60 (dd, *J* = 7.6, 6.1 Hz, 2H), 7.55 (t, *J* = 7.4 Hz, 1H), 7.51 – 7.38 (m, 7H), 7.33 (t, *J* = 7.8 Hz, 2H), 6.02 (t, *J* = 9.1 Hz, 1H), 5.82 (dd, *J* = 9.3, 3.3 Hz, 1H), 5.66 (t, *J* = 3.0 Hz, 1H), 4.69 – 4.59 (m, 2H), 4.37 – 4.23 (m, 2H), 2.43 (t, *J* = 7.3 Hz, 2H), 2.14 (s, 3H), 2.11 – 2.00 (m, 1H), 1.88 – 1.77 (m, 1H), 1.75 – 1.68 (m, 2H), 1.64 – 1.52 (m, 1H), 1.53 – 1.41 (m, 1H).

^{13}C NMR (125 MHz, Chloroform-*d*) δ 208.5, 166.2, 165.7, 165.7, 165.5, 133.5, 133.4, 133.4, 133.1, 129.8, 129.8, 129.6, 129.0, 128.6, 128.5, 128.4, 128.4, 75.3, 71.9, 70.4, 70.1, 67.7, 63.2, 43.4, 29.9, 28.5, 25.1, 23.2.

LRMS(ESI): 701.3 $[M+Na]^+$

HRMS(ESI-TOF) m/z: $[M+H]^+$ Calcd for $C_{40}H_{39}O_{10}$ 679.2538; Found 679.2537.

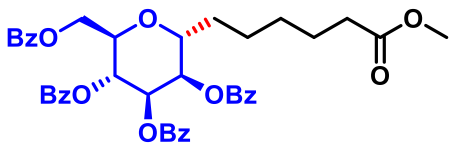

3aq

(2*R*,3*R*,4*R*,5*R*,6*R*)-2-((benzoyloxy)methyl)-6-(6-methoxy-6-oxohexyl)tetrahydro-2*H*-pyran-3,4,5-triyl tribenzoate (3aq)

Compound **3aq** was obtained following the general procedure in 58% yield (80.6 mg) as colorless oil. (R_f = 0.3, PE/EA = 4 : 1 (v/v)).

^{1}H NMR (600 MHz, Chloroform-*d*) δ 8.09 (d, *J* = 8.1 Hz, 2H), 8.05 (d, *J* = 8.1 Hz, 2H), 7.98 (d, *J* = 7.7 Hz, 2H), 7.87 (d, *J* = 8.0 Hz, 2H), 7.61 – 7.54 (m, 2H), 7.52 (t, *J* = 7.6 Hz, 1H), 7.47 – 7.35 (m, 7H), 7.30 (t, *J* = 7.6 Hz, 2H), 6.00 (t, *J* = 9.2 Hz, 1H), 5.81 (dd, *J* = 9.4, 3.2 Hz, 1H), 5.64 (t, *J* = 2.9 Hz, 1H), 4.65 – 4.58 (m, 2H), 4.32 – 4.29 (m, 1H), 4.28 – 4.25 (m, 1H), 3.67 (s, 3H), 2.30 (t, *J* = 7.5 Hz, 2H), 2.09 – 2.00 (m, 1H), 1.83 – 1.75 (m, 1H), 1.64 – 1.57 (m, 3H), 1.49 – 1.38 (m, 3H).

^{13}C NMR (150 MHz, Chloroform-*d*) δ 174.0, 166.2, 165.7, 165.7, 165.5, 133.5, 133.4, 133.3, 133.1, 129.9, 129.8, 129.8, 129.7, 129.6, 129.0, 128.5, 128.5, 128.4, 128.4, 75.5, 72.0, 70.4, 70.2, 67.8, 63.3, 51.5, 33.9, 28.7, 28.5, 25.3, 24.8.

LRMS(ESI): 731.3 $[M+Na]^+$

HRMS(ESI-TOF) m/z: $[M+H]^+$ Calcd for $C_{41}H_{41}O_{11}$ 709.2643; Found 709.2646.

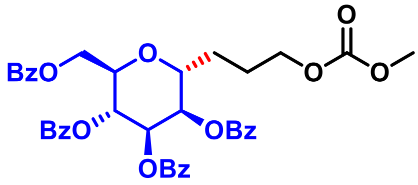

3ar

(2*R*,3*R*,4*R*,5*R*,6*R*)-2-((benzoyloxy)methyl)-6-(3-((methoxycarbonyl)oxy)propyl)tetrahydro-2*H*-pyran-3,4,5-triyl tribenzoate (3ar)

Compound **3ar** was obtained following the general procedure in 35% yield (44.8 mg) as colorless oil. (R_f= 0.5, PE/EA = 2 : 1 (v/v)).

^{1}H NMR (600 MHz, Chloroform-*d*) δ 8.10 (d, *J* = 7.8 Hz, 2H), 8.06 (d, *J* = 7.8 Hz, 2H), 8.01 (d, *J* = 7.9 Hz, 2H), 7.90 (d, *J* = 7.9 Hz, 2H), 7.60 (q, *J* = 8.1 Hz, 2H), 7.56 (t, *J* = 7.3 Hz, 1H), 7.49 (t, *J* = 7.5 Hz, 1H), 7.46 – 7.40 (m, 6H), 7.34 (t, *J* = 7.7 Hz, 2H), 6.02 (t, *J* = 8.9 Hz, 1H), 5.83 (dd, *J* = 9.2, 3.2 Hz, 1H), 5.66 (t, *J* = 3.3 Hz, 1H), 4.72 – 4.61 (m, 2H), 4.36 – 4.30 (m, 2H), 4.27 (t, *J* = 6.0 Hz, 2H), 3.81 (s, 3H), 2.21 – 2.12 (m, 1H), 2.03 – 1.96 (m, 1H), 1.95 – 1.84 (m, 2H).

^{13}C NMR (150 MHz, Chloroform-*d*) δ 166.2, 165.7, 165.5, 155.8, 133.6, 133.4, 133.4, 133.1, 129.9, 129.8, 129.8, 129.8, 129.5, 129.0, 128.9, 128.6, 128.5, 128.5, 128.5, 74.9, 71.8, 70.6, 70.0, 67.6, 67.3, 63.0, 54.8, 25.1, 25.0.

LRMS(ESI): 719.3 $[M+Na]^+$

HRMS(ESI-TOF) m/z: $[M+Na]^+$ Calcd for $C_{39}H_{36}NaO_{12}$ 719.2099; Found 719.2094.

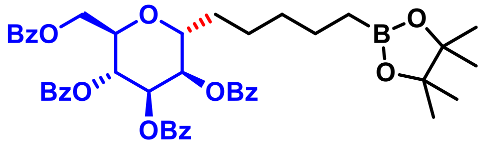

3as

(2*R*,3*R*,4*R*,5*R*,6*R*)-2-((benzoyloxy)methyl)-6-(5-(4,4,5,5-tetramethyl-1,3,2-dioxaborolan-2-yl)pentyl)tetrahydro-2*H*-pyran-3,4,5-triyl tribenzoate (3as)

Compound **3as** was obtained following the general procedure in 40% yield (61.0 mg) as colorless oil. (R_f= 0.3, PE/EA = 5 : 1 (v/v)).

^{1}H NMR (500 MHz, Chloroform-*d*) δ 8.11 (d, *J* = 7.2 Hz, 2H), 8.08 (d, *J* = 7.4 Hz, 2H), 8.00 (d, *J* = 7.3 Hz, 2H), 7.88 (d, *J* = 7.2 Hz, 2H), 7.64 – 7.51 (m, 3H), 7.50 – 7.37 (m, 7H), 7.31 (t, *J* = 7.8 Hz, 2H), 6.03 (t, *J* = 9.4 Hz, 1H), 5.83 (dd, *J* = 9.6, 3.3 Hz, 1H),

5.67 (t, J = 2.8 Hz, 1H), 4.65 (dd, J = 12.0, 2.8 Hz, 1H), 4.58 (dd, J = 12.0, 5.5 Hz, 1H), 4.35 – 4.25 (m, 2H), 2.14 – 2.02 (m, 1H), 1.85 – 1.75 (m, 1H), 1.50 – 1.39 (m, 5H), 1.28 (s, 12H), 1.25 – 1.22 (m, 1H), 0.81 (t, J = 7.2 Hz, 2H).

^{13}C NMR (125 MHz, Chloroform-*d*) δ 166.3, 165.7, 165.7, 165.5, 133.5, 133.3, 133.3, 133.0, 129.8, 129.8, 129.7, 129.0, 128.5, 128.5, 128.4, 128.4, 82.9, 75.9, 72.1, 70.3, 70.2, 69.9, 67.7, 63.4, 32.0, 28.4, 25.4, 24.8, 23.9.

LRMS(ESI): 799.4 [M+Na]$^+$

HRMS(ESI-TOF) m/z: [M+H]$^+$ Calcd for $C_{45}H_{50}$[11B]O_{11} 777.3441; Found 777.3439.

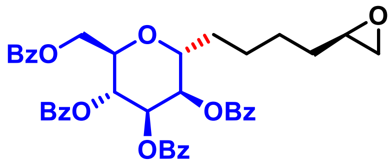

3at

(2*R*,3*R*,4*R*,5*R*,6*R*)-2-((benzoyloxy)methyl)-6-(4-((*R*)-oxiran-2-yl)butyl)tetrahydro-2*H*-pyran-3,4,5-triyl tribenzoate (3at)

Compound **3at** was obtained following the general procedure in 54% yield (71.8 mg) as colorless oil. (R_f = 0.6, PE/EA = 3 : 1 (v/v)).

^{1}H NMR (500 MHz, Acetone-d_6) δ 8.11 (dd, J = 8.3, 1.4 Hz, 2H), 8.05 (dd, J = 8.3, 1.4 Hz, 2H), 8.00 (dd, J = 8.4, 1.3 Hz, 2H), 7.83 (dd, J = 8.4, 1.4 Hz, 2H), 7.70 – 7.64 (m, 2H), 7.60 – 7.56 (m, 1H), 7.55 – 7.47 (m, 5H), 7.44 (t, J = 7.8 Hz, 2H), 7.35 (t, J = 7.8 Hz, 2H), 6.07 (t, J = 9.4 Hz, 1H), 5.91 (dd, J = 9.6, 3.3 Hz, 1H), 5.73 (t, J = 2.8 Hz, 1H), 4.73 – 4.60 (m, 2H), 4.56 (ddd, J = 8.1, 4.7, 2.9 Hz, 1H), 4.30 (ddd, J = 10.7, 4.5, 2.3 Hz, 1H), 2.86 – 2.81 (m, 1H), 2.63 (dd, J = 5.2, 3.9 Hz, 1H), 2.40 (dd, J = 5.2, 2.6 Hz, 1H), 2.31 – 2.19 (m, 1H), 1.94 – 1.83 (m, 1H), 1.77 – 1.67 (m, 1H), 1.66 – 1.46 (m, 5H).

^{13}C NMR (125 MHz, Acetone-d_6) δ 165.1, 164.7, 164.7, 164.7, 133.0, 133.0, 132.9, 132.7, 129.7, 129.5, 129.1, 129.0, 129.0, 128.9, 128.2, 128.1, 128.1, 128.0, 75.2, 71.7, 70.2, 69.6, 67.1, 62.6, 50.9, 45.5, 31.8, 27.5, 25.0, 24.8.

LRMS(ESI): 701.4 [M+Na]$^+$

HRMS(ESI-TOF) m/z: [M+H]$^+$ Calcd for $C_{40}H_{39}O_{10}$ 679.2538; Found 679.2542.

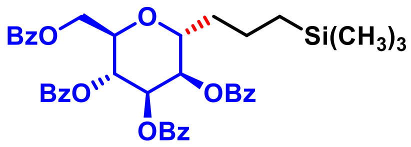

3au

(2*R*,3*R*,4*R*,5*R*,6*R*)-2-((benzoyloxy)methyl)-6-(3-(trimethylsilyl)propyl)tetrahydro-2*H*-pyran-3,4,5-triyl tribenzoate (3au)

Compound **3au** was obtained following the general procedure in 52% yield (70.8 mg) as colorless oil. (R_f = 0.6, PE/EA = 10 : 1 (v/v)).

^{1}H NMR (600 MHz, Chloroform-*d*) δ 8.09 (d, J = 7.9 Hz, 2H), 8.07 (d, J = 7.7 Hz, 2H), 7.98 (d, J = 7.8 Hz, 2H), 7.87 (d, J = 7.8 Hz, 2H), 7.62 – 7.54 (m, 2H), 7.52 (t, J = 7.2 Hz, 1H), 7.45 (d, J = 7.6 Hz, 1H), 7.44 – 7.36 (m, 6H), 7.30 (t, J = 7.7 Hz, 2H), 6.00 (t, J = 9.2 Hz, 1H), 5.82 (dd, J = 9.4, 3.1 Hz, 1H), 5.65 (t, J = 2.8 Hz, 1H), 4.67 – 4.58 (m, 2H), 4.36 – 4.29 (m, 2H), 2.18 – 2.07 (m, 1H), 1.81 – 1.73 (m, 1H), 1.64 – 1.56 (m, 1H), 1.52 – 1.40 (m, 1H), 0.64 (td, J = 13.7, 4.8 Hz, 1H), 0.55 (td, J = 14.0, 13.3, 4.8 Hz, 1H), -0.03 (s, 9H).

^{13}C NMR (150 MHz, Chloroform-*d*) δ 166.4, 165.9, 165.8, 165.6, 133.6, 133.5, 133.4, 133.2, 130.0, 129.9, 129.9, 129.8, 129.2, 128.7, 128.6, 128.6, 128.5, 75.5, 72.3, 70.5, 70.4, 67.9, 63.5, 32.6, 20.3, 16.5, -1.6.

LRMS(ESI): 701.4 $[M+NH_4]^+$

HRMS(ESI-TOF) m/z: $[M+H]^+$ Calcd for $C_{40}H_{43}O_9Si$ 695.2671; Found 695.2669.

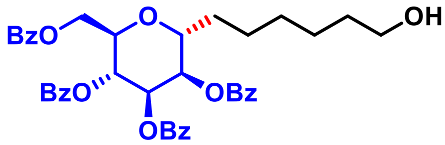

3av

(2*R*,3*R*,4*R*,5*R*,6*R*)-2-((benzoyloxy)methyl)-6-(6-hydroxyhexyl)tetrahydro-2*H*-pyran-3,4,5-triyl tribenzoate (3av)

Compound **3av** was obtained following the general procedure in 40% yield (53.3 mg) as colorless oil. (R_f = 0.3, PE/EA = 2 : 1 (v/v)).

^{1}H NMR (600 MHz, Chloroform-*d*) δ 8.09 (d, J = 7.9 Hz, 2H), 8.06 (d, J = 7.8 Hz, 2H), 7.98 (d, J = 7.9 Hz, 2H), 7.86 (d, J = 7.7 Hz, 2H), 7.60 – 7.55 (m, 2H), 7.52 (t, J = 7.4

Hz, 1H), 7.46 (t, J = 7.6 Hz, 1H), 7.42 (t, J = 7.7 Hz, 4H), 7.38 (t, J = 7.7 Hz, 2H), 7.30 (t, J = 7.7 Hz, 2H), 6.00 (t, J = 9.3 Hz, 1H), 5.81 (dd, J = 9.5, 3.1 Hz, 1H), 5.65 (s, 1H), 4.64 (dd, J = 12.0, 2.9 Hz, 1H), 4.59 (dd, J = 12.0, 5.5 Hz, 1H), 4.32 – 4.29 (m, 1H), 4.29 – 4.25 (m, 1H), 3.62 (t, J = 6.6 Hz, 2H), 2.09 – 2.01 (m, 1H), 1.84 – 1.75 (m, 1H), 1.58 – 1.35 (m, 8H).

^{13}C NMR (150 MHz, Chloroform-*d*) δ 166.2, 165.7, 165.7, 165.5, 133.5, 133.4, 133.3, 133.1, 129.9, 129.8, 129.8, 129.6, 129.0, 129.0, 128.5, 128.5, 128.4, 128.4, 75.7, 72.1, 70.3, 70.2, 67.8, 63.3, 62.9, 32.6, 28.9, 28.5, 25.6, 25.5.

LRMS(ESI): 698.1 $[M+NH_4]^+$

HRMS(ESI-TOF) m/z: $[M+H]^+$ Calcd for $C_{40}H_{41}O_{10}$ 681.2694; Found 681.2695.

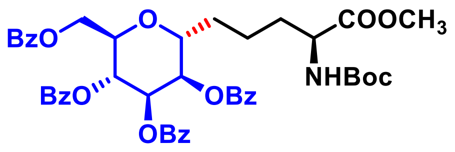

3aw

(2*R*,3*R*,4*R*,5*R*,6*R*)-2-((benzoyloxy)methyl)-6-((*S*)-4-((tert-butoxycarbonyl)amino)-5-methoxy-5-oxopentyl)tetrahydro-2*H*-pyran-3,4,5-triyl tribenzoate (3aw)

Compound **3aw** was obtained following the general procedure in 62% yield (98.7 mg) as colorless oil. (R_f = 0.3, PE/EA = 2 : 1 (v/v)).

^{1}H NMR (500 MHz, Chloroform-*d*) δ 8.08 (dd, J = 8.2, 1.4 Hz, 2H), 8.04 (dd, J = 8.2, 1.4 Hz, 2H), 7.98 (dd, J = 8.2, 1.4 Hz, 2H), 7.86 (dd, J = 8.2, 1.4 Hz, 2H), 7.58 (q, J = 7.4 Hz, 2H), 7.52 (t, J = 7.4 Hz, 1H), 7.48 – 7.36 (m, 7H), 7.30 (t, J = 7.8 Hz, 2H), 6.02 (t, J = 9.0 Hz, 1H), 5.79 (dd, J = 9.3, 3.3 Hz, 1H), 5.63 (t, J = 3.0 Hz, 1H), 4.61 (d, J = 4.1 Hz, 2H), 4.38 – 4.19 (m, 3H), 3.72 (s, 3H), 2.11 – 2.03 (m, 1H), 1.98 – 1.89 (m, 1H), 1.87 – 1.81 (m, 1H), 1.78 – 1.70 (m, 1H), 1.68 – 1.62 (m, 1H), 1.61 – 1.53 (m, 1H), 1.44 (s, 9H).

^{13}C NMR (125 MHz, Chloroform-*d*) δ 173.1, 166.3, 165.7, 165.6, 165.4, 155.4, 133.5, 133.4, 133.3, 133.1, 129.8, 129.8, 129.8, 129.6, 129.0, 129.0, 128.6, 128.5, 128.4, 80.0, 75.3, 71.8, 70.5, 70.1, 67.7, 63.1, 53.2, 52.3, 32.4, 28.3, 28.1, 21.6.

LRMS(ESI): 832.3 $[M+Na]^+$

HRMS(ESI-TOF) m/z: $[M+Na]^+$ Calcd for $C_{45}H_{47}NNaO_{13}$ 832.294; Found 832.2942.

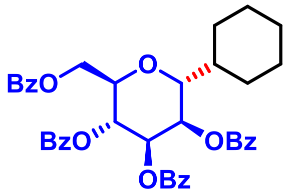

3ax

(2*R*,3*R*,4*R*,5*R*,6*R*)-2-((benzoyloxy)methyl)-6-cyclohexyltetrahydro-2*H*-pyran-3,4,5-triyl tribenzoate (3ax)

Compound **3ax** was obtained following the general procedure in 32% yield (41.5 mg) as colorless oil. (R_f = 0.6, PE/EA = 6 : 1 (v/v)).

^{1}H NMR (500 MHz, Chloroform-*d*) δ 8.11 – 8.03 (m, 4H), 7.97 (dd, *J* = 8.3, 1.3 Hz, 2H), 7.86 (dd, *J* = 8.3, 1.3 Hz, 2H), 7.61 – 7.54 (m, 2H), 7.54 – 7.49 (m, 1H), 7.47 – 7.35 (m, 7H), 7.29 (t, *J* = 7.8 Hz, 2H), 6.07 (t, *J* = 9.7 Hz, 1H), 5.93 (dd, *J* = 3.2, 2.0 Hz, 1H), 5.74 (dd, *J* = 9.8, 3.1 Hz, 1H), 4.63 (dd, *J* = 12.0, 2.7 Hz, 1H), 4.53 (dd, *J* = 12.0, 5.3 Hz, 1H), 4.25 (ddd, *J* = 9.7, 5.4, 2.7 Hz, 1H), 3.89 (dd, *J* = 10.6, 2.0 Hz, 1H), 2.16 – 1.99 (z), 1.87 (dd, *J* = 13.3, 4.4 Hz, 1H), 1.80 (d, *J* = 13.2 Hz, 1H), 1.77 – 1.69 (m, 1H), 1.49 – 1.36 (m, 1H), 1.37 – 1.15 (m, 3H), 1.14 – 1.02 (m, 1H).

^{13}C NMR (500 MHz, Chloroform-*d*) δ 165.8, 165.4, 165.3, 165.0, 133.0, 132.8, 132.6, 129.4, 129.4, 129.3, 128.6, 128.1, 128.0, 127.9, 127.9, 80.4, 70.1, 70.0, 69.7, 67.0, 63.1, 35.0, 28.8, 28.6, 25.6, 25.2, 25.0.

LRMS(ESI): 680.0 $[M+NH_4]^+$, 685.3 $[M+Na]^+$

HRMS(ESI-TOF) m/z: $[M+H]^+$ Calcd for $C_{40}H_{39}O_9$ 663.2589; Found 663.2590.

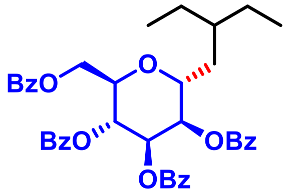

3ay

(2*R*,3*R*,4*R*,5*R*,6*R*)-2-((benzoyloxy)methyl)-6-(2-ethylbutyl)tetrahydro-2*H*-pyran-3,4,5-triyl tribenzoate (3ay)

Compound **3ay** was not obtained following the general procedure.

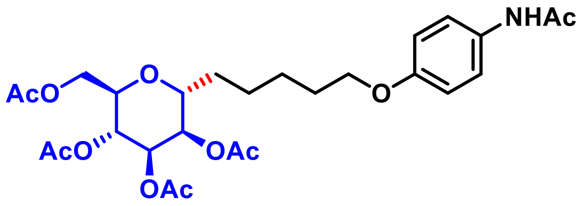

3bn

(2*R*,3*R*,4*R*,5*R*,6*R*)-2-(5-(4-acetamidophenoxy)pentyl)-6-(acetoxymethyl)tetrahydro-2*H*-pyran-3,4,5-triyl triacetate (3bn)

Compound **3bn** was obtained following the general procedure in 54% yield (59.6 mg) as colorless oil. (R_f= 0.3, PE/EA = 1 : 1 (v/v)).

^{1}H NMR (600 MHz, Chloroform-*d*) δ 7.39 (d, *J* = 8.9 Hz, 2H), 7.29 (s, 1H), 6.85 (d, *J* = 8.9 Hz, 2H), 5.26 (dd, *J* = 9.1, 3.2 Hz, 1H), 5.22 (t, *J* = 8.6 Hz, 1H), 5.18 (t, *J* = 3.0 Hz, 1H), 4.33 (dd, *J* = 12.1, 6.1 Hz, 1H), 4.11 (dd, *J* = 12.0, 2.8 Hz, 1H), 4.00 – 3.96 (m, 1H), 3.95 (t, *J* = 6.4 Hz, 2H), 3.87 (td, *J* = 6.0, 3.1 Hz, 1H), 2.16 (s, 3H), 2.15 (s, 3H), 2.10 (s, 3H), 2.07 (s, 3H), 2.04 (s, 3H), 1.86 – 1.77 (m, 3H), 1.65 – 1.58 (m, 1H), 1.56 – 1.48 (m, 3H), 1.47 – 1.40 (m, 1H).

^{13}C NMR (125 MHz, Chloroform-*d*) δ 170.3, 169.9, 169.6, 169.3, 167.8, 155.4, 130.5, 121.4, 114.3, 74.6, 70.4, 69.7, 68.6, 67.5, 66.5, 62.2, 28.7, 28.0, 25.2, 24.7, 23.9, 20.5, 20.3, 20.3.

LRMS(ESI): 569.0 $[M+NH_4]^+$

HRMS(ESI-TOF) m/z: $[M+H]^+$ Calcd for $C_{27}H_{38}NO_{11}$ 552.2442; Found 552.2439.

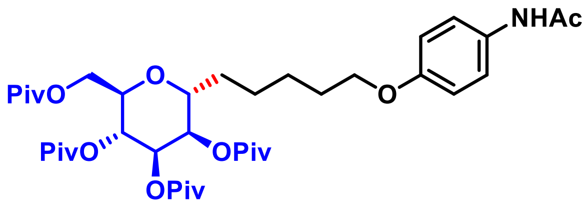

3cn

(2*R*,3*R*,4*R*,5*R*,6*R*)-2-(5-(4-acetamidophenoxy)pentyl)-6-((pivaloyloxy)methyl)tetrahydro-2*H*-pyran-3,4,5-triyl tris(2,2-dimethylpropanoate) (3cn)

Compound **3cn** was obtained following the general procedure in 62% yield (67.7 mg) as colorless oil. (R_f= 0.3, PE/EA = 2 : 1 (v/v)).

^{1}H NMR (500 MHz, Chloroform-*d*) δ 7.30 (d, *J* = 9.0 Hz, 2H), 7.10 (s, 1H), 6.77 (d, *J*

= 9.0 Hz, 2H), 5.32 (t, *J* = 9.3 Hz, 1H), 5.20 (dd, *J* = 9.5, 3.2 Hz, 1H), 5.10 (t, *J* = 2.8 Hz, 1H), 4.17 (dd, *J* = 12.2, 5.4 Hz, 1H), 4.02 (dd, *J* = 12.3, 2.0 Hz, 1H), 3.89 – 3.83 (m, 3H), 3.82 – 3.77 (m, 1H), 2.08 (s, 3H), 1.81 – 1.68 (m, 3H), 1.53 – 1.32 (m, 5H), 1.19 (s, 9H), 1.17 (s, 9H), 1.11 (s, 9H), 1.07 (s, 9H).

^{13}C NMR (125 MHz, Chloroform-*d*) δ 178.2, 177.5, 177.5, 176.8, 168.2, 155.9, 130.8, 121.9, 114.8, 75.7, 71.0, 70.5, 69.5, 68.0, 66.0, 62.4, 38.9, 38.9, 38.8, 29.1, 28.4, 27.2, 27.2, 27.1, 27.1, 25.8, 25.4, 24.4.

LRMS(ESI): 737.1 $[M+NH_4]^+$, 720.4 $[M+H]^+$

HRMS(ESI-TOF) m/z: $[M+H]^+$ Calcd for $C_{39}H_{62}NO_{11}$ 720.4318; Found 720.4317.

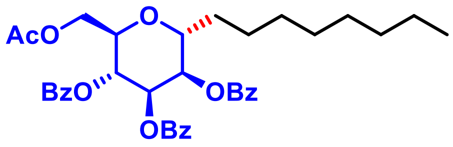

3da

(2*R*,3*R*,4*R*,5*R*,6*R*)-2-(acetoxymethyl)-6-octyltetrahydro-2*H*-pyran-3,4,5-triyl tribenzoate (3da)

Compound **3da** was obtained following the general procedure in 68% yield (85.8 mg) as colorless oil. (R_f = 0.5, PE/EA = 6 : 1 (v/v)).

^{1}H NMR (500 MHz, Chloroform-*d*) δ 8.11 (d, *J* = 7.1 Hz, 2H), 8.00 (d, *J* = 7.1 Hz, 2H), 7.88 (d, *J* = 7.0 Hz, 2H), 7.62 (t, *J* = 7.4 Hz, 1H), 7.55 (t, *J* = 7.4 Hz, 1H), 7.53 – 7.44 (m, 3H), 7.42 (t, *J* = 7.7 Hz, 2H), 7.31 (t, *J* = 7.7 Hz, 2H), 5.89 (t, *J* = 9.3 Hz, 1H), 5.80 (dd, *J* = 9.5, 3.3 Hz, 1H), 5.66 (t, *J* = 2.9 Hz, 1H), 4.45 (dd, *J* = 12.0, 5.8 Hz, 1H), 4.30 (dd, *J* = 12.0, 3.0 Hz, 1H), 4.29 – 4.24 (m, 1H), 4.22 – 4.16 (m, 1H), 2.11 (s, 3H), 2.09 – 1.97 (m, 1H), 1.85 – 1.75 (m, 1H), 1.64 – 1.53 (m, 1H), 1.52 – 1.40 (m, 3H), 1.39 – 1.29 (m, 8H), 0.92 (t, *J* = 6.6 Hz, 3H).

^{13}C NMR (125 MHz, Chloroform-*d*) δ 172.1, 167.1, 167.1, 166.9, 134.9, 134.8, 134.7, 131.3, 131.2, 131.2, 131.1, 130.4, 130.4, 130.0, 129.9, 129.8, 77.3, 73.4, 71.6, 71.5, 69.1, 64.5, 33.3, 30.9, 30.6, 30.0, 27.0, 24.1, 22.1, 15.5.

LRMS(ESI): 653.4 $[M+Na]^+$

HRMS(ESI-TOF) m/z: $[M+H]^+$ Calcd for $C_{37}H_{43}O_9$ 631.2904; Found 631.2904.

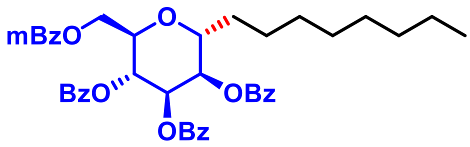

3ea

(2*R*,3*R*,4*R*,5*R*,6*R*)-2-(((4-methoxybenzoyl)oxy)methyl)-6-octyltetrahydro-2*H*-pyran-3,4,5-triyl tribenzoate (3ea)

Compound **3ea** was obtained following the general procedure in 70% yield (101.2 mg) as colorless oil. (R_f = 0.4, PE/EA = 6 : 1 (v/v)).

^{1}H NMR (500 MHz, Chloroform-*d*) δ 8.09 (d, *J* = 8.0 Hz, 2H), 8.06 (d, *J* = 8.7 Hz, 2H), 8.00 (d, *J* = 7.8 Hz, 2H), 7.89 (d, *J* = 7.8 Hz, 2H), 7.61 (t, *J* = 7.4 Hz, 1H), 7.54 (t, *J* = 7.4 Hz, 1H), 7.50 – 7.43 (m, 3H), 7.40 (t, *J* = 7.7 Hz, 2H), 7.32 (t, *J* = 7.6 Hz, 2H), 6.90 (d, *J* = 8.4 Hz, 2H), 6.01 (t, *J* = 9.3 Hz, 1H), 5.83 (dd, *J* = 9.5, 3.3 Hz, 1H), 5.67 (d, *J* = 2.7 Hz, 1H), 4.64 (dd, *J* = 12.0, 2.9 Hz, 1H), 4.56 (dd, *J* = 12.0, 5.7 Hz, 1H), 4.35 – 4.26 (m, 2H), 3.89 (s, 3H), 2.13 – 2.01 (m, 1H), 1.85 – 1.75 (m, 1H), 1.61 – 1.54 (m, 1H), 1.52 – 1.26 (m, 11H), 0.91 (t, *J* = 6.7 Hz, 3H).

^{13}C NMR (125 MHz, Chloroform-*d*) δ 166.0, 165.7, 165.5, 163.4, 133.4, 133.3, 131.8, 129.8, 129.7, 129.7, 129.1, 129.0, 128.5, 128.5, 128.4, 113.6, 75.8, 72.2, 70.3, 67.8, 63.1, 55.4, 31.9, 30.9, 29.5, 29.3, 29.2, 29.1, 28.6, 28.5, 25.6, 22.6, 14.1.

LRMS(ESI): 745.3 $[M+Na]^+$

HRMS(ESI-TOF) m/z: $[M+H]^+$ Calcd for $C_{43}H_{47}O_{10}$ 723.3164; Found 723.3168.

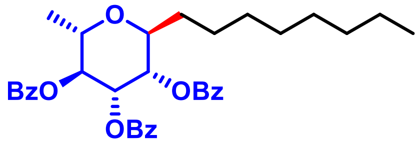

3fa

(2*S*,3*S*,4*R*,5*S*,6*S*)-2-methyl-6-octyltetrahydro-2*H*-pyran-3,4,5-triyl tribenzoate (3fa)

Compound **3fa** was obtained following the general procedure in 65% yield (74.5 mg) as colorless oil. (R_f = 0.5, PE/EA = 8 : 1 (v/v)).

^{1}H NMR (600 MHz, Chloroform-*d*) δ 8.11 (d, *J* = 7.1 Hz, 2H), 8.00 (d, *J* = 7.3 Hz, 2H), 7.86 (d, *J* = 7.3 Hz, 2H), 7.62 (t, *J* = 7.5 Hz, 1H), 7.55 (t, *J* = 7.4 Hz, 1H), 7.53 – 7.44

(m, 3H), 7.42 (t, J = 7.8 Hz, 2H), 7.30 (t, J = 7.8 Hz, 2H), 5.75 (dd, J = 9.7, 3.4 Hz, 1H), 5.66 (t, J = 9.3 Hz, 1H), 5.64 (dd, J = 3.4, 2.1 Hz, 1H), 4.18 (ddd, J = 10.0, 5.1, 2.1 Hz, 1H), 4.09 – 4.01 (m, 1H), 2.08 – 1.99 (m, 1H), 1.83 – 1.73 (m, 1H), 1.58 – 1.53 (m, 1H), 1.52 – 1.29 (m, 14H), 0.92 (t, J = 6.8 Hz, 3H).

^{13}C NMR (150 MHz, Chloroform-*d*) δ 165.9, 165.8, 165.8, 133.3, 133.3, 133.2, 129.9, 129.7, 129.7, 129.4, 129.2, 128.5, 128.5, 128.3, 75.9, 72.5, 72.4, 70.3, 68.0, 31.9, 29.5, 29.3, 29.3, 28.7, 25.8, 22.7, 18.1, 14.1.

LRMS(ESI): 595.4 $[M+Na]^+$

HRMS(ESI-TOF) m/z: $[M+H]^+$ Calcd for $C_{35}H_{41}O_7$ 573.2847; Found 573.2845.

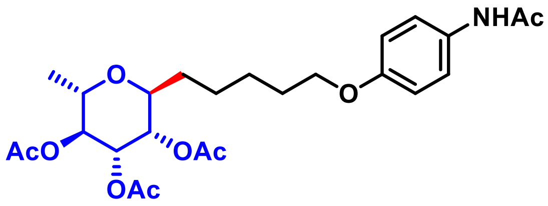

3gn

(2*S*,3*S*,4*R*,5*S*,6*S*)-2-(5-(4-acetamidophenoxy)pentyl)-6-methyltetrahydro-2*H*-pyran-3,4,5-triyl triacetate (3gn)

Compound **3gn** was obtained following the general procedure in 49% yield (48.4 mg) as colorless oil. (R_f = 0.3, PE/EA = 2 : 1 (v/v)).

^{1}H NMR (600 MHz, Chloroform-*d*) δ 7.39 (d, J = 8.9 Hz, 2H), 7.22 (s, 1H), 6.86 (d, J = 8.9 Hz, 2H), 5.22 – 5.18 (m, 2H), 5.06 (t, J = 8.8 Hz, 1H), 3.95 (t, J = 6.4 Hz, 2H), 3.89 (ddd, J = 10.2, 4.9, 2.1 Hz, 1H), 3.74 (dt, J = 8.7, 6.3 Hz, 1H), 2.16 (s, 3H), 2.15 (s, 3H), 2.08 (s, 3H), 2.03 (s, 3H), 1.89 – 1.77 (m, 3H), 1.62 – 1.42 (m, 5H), 1.25 (d, J = 6.2 Hz, 3H).

^{13}C NMR (125 MHz, Chloroform-*d*) δ 170.0, 169.8, 169.5, 167.7, 155.5, 130.4, 121.4, 114.3, 74.8, 71.1, 70.8, 68.9, 67.5, 67.3, 28.7, 28.1, 25.2, 25.0, 23.9, 20.6, 20.4, 20.3, 17.3.

LRMS(ESI): 494.3 $[M+H]^+$, 511.1 $[M+NH_4]^+$

HRMS(ESI-TOF) m/z: $[M+H]^+$ Calcd for $C_{25}H_{36}NO_9$ 494.2385; Found 494.2384.

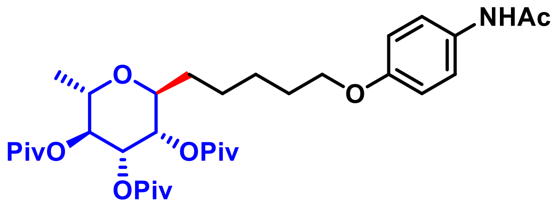

3hn

(2*S*,3*S*,4*R*,5*S*,6*S*)-2-(5-(4-acetamidophenoxy)pentyl)-6-methyltetrahydro-2*H*-pyran-3,4,5-triyl tris(2,2-dimethylpropanoate) (3hn)

Compound **3hn** was obtained following the general procedure in 62% yield (52.1 mg) as colorless oil. (R_f= 0.3, PE/EA = 2 : 1 (v/v)).

^{1}H NMR (500 MHz, Chloroform-*d*) δ 7.37 (d, *J* = 8.9 Hz, 2H), 7.12 (s, 1H), 6.84 (d, *J* = 9.0 Hz, 2H), 5.22 (dd, *J* = 9.7, 3.2 Hz, 1H), 5.15 (dd, *J* = 3.2, 2.2 Hz, 1H), 5.11 (t, *J* = 9.3 Hz, 1H), 3.93 (t, *J* = 5.9, 2H), 3.84 (ddd, *J* = 10.3, 4.8, 2.2 Hz, 1H), 3.77 – 3.70 (m, 1H), 2.15 (s, 3H), 1.91 – 1.84 (m, 1H), 1.82 – 1.75 (m, 2H), 1.55 – 1.38 (m, 5H), 1.25 (s, 9H), 1.20 (d, *J* = 6.2 Hz, 3H), 1.18 (s, 9H), 1.13 (s, 9H).

^{13}C NMR (125 MHz, Chloroform-*d*) δ 177.6, 177.5, 177.2, 168.1, 156.0, 130.8, 121.9, 114.8, 75.8, 71.3, 71.2, 69.5, 68.0, 67.8, 38.9, 38.8, 38.8, 29.2, 28.4, 25.8, 25.5, 24.4, 17.9.

LRMS(ESI): 642.5 $[M+Na]^+$, 637.3 $[M+NH_4]^+$

HRMS(ESI-TOF) m/z: $[M+H]^+$ Calcd for $C_{34}H_{54}NO_9$ 620.3793; Found 620.3798.

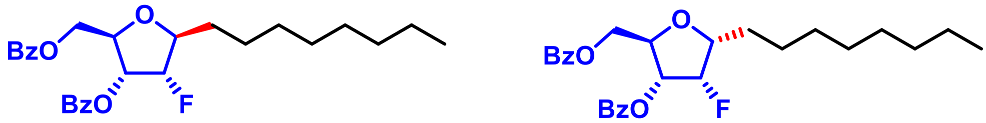

3ia (1 : 1)

((2*R*,3*R*,4*S*,5*S*)-3-(benzoyloxy)-4-fluoro-5-octyltetrahydrofuran-2-yl)methyl benzoate (3ia)

Compound **3ia** was obtained following the general procedure in 56% yield (51.1 mg) as colorless oil. (R_f= 0.4, PE/EA = 4 : 1 (v/v)).

^{1}H NMR (500 MHz, Chloroform-*d*) δ 8.09 (dd, *J* = 8.3, 1.4 Hz, 2H), 8.03 (dd, *J* = 8.4, 1.4 Hz, 2H), 7.65 – 7.59 (m, 1H), 7.58 – 7.53 (m, 1H), 7.50 – 7.38 (m, 4H), 5.62 (dd, *J* = 3.4, 1.8 Hz, 0.5H), 5.58 (dd, *J* = 3.5, 1.8 Hz, 0.5H), 5.06 (dd, *J* = 3.1, 1.8 Hz, 0.5H), 4.96 (dd, *J* = 3.1, 1.8 Hz, 0.5H), 4.65 (dd, *J* = 11.7, 5.6 Hz, 1H), 4.58 (dd, *J* = 11.7, 4.5

Hz, 1H), 4.45 (ddd, *J* = 5.6, 4.4, 3.2 Hz, 1H), 4.36 (ddd, *J* = 8.6, 6.2, 3.0 Hz, 0.5H), 4.32 (ddd, *J* = 8.8, 6.2, 3.0 Hz, 0.5H), 1.79 – 1.70 (m, 1H), 1.69 – 1.60 (m, 1H), 1.54 – 1.45 (m, 1H), 1.44 – 1.38 (m, 1H), 1.37 – 1.17 (m, 10H), 0.87 (t, *J* = 7.0 Hz, 3H).

^{13}C NMR (125 MHz, Chloroform-*d*) δ 165.9, 165.1, 133.2, 132.6, 129.4, 129.3, 128.1, 127.9, 99.5, 98.0, 82.7, 82.5, 80.2, 80.2, 79.1, 78.9, 63.7, 31.4, 31.3, 29.0, 28.9, 28.7, 25.0, 22.2, 13.6.

^{19}F NMR (471 MHz, Chloroform-*d*) δ -184.29 (t, *J* = 20.1 Hz), -184.40 (t, *J* = 20.1 Hz)

LRMS(ESI): 479.4 [M+Na]$^+$,

HRMS(ESI-TOF) m/z: [M+H]$^+$ Calcd for $C_{27}H_{34}FO_5$ 457.2385; Found 457.2389.

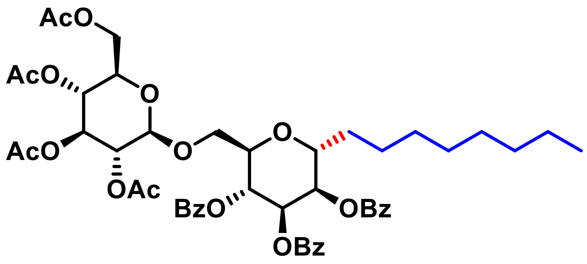

3ja

(2*R*,3*R*,4*R*,5*R*,6*R*)-2-octyl-6-((((2*R*,3*R*,4*S*,5*R*,6*R*)-3,4,5-triacetoxy-6-(acetoxymethyl)tetrahydro-2*H*-pyran-2-yl)oxy)methyl)tetrahydro-2*H*-pyran-3,4,5-triyl tribenzoate (3ja)

Compound **3ja** was obtained following the general procedure in 51% yield (93.7 mg) as colorless oil. (R_f = 0.3, PE/EA = 2 : 1 (v/v)).

^{1}H NMR (500 MHz, Chloroform-*d*) δ 8.09 (dd, *J* = 8.3, 1.4 Hz, 2H), 7.95 (dd, *J* = 8.3, 1.4 Hz, 2H), 7.80 (dd, *J* = 8.3, 1.4 Hz, 2H), 7.63 – 7.58 (m, 1H), 7.56 – 7.48 (m, 3H), 7.46 – 7.36 (m, 3H), 7.29 – 7.24 (m, 2H), 5.78 – 5.70 (m, 2H), 5.62 (d, *J* = 2.6 Hz, 1H), 5.19 (t, *J* = 9.5 Hz, 1H), 5.09 – 5.00 (m, 2H), 4.58 (d, *J* = 7.9 Hz, 1H), 4.25 – 4.16 (m, 2H), 4.15 – 4.02 (m, 3H), 3.74 (dd, *J* = 10.8, 6.4 Hz, 1H), 3.69 – 3.62 (m, 1H), 2.08 (s, 3H), 2.01 – 1.94 (m, 10H), 1.83 – 1.72 (m, 1H), 1.59 – 1.55 (m, 1H), 1.46 – 1.27 (m, 11H), 0.89 (t, *J* = 7.0 Hz, 3H).

^{13}C NMR (125 MHz, Chloroform-*d*) δ 169.6, 169.3, 168.4, 168.3, 164.9, 164.7, 164.4, 132.5, 132.4, 132.2, 128.9, 128.7, 128.7, 128.6, 128.0, 127.6, 127.5, 127.3, 100.0, 75.2,

71.8, 71.1, 70.8, 70.2, 70.0, 69.4, 67.8, 67.4, 66.6, 60.9, 30.8, 28.3, 27.6, 24.5, 21.7, 19.7, 19.6, 19.6, 13.1.

LRMS(ESI): 936.1 [M+NH$_4$]$^+$, 941.4 [M+Na]$^+$

HRMS(ESI-TOF) m/z: [M+Na]$^+$ Calcd for $C_{49}H_{58}NaO_{17}$ 941.3566; Found 941.3565.

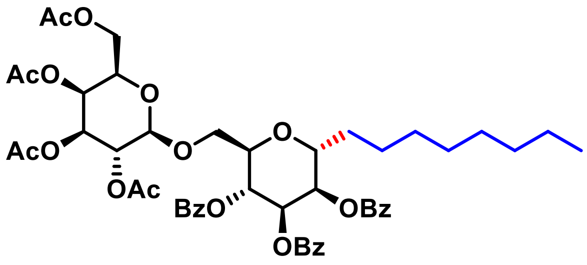

3ka

(2*R*,3*R*,4*R*,5*R*,6*R*)-2-octyl-6-((((2*R*,3*R*,4*S*,5*S*,6*R*)-3,4,5-triacetoxy-6-(acetoxymethyl)tetrahydro-2*H*-pyran-2-yl)oxy)methyl)tetrahydro-2*H*-pyran-3,4,5-triyl tribenzoate (3ka)

Compound **3ka** was obtained following the general procedure in 49% yield (90.0 mg) as colorless oil. (R_f = 0.3, PE/EA = 2 : 1 (v/v)).

^{1}H NMR (500 MHz, Chloroform-*d*) δ 8.12 – 8.08 (m, 2H), 7.97 – 7.93 (m, 2H), 7.83 – 7.78 (m, 2H), 7.63 – 7.57 (m, 1H), 7.55 – 7.46 (m, 3H), 7.46 – 7.36 (m, 3H), 7.29 – 7.24 (m, 2H), 5.78 – 5.70 (m, 2H), 5.63 (t, *J* = 2.2 Hz, 1H), 5.36 (d, *J* = 2.9 Hz, 1H), 5.28 (dd, *J* = 10.4, 7.9 Hz, 1H), 4.99 (dd, *J* = 10.5, 3.4 Hz, 1H), 4.53 (d, *J* = 8.0 Hz, 1H), 4.19 (ddd, *J* = 9.9, 5.4, 1.9 Hz, 1H), 4.15 – 4.01 (m, 4H), 3.87 (t, *J* = 6.8 Hz, 1H), 3.72 (dd, *J* = 10.9, 6.5 Hz, 1H), 2.10 (s, 3H), 2.09 (s, 3H), 1.98 (s, 3H), 1.97 (s, 3H), 1.82 – 1.72 (m, 1H), 1.62 – 1.54 (m, 2H), 1.46 – 1.27 (m, 11H), 0.89 (t, *J* = 7.0 Hz, 3H).

^{13}C NMR (125 MHz, Chloroform-*d*) δ 169.3, 169.2, 169.2, 168.4, 164.9, 164.7, 164.5, 132.5, 132.3, 132.2, 128.9, 128.7, 128.7, 128.6, 128.0, 128.0, 127.6, 127.5, 127.3, 100.5, 75.2, 71.1, 70.2, 70.0, 69.6, 69.4, 67.9, 67.5, 66.6, 66.0, 60.1, 30.9, 28.3, 27.6, 24.5, 21.7, 19.8, 19.6, 19.6, 19.6, 13.1.

LRMS(ESI): 936.1 [M+NH$_4$]$^+$, 941.4 [M+Na]$^+$

HRMS(ESI-TOF) m/z: [M+Na]$^+$ Calcd for $C_{49}H_{58}NaO_{17}$ 941.3566; Found 941.3567.

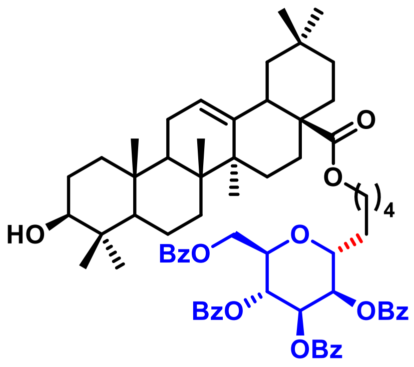

5aa

(2*R*,3*R*,4*R*,5*R*,6*R*)-2-((benzoyloxy)methyl)-6-(5-(((4a*S*,6a*S*,6b*R*,10*S*,12a*R*)-10-hydroxy-2,2,6a,6b,9,9,12a-heptamethyl-1,2,3,4,4a,5,6,6a,6b,7,8,8a,9,10,11,12,12a,12b,13,14b-icosahydropicene-4a-carbonyl)oxy)pentyl)tetrahydro-2*H*-pyran-3,4,5-triyl tribenzoate (5aa)

Compound **5aa** was obtained following the general procedure in 48% yield (106.0 mg) as colorless oil. (R_f = 0.2, PE/EA = 4 : 1 (v/v)).

^{1}H NMR (600 MHz, Chloroform-*d*) δ 8.11 (d, *J* = 7.2 Hz, 2H), 8.08 (d, *J* = 7.2 Hz, 2H), 8.00 (d, *J* = 7.2 Hz, 2H), 7.88 (d, *J* = 7.3 Hz, 2H), 7.64 – 7.56 (m, 2H), 7.55 (t, *J* = 7.5 Hz, 1H), 7.48 (t, *J* = 7.4 Hz, 1H), 7.47 – 7.38 (m, 6H), 7.32 (t, *J* = 7.7 Hz, 2H), 6.03 (t, *J* = 9.3 Hz, 1H), 5.83 (dd, *J* = 9.5, 3.3 Hz, 1H), 5.68 (t, *J* = 2.9 Hz, 1H), 5.31 (t, *J* = 3.7 Hz, 1H), 4.66 (dd, *J* = 12.0, 2.9 Hz, 1H), 4.61 (dd, *J* = 12.0, 5.5 Hz, 1H), 4.35 – 4.26 (m, 2H), 4.03 (td, *J* = 6.4, 2.0 Hz, 2H), 3.21 (dd, *J* = 11.5, 4.3 Hz, 1H), 2.89 (dd, *J* = 13.9, 4.6 Hz, 1H), 2.14 – 2.07 (m, 1H), 2.02 – 1.94 (m, 1H), 1.92 – 1.87 (m, 2H), 1.84 – 1.79 (m, 1H), 1.75 – 1.33 (m, 24H), 1.23 – 1.18 (m, 1H), 1.15 (s, 3H), 1.11 – 1.06 (m, 1H), 0.98 (s, 3H), 0.95 (s, 3H), 0.91 (s, 3H), 0.90 (s, 3H), 0.76 (s, 3H), 0.75 (s, 3H).

^{13}C NMR (150 MHz, Chloroform-*d*) δ 177.8, 166.2, 165.7, 165.7, 165.5, 143.9, 133.5, 133.4, 133.4, 133.1, 129.8, 129.8, 129.6, 129.0, 129.0, 128.6, 128.5, 128.4, 128.4, 122.4, 79.0, 75.6, 72.0, 70.3, 70.2, 67.7, 64.0, 63.3, 60.4, 55.2, 47.6, 46.7, 45.9, 41.7, 41.3, 39.3, 38.7, 38.4, 37.0, 33.9, 33.1, 32.8, 32.5, 30.7, 28.6, 28.6, 28.1, 27.7, 27.2, 25.9, 25.8, 25.3, 23.7, 23.4, 23.0, 21.1, 19.2, 18.3, 17.1, 15.6, 15.3, 14.2.

LRMS(ESI): 1127.6 [M+Na]$^+$

HRMS(ESI-TOF) m/z: [M+Na]$^+$ Calcd for $C_{69}H_{84}NaO_{12}$ 1127.5855; Found 1127.5859.

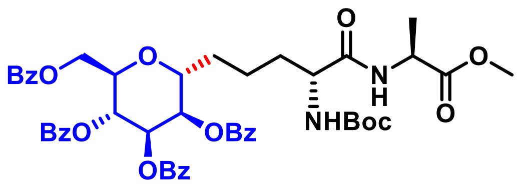

6aa

(2*R*,3*R*,4*R*,5*R*,6*R*)-2-((benzoyloxy)methyl)-6-((*R*)-4-((tert-butoxycarbonyl)amino)-5-(((*S*)-1-methoxy-1-oxopropan-2-yl)amino)-5-oxopentyl)tetrahydro-2*H*-pyran-3,4,5-triyl tribenzoate (6aa)

Compound **6aa** was obtained following the general procedure in 55% yield (96.8 mg) as colorless oil. (R_f = 0.4, PE/EA = 2 : 1 (v/v)).

^{1}H NMR (500 MHz, Chloroform-*d*) δ 8.11 (d, *J* = 7.2 Hz, 2H), 8.06 (d, *J* = 6.8 Hz, 2H), 8.00 (d, *J* = 6.9 Hz, 2H), 7.88 (d, *J* = 6.9 Hz, 2H), 7.64 – 7.56 (m, 2H), 7.58 – 7.50 (m, 1H), 7.51 – 7.37 (m, 7H), 7.32 (t, *J* = 7.8 Hz, 2H), 6.77 (s, 1H), 6.07 (t, *J* = 9.2 Hz, 1H), 5.82 (dd, *J* = 9.5, 3.3 Hz, 1H), 5.67 (t, *J* = 2.9 Hz, 1H), 5.11 (s, 1H), 4.65 (dd, *J* = 12.1, 3.0 Hz, 1H), 4.62 – 4.54 (m, 2H), 4.37 – 4.26 (m, 2H), 4.24 – 4.17 (m, 1H), 3.76 (s, 3H), 2.22 – 2.13 (m, 1H), 2.01 – 1.94 (m, 1H), 1.82 – 1.66 (m, 4H), 1.47 (s, 9H), 1.43 (d, *J* = 7.2 Hz, 3H).

^{13}C NMR (125 MHz, Chloroform-*d*) δ 173.1, 171.4, 166.2, 165.7, 165.6, 165.4, 133.4, 133.3, 133.3, 133.0, 129.8, 129.7, 129.7, 129.5, 128.9, 128.9, 128.5, 128.4, 128.4, 128.3, 71.8, 70.3, 70.2, 67.4, 63.0, 52.4, 48.0, 31.9, 28.2, 21.6, 18.2.

LRMS(ESI): 881.1 $[M+H]^+$

HRMS(ESI-TOF) m/z: $[M+H]^+$ Calcd for $C_{48}H_{53}N_2O_{14}$ 881.3491; Found 881.3492.

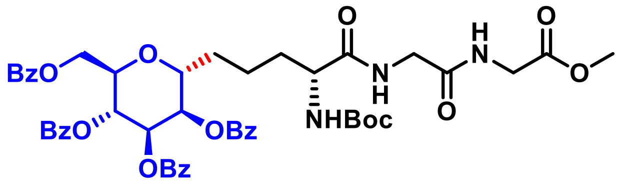

7aa

(2*R*,3*R*,4*R*,5*R*,6*R*)-2-((benzoyloxy)methyl)-6-((*R*)-4-((tert-butoxycarbonyl)amino)-5-((2-((2-methoxy-2-oxoethyl)amino)-2-oxoethyl)amino)-5-oxopentyl)tetrahydro-2*H*-pyran-3,4,5-triyl tribenzoate (7aa)

Compound **7aa** was obtained following the general procedure in 52% yield (96.0 mg) as colorless oil. (R_f = 0.4, PE/EA = 1 : 1 (v/v)).

^{1}H NMR (500 MHz, Chloroform-*d*) δ 8.08 (d, *J* = 7.1 Hz, 2H), 8.02 (d, *J* = 7.0 Hz, 2H), 7.97 (d, *J* = 7.3 Hz, 2H), 7.86 (d, *J* = 7.2 Hz, 2H), 7.61 – 7.55 (m, 2H), 7.54 – 7.49 (m, 1H), 7.48 – 7.35 (m, 7H), 7.30 (t, *J* = 7.7 Hz, 2H), 7.09 (s, 1H), 7.00 (t, *J* = 6.0 Hz, 1H), 6.02 (t, *J* = 9.1 Hz, 1H), 5.79 (dd, *J* = 9.4, 3.3 Hz, 1H), 5.64 (t, *J* = 2.9 Hz, 1H), 5.24 (d, *J* = 6.9 Hz, 1H), 4.71 – 4.55 (m, 2H), 4.35 – 4.22 (m, 2H), 4.12 – 4.00 (m, 3H), 4.00 – 3.90 (m, 2H), 3.68 (s, 3H), 2.16 – 2.05 (m, 1H), 1.98 – 1.90 (m, 2H), 1.87 – 1.80 (m, 1H), 1.73 – 1.65 (m, 1H), 1.61 – 1.52 (m, 1H), 1.42 (s, 9H).

^{13}C NMR (125 MHz, Chloroform-*d*) δ 174.1, 171.6, 170.7, 167.7, 167.2, 167.2, 166.9, 134.9, 134.8, 134.6, 131.3, 131.2, 131.2, 130.9, 130.4, 130.3, 130.0, 129.9, 129.9, 129.9, 82.0, 76.6, 75.9, 73.2, 71.9, 71.6, 69.0, 64.5, 56.3, 53.7, 44.4, 42.5, 33.0, 31.1, 29.7, 29.5, 23.1.

LRMS(ESI): 946.4 $[M+Na]^+$

HRMS(ESI-TOF) m/z: $[M+H]^+$ Calcd for $C_{49}H_{54}N_3O_{15}$ 924.3549; Found 924.3548.

V. Scale-up Reaction and Further Transformation

Scheme S5. **Gram-scale synthesis**

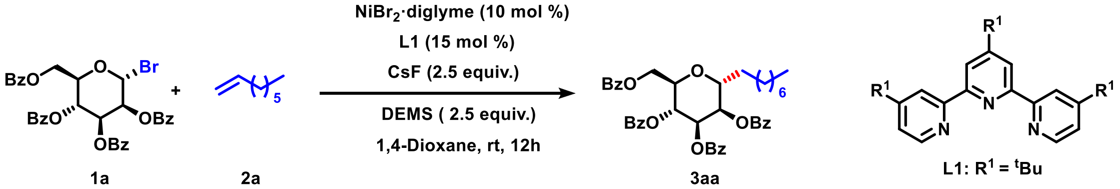

1a 2a 3aa

2g 0.78g

Mannosyl bromide **1a** (3.10 mmol, 2.0 equiv.), **L1** (15 mol%, 0.23 mmol), $NiBr_2$·diglyme (10 mol%, 0.16 mmol), and CsF (3.88 mmol, 2.5 equiv.) were sequentially added to a Schlenk tube under air, then the tube was evacuated and backfilled with argon three times. The mixture of 1,4-Dioxane (10 mL), octene **2a** (1.55 mmol) and DEMS (3.88 mmol) was added subsequently. The resulting mixture was stirred at room temperature for 12 h. After completion of the reaction, the mixture was filtered through a celite pad and washed with DCM (2.0 mL × 3). The combined organic layer was concentrated under reduced pressure and the crude residue was purified by preparative TLC to give the desired product **3aa** (0.78 g, 73%).

Scheme S6. **Removal of the protecting group**

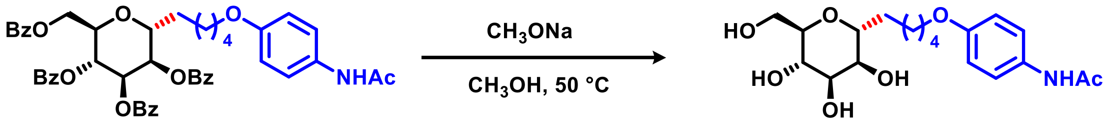

3an 4an 85%

Compound **3aa** was dissolved in CH_3OH (10 mL) in a 50 mL round bottom flask. Sodium methoxide was introduced into the solution to raise the pH of the solution above 13. The mixture was stirred at 50 °C for 6 h. 1N HCl was add to the solution with the pH of the solution to 7. Then the solution was concentrated under reduced pressure and purified on flash silica gel chromatography, giving product **4an** as a white solid in 85% yield.

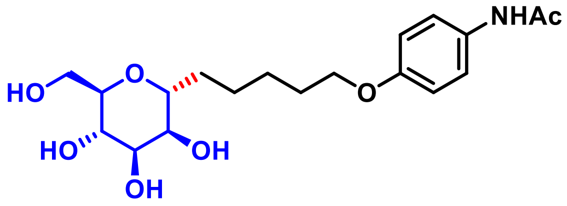

4an

***N*-(4-((5-((2*R*,3*S*,4*R*,5*S*,6*R*)-3,4,5-trihydroxy-6-(hydroxymethyl)tetrahydro-2*H*-pyran-2-yl)pentyl)oxy)phenyl)acetamide (4an)**

^{1}H NMR (600 MHz, Methanol-d_4) δ 7.43 (d, *J* = 9.0 Hz, 2H), 6.87 (d, *J* = 8.9 Hz, 2H), 3.97 (t, *J* = 6.4 Hz, 2H), 3.91 – 3.87 (m, 1H), 3.82 – 3.73 (m, 3H), 3.71 (dd, *J* = 9.0, 3.1 Hz, 1H), 3.67 (t, *J* = 8.9 Hz, 1H), 3.46 – 3.42 (m, 1H), 2.12 (s, 3H), 1.85 – 1.76 (m, 3H), 1.60 – 1.48 (m, 4H), 1.47 – 1.41 (m, 1H).

^{13}C NMR (125 MHz, Methanol-d_4) δ 170.0, 155.9, 131.2, 121.6, 114.1, 77.7, 77.6, 74.0, 71.7, 71.3, 67.7, 61.3, 28.9, 28.0, 25.5, 25.3, 22.1.

LRMS(ESI): 384.4 $[M+H]^+$, 401.1 $[M+NH_4]^+$

HRMS(ESI-TOF) m/z: $[M+H]^+$ Calcd for $C_{19}H_{30}NO_7$ 384.2017; Found 384.2019.

VI. Mechanistic Investigation

1. Radical Trapping Experiment

Scheme S7. Radical trapping experiment with TEMPO

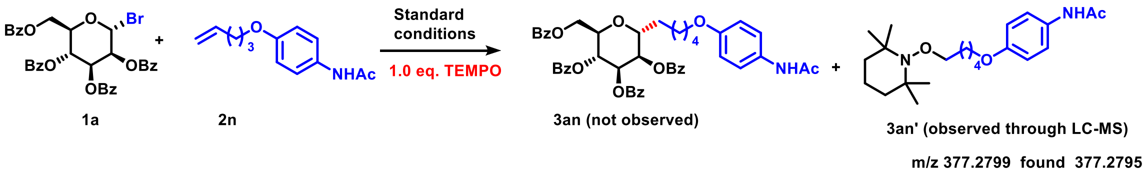

Mannosyl bromide **1a** (0.4 mmol, 2.0 equiv.), **L1** (15 mol%, 0.03 mmol), $NiBr_2$·diglyme (10 mol%, 0.02 mmol), CsF (0.5 mmol, 2.5 equiv.) and TEMPO (0.2 mmol, 1.0 equiv.) were sequentially added to a Schlenk tube under air, then the tube was evacuated and backfilled with argon three times, then the tube was evacuated and backfilled with argon three times. The mixture of 1,4-Dioxane (1 mL), octene **2a** (0.2 mmol) and DEMS (0.5 mmol) was added subsequently. The resulting mixture was stirred at room temperature for 12 h. After completion of the reaction, the mixture was filtered through a celite pad and washed with DCM (2.0 mL × 3). The combined organic layer was concentrated under reduced pressure and the crude residue was purified by preparative TLC to give the product **3an'** only.

2. Deuterium Labelling Experiment

Scheme S8. Deuterium labelling experiment with Ph_2SiD_2

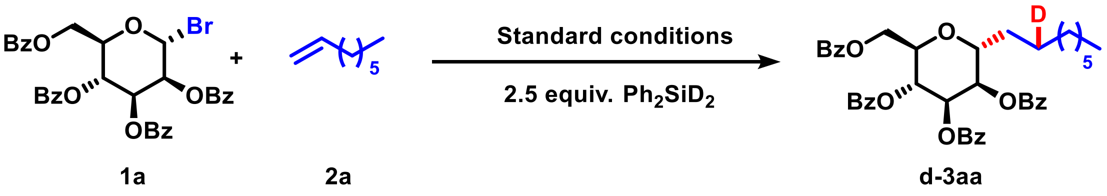

Mannosyl bromide **1a** (0.4 mmol, 2.0 equiv.), **L1** (15 mol%, 0.03 mmol), $NiBr_2$·diglyme (10 mol%, 0.02 mmol), and CsF (0.5 mmol, 2.5 equiv.) were sequentially added to a Schlenk tube under air, then the tube was evacuated and backfilled with argon three times. The mixture of 1,4-Dioxane (1 mL), octene **2a** (0.2 mmol) and Ph_2SiD_2 (0.5 mmol) was added subsequently. The resulting mixture was stirred at room temperature for 12 h. After completion of the reaction, the mixture was filtered through a celite pad and washed with DCM (2.0 mL × 3). The combined organic layer was concentrated under reduced pressure and the crude residue was purified by

preparative TLC to give the product **d-3aa**.

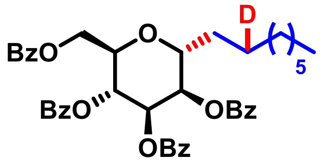

d-3aa

2-((benzoyloxy)methyl)-6-(octyl-2-d)tetrahydro-2*H*-pyran-3,4,5-triyl tribenzoate (d-3aa)

^{1}H NMR (500 MHz, Acetone-d_6) δ 8.11 (dd, *J* = 8.3, 1.4 Hz, 2H), 8.05 (dd, *J* = 8.2, 1.4 Hz, 2H), 8.00 (dd, *J* = 8.3, 1.4 Hz, 2H), 7.82 (dd, *J* = 8.3, 1.4 Hz, 2H), 7.71 – 7.62 (m, 2H), 7.61 – 7.54 (m, 1H), 7.56 – 7.46 (m, 5H), 7.43 (t, *J* = 7.8 Hz, 2H), 7.34 (t, *J* = 7.9 Hz, 2H), 6.06 (t, *J* = 9.4 Hz, 1H), 5.91 (dd, *J* = 9.5, 3.3 Hz, 1H), 5.73 (t, *J* = 2.8 Hz, 1H), 4.71 – 4.60 (m, 2H), 4.54 (ddd, *J* = 9.3, 4.8, 2.9 Hz, 1H), 4.28 (ddd, *J* = 10.7, 4.8, 2.3 Hz, 1H), 2.27 – 2.15 (m, 1H), 1.90 – 1.80 (m, 1H), 1.66 – 1.56 (m, 1H), 1.48 – 1.37 (m, 2H), 1.34 – 1.24 (m, 8H), 0.92 – 0.82 (m, 3H).

^{13}C NMR (125 MHz, Chloroform-d) δ 166.4, 165.9, 165.6, 133.6, 133.5, 133.4, 133.2, 130.0, 130.0, 129.9, 129.8, 129.2, 128.7, 128.6, 128.5, 76.0, 72.3, 70.4, 70.4, 67.9, 63.5, 32.0, 29.6, 29.6, 29.4, 29.3, 29.3, 28.7, 28.6, 25.7, 22.8, 14.3.

LRMS(ESI): 711.0 $[M+NH_4]^+$.

HRMS(ESI-TOF) m/z: $[M+H]^+$ Calcd for $C_{42}H_{44}DO_9$ 694.3121; Found 694.3121.

VII. References

(1) (a)Wu, J.; Wei, W.; Poehlmann, J.; Purushothaman, R.; Ackermann, L. Domino meta-C-H Ethyl Glycosylation by Ruthenium(II/III) Catalysis: Modular Assembly of meta-*C*-Alkyl Glycosides. *Angew Chem Int Ed.* **2023**, *62*. e202219319. (b) Wu, J.; Kaplaneris, N.; Pohlmann, J.; Michiyuki, T.; Yuan, B. B.; Ackermann, L. Remote C-H Glycosylation by Ruthenium(II) Catalysis: Modular Assembly of meta-C-Aryl Glycosides. *Angew Chem Int Ed.* **2022**, *61*. e202208620. (c) M. Emmadi, S. S. Kulkarni. Total synthesis of the bacillosamine containing α-l-serine linked trisaccharide of Neisseria meningitidis. Carbohyd. Res. **2014**, 399, 57.

VII. NMR Spectra

(2*R*,3*R*,4*R*,5*R*,6*R*)-2-((benzoyloxy)methyl)-6-octyltetrahydro-2*H*-pyran-3,4,5-triyl tribenzoate (3aa)

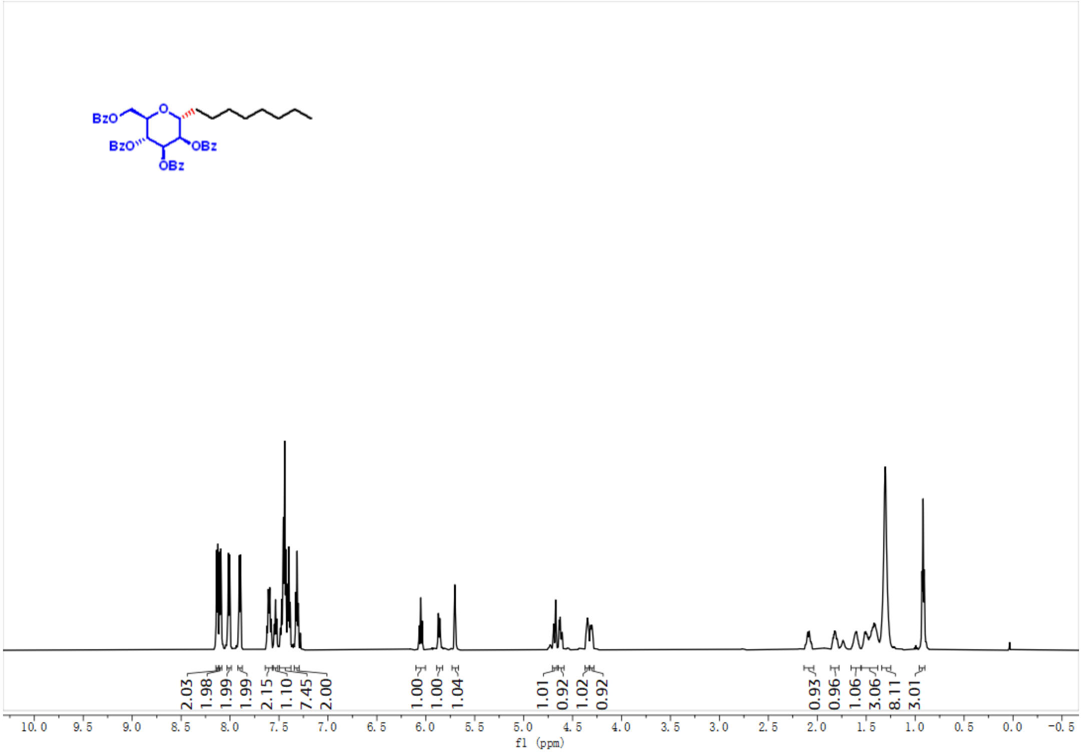

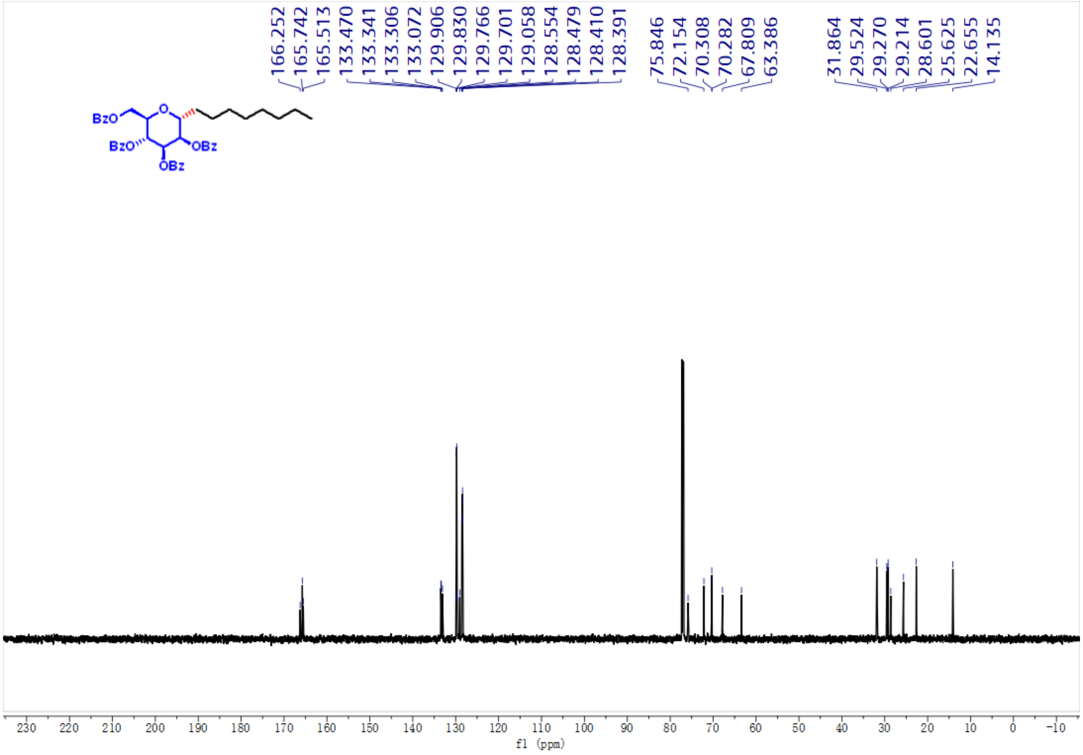

(2*R*,3*R*,4*R*,5*R*,6*R*)-2-((benzoyloxy)methyl)-6-hexyltetrahydro-2*H*-pyran-3,4,5-triyl tribenzoate (3ab)

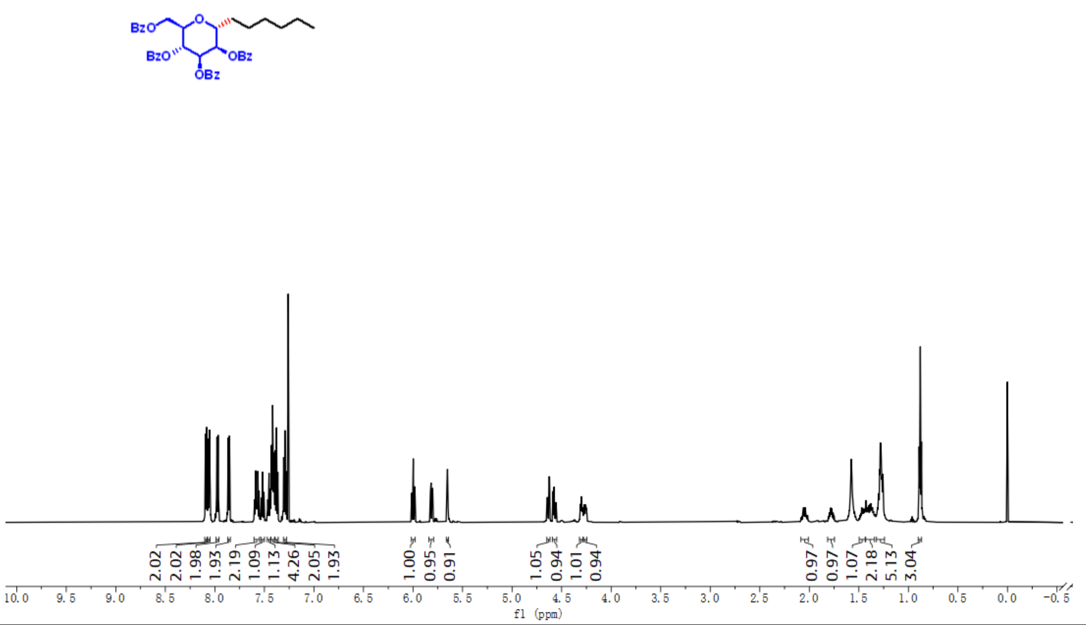

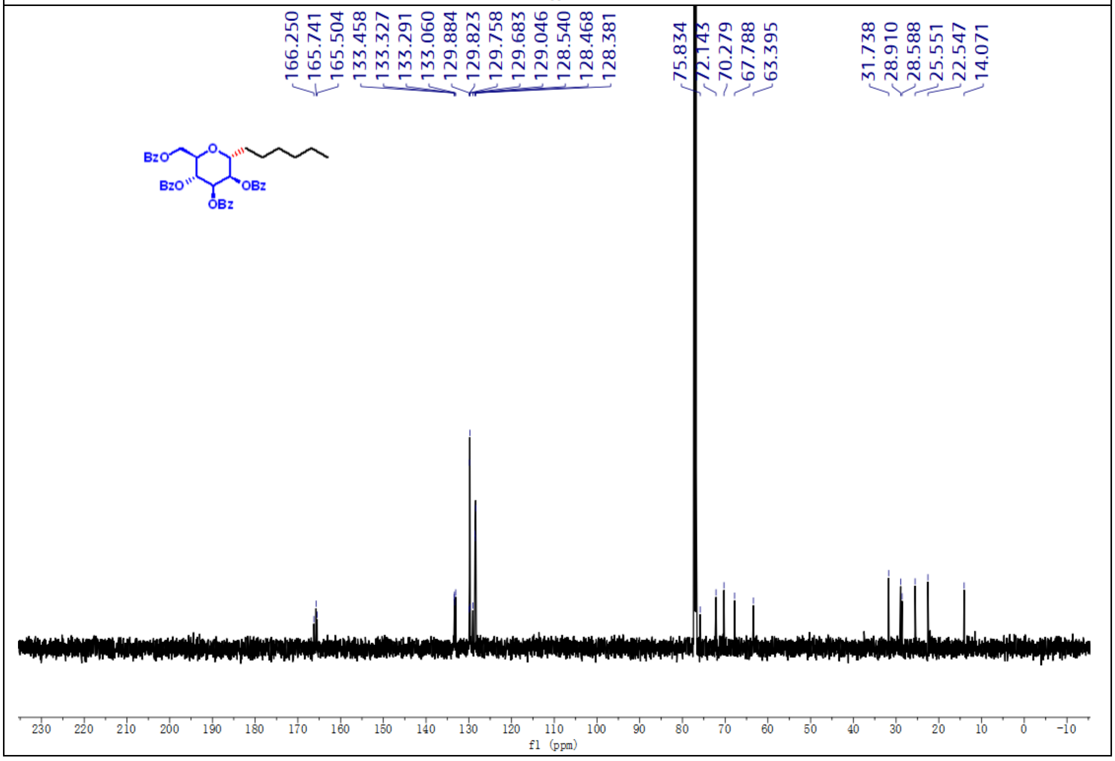

(2*R*,3*R*,4*R*,5*R*,6*R*)-2-((benzoyloxy)methyl)-6-heptyltetrahydro-2*H*-pyran-3,4,5-triyl tribenzoate (3ac)

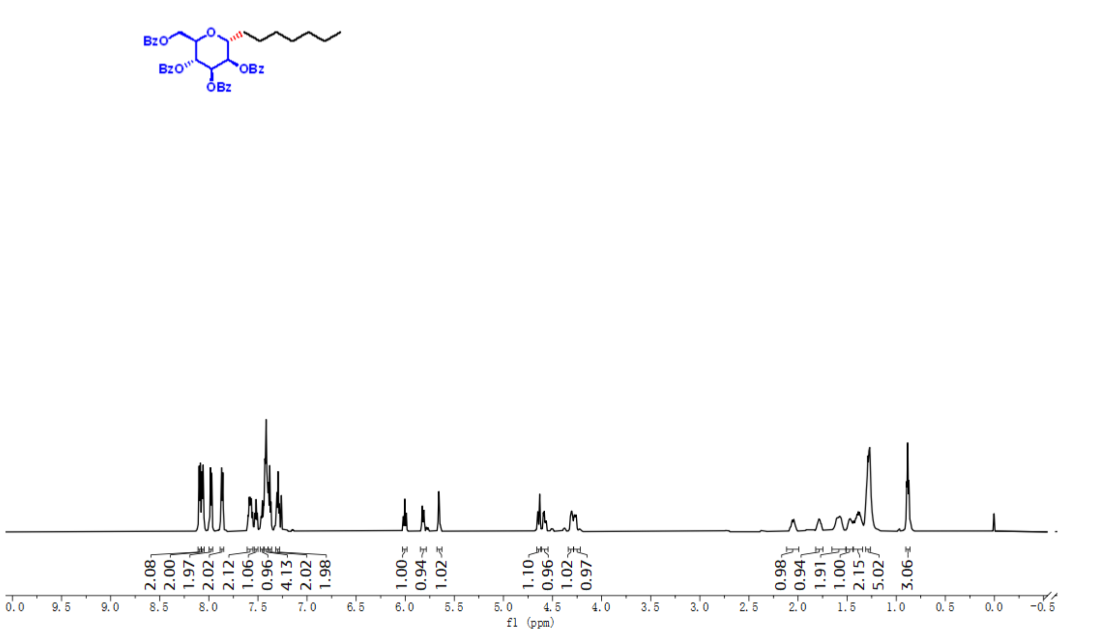

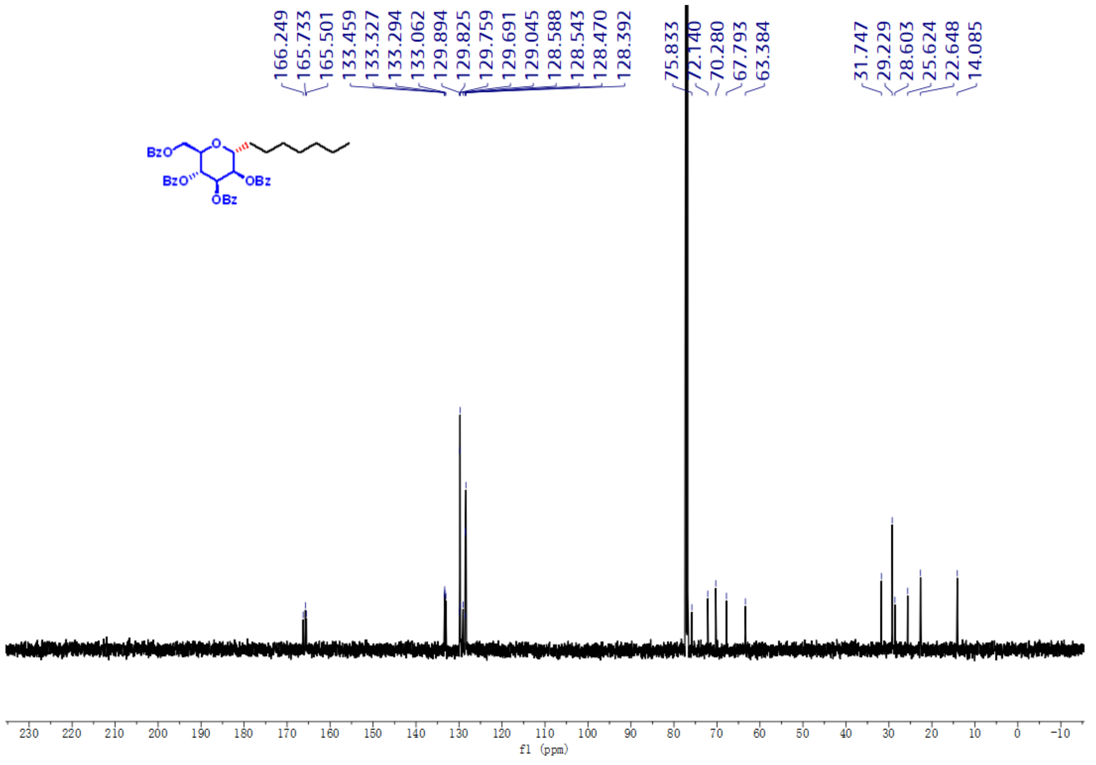

(2*R*,3*R*,4*R*,5*R*,6*R*)-2-((benzoyloxy)methyl)-6-(2-cyclopentylethyl)tetrahydro-2*H*-pyran-3,4,5-triyl tribenzoate (3ad)

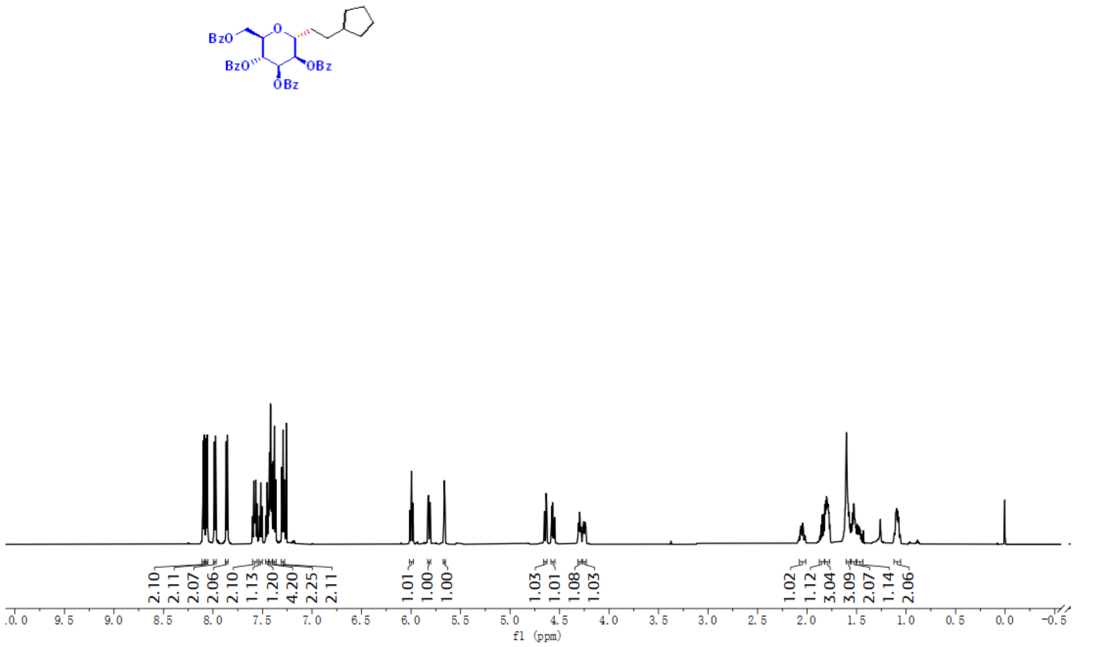

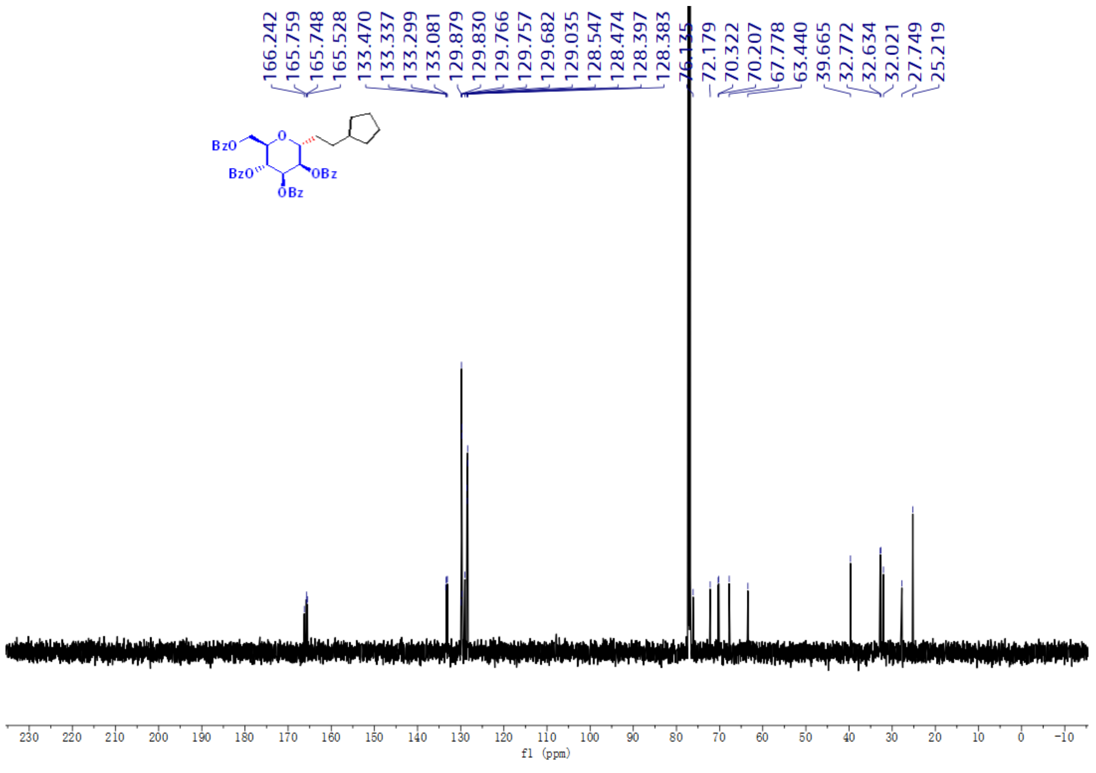

(2*R*,3*R*,4*R*,5*R*,6*R*)-2-((benzoyloxy)methyl)-6-(2-cyclohexylethyl)tetrahydro-2*H*-pyran-3,4,5-triyl tribenzoate (3ae)

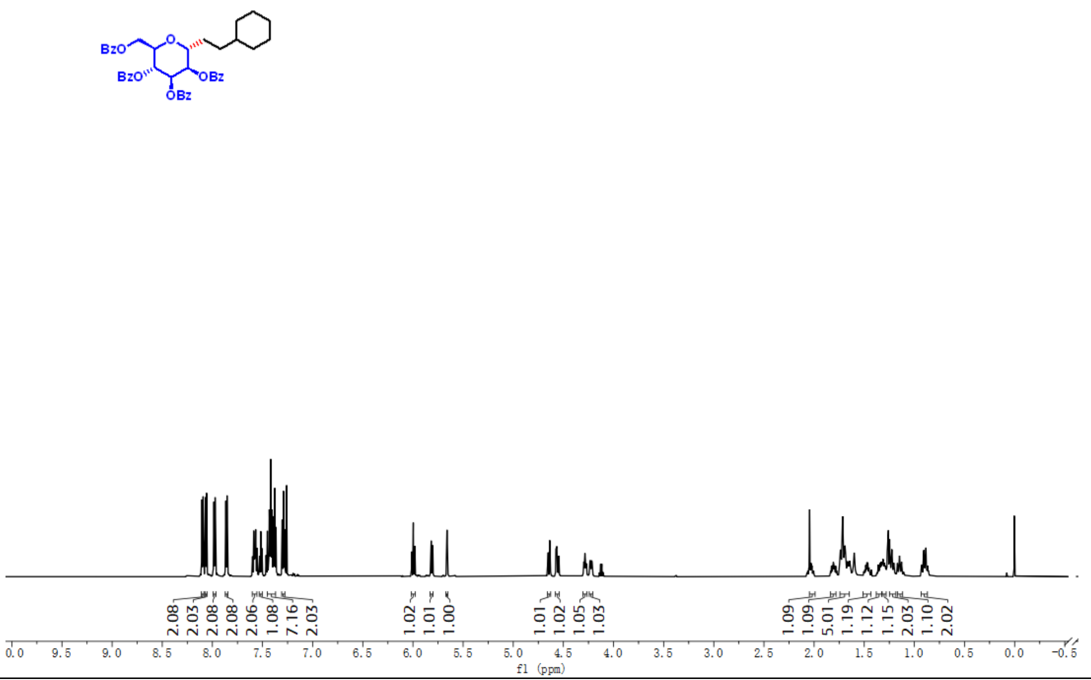

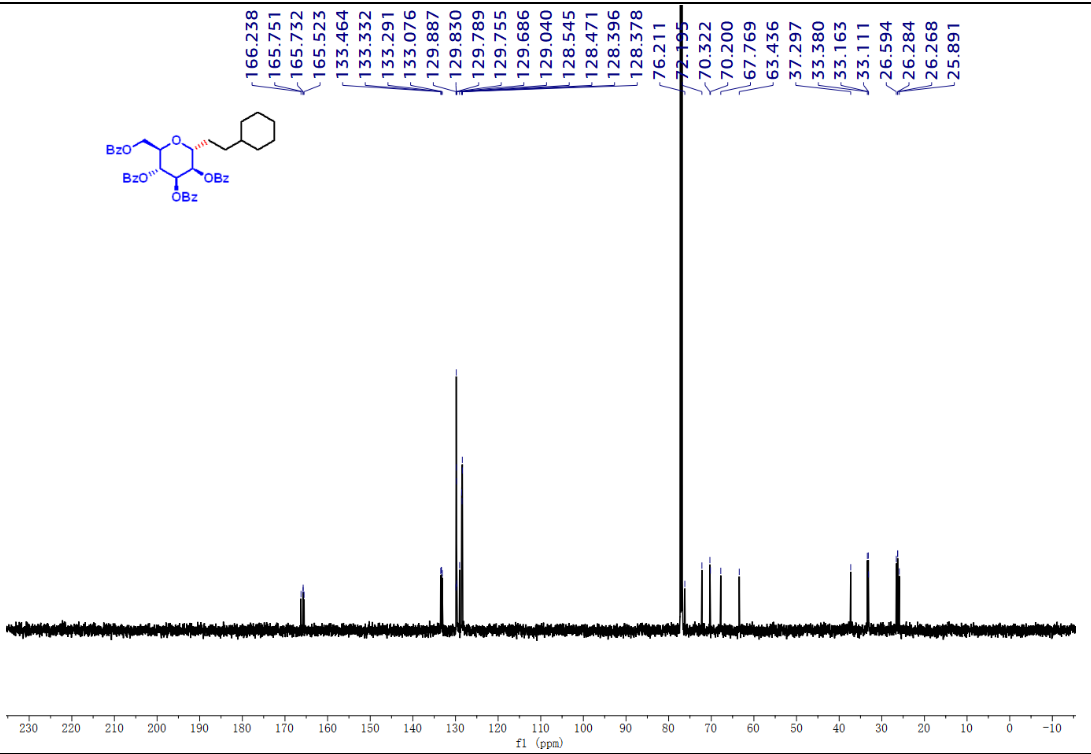

(2*R*,3*R*,4*R*,5*R*,6*R*)-2-((benzoyloxy)methyl)-6-(3-phenylpropyl)tetrahydro-2*H*-pyran-3,4,5-triyl tribenzoate (3af)

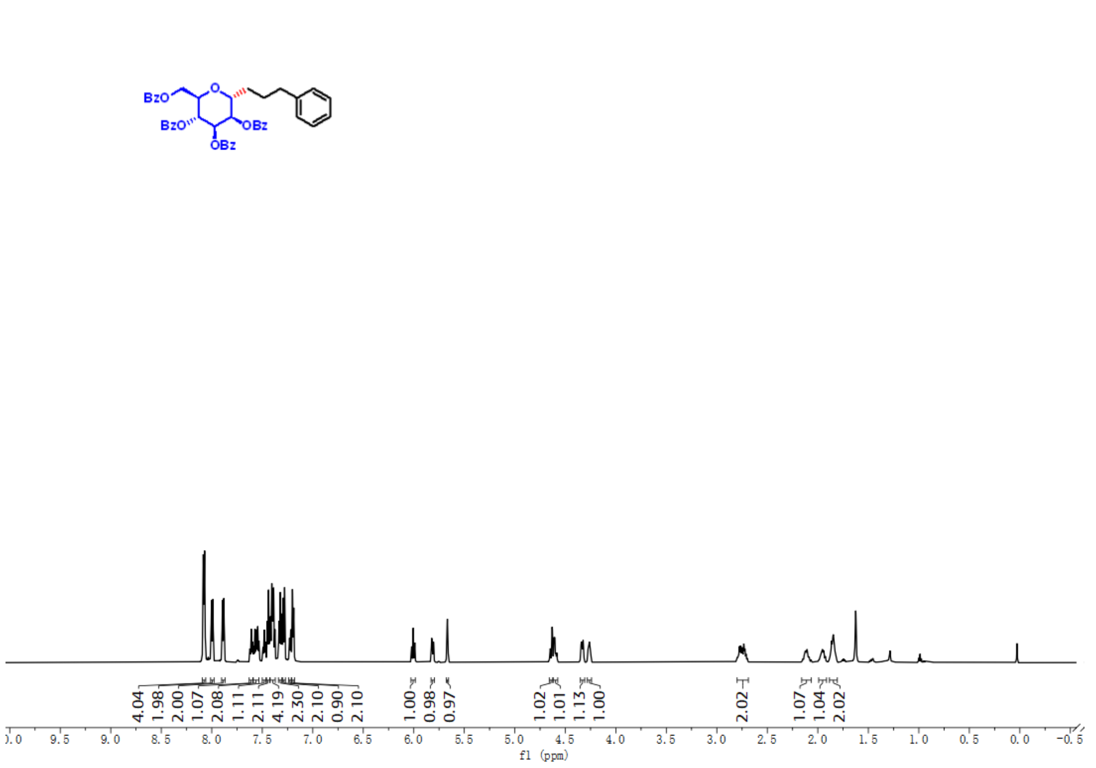

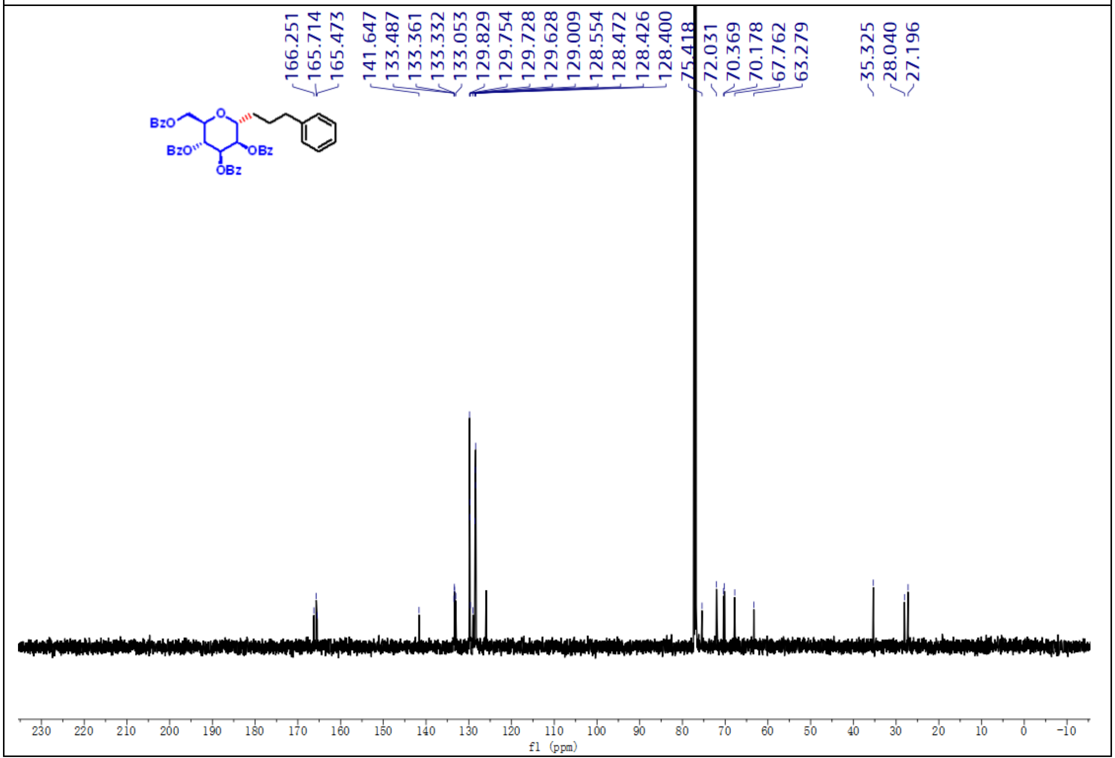

(2*R*,3*R*,4*R*,5*R*,6*R*)-2-((benzoyloxy)methyl)-6-(3-(o-tolyl)propyl)tetrahydro-2*H*-pyran-3,4,5-triyl tribenzoate (3ag)

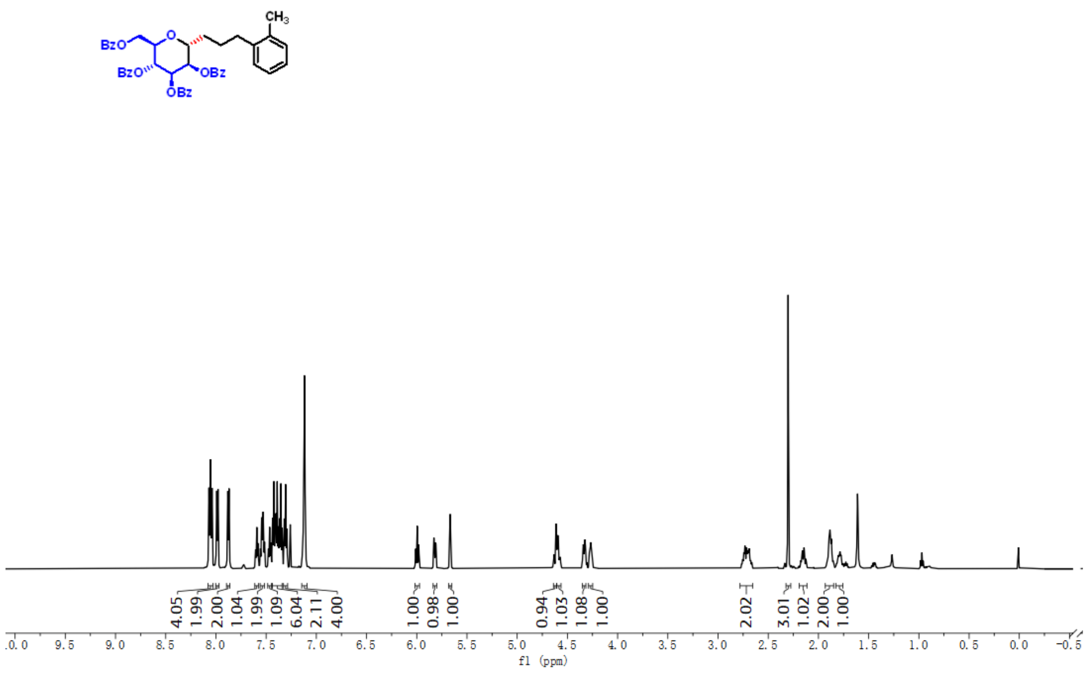

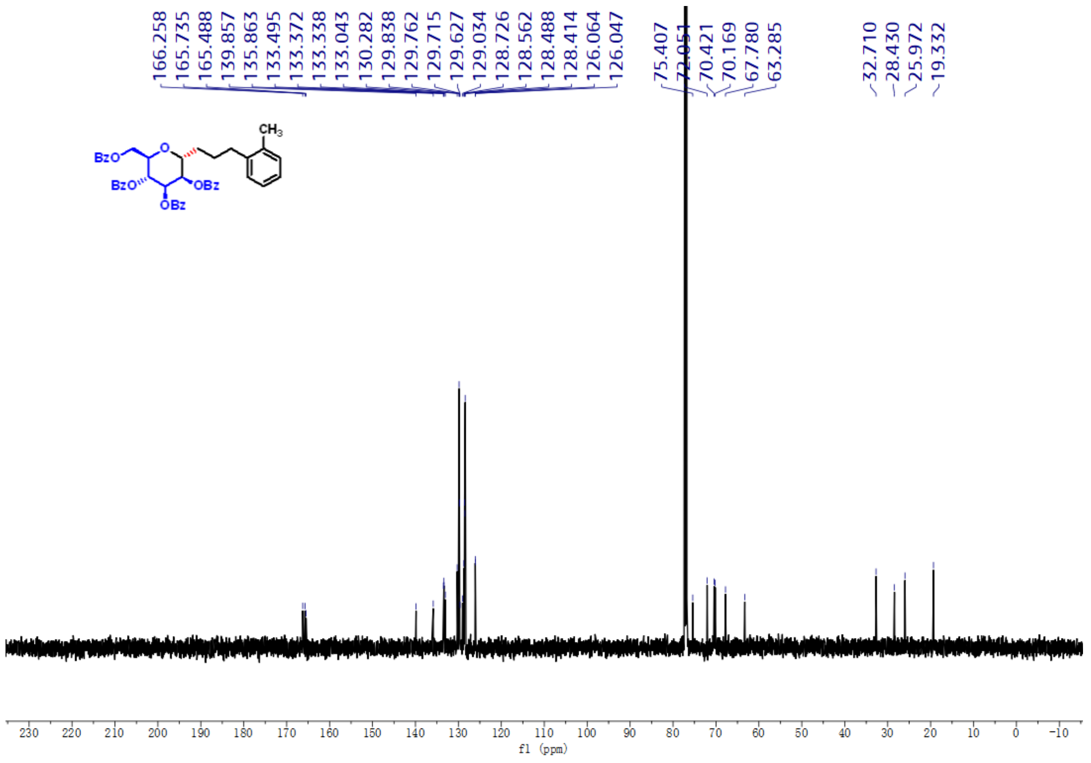

(2*R*,3*R*,4*R*,5*R*,6*R*)-2-((benzoyloxy)methyl)-6-(4-phenylbutyl)tetrahydro-2*H*-pyran-3,4,5-triyl tribenzoate (3ah)

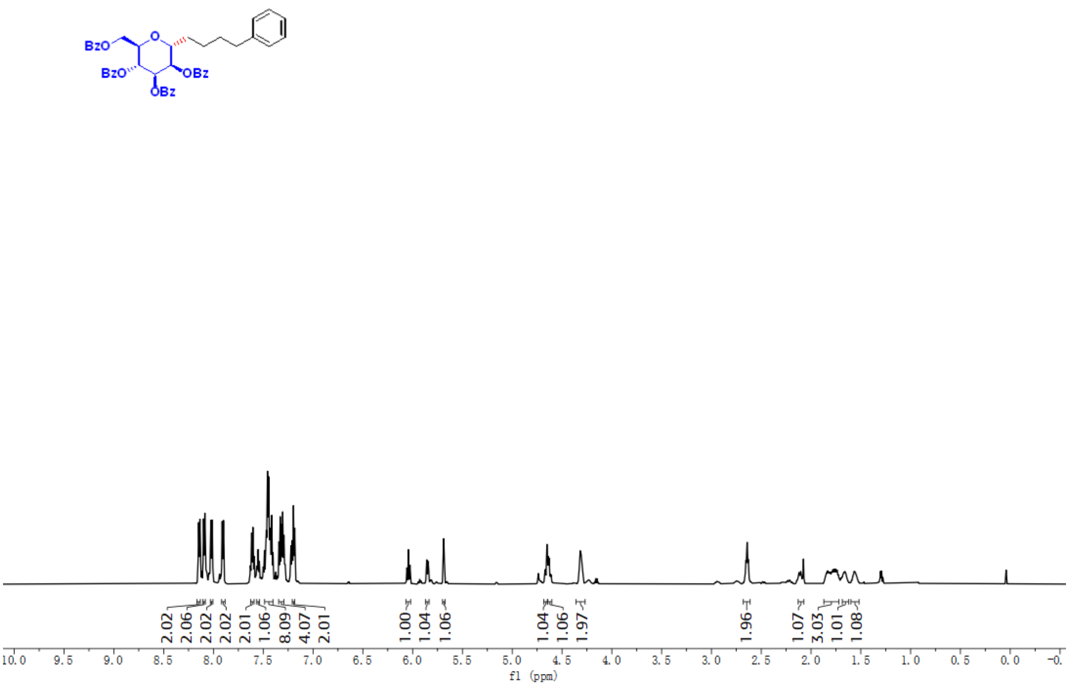

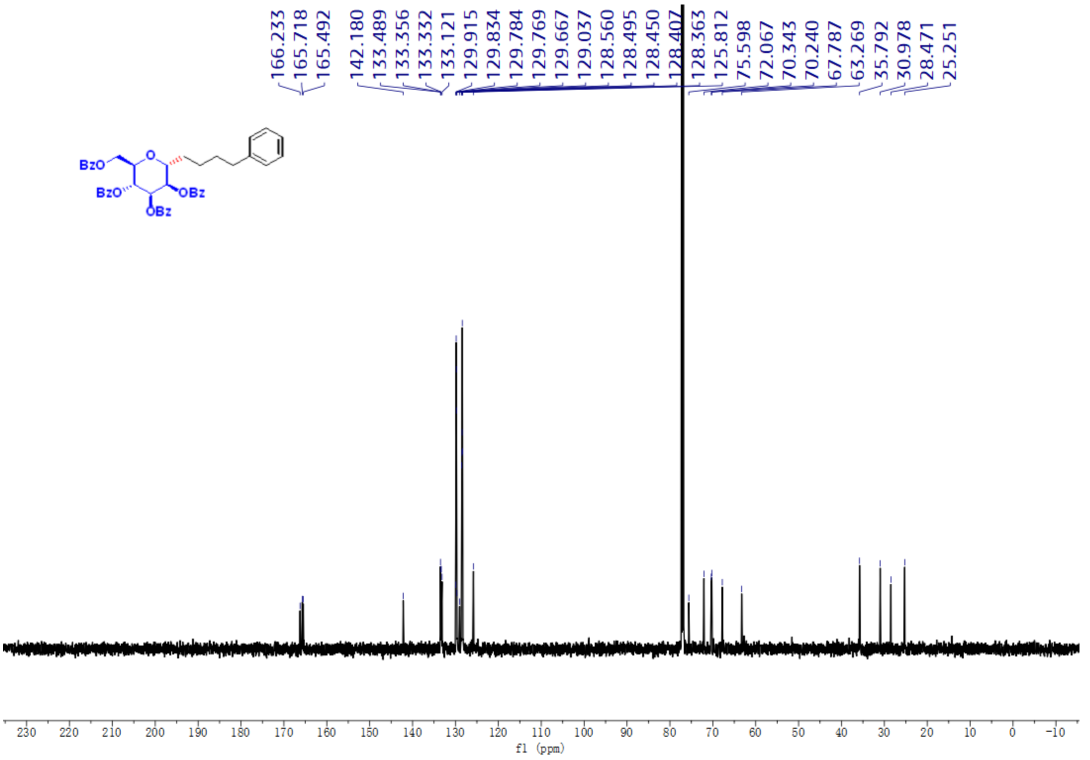

(2*R*,3*R*,4*R*,5*R*,6*R*)-2-((benzoyloxy)methyl)-6-(5-bromopentyl)tetrahydro-2*H*-pyran-3,4,5-triyl tribenzoate (3ai)

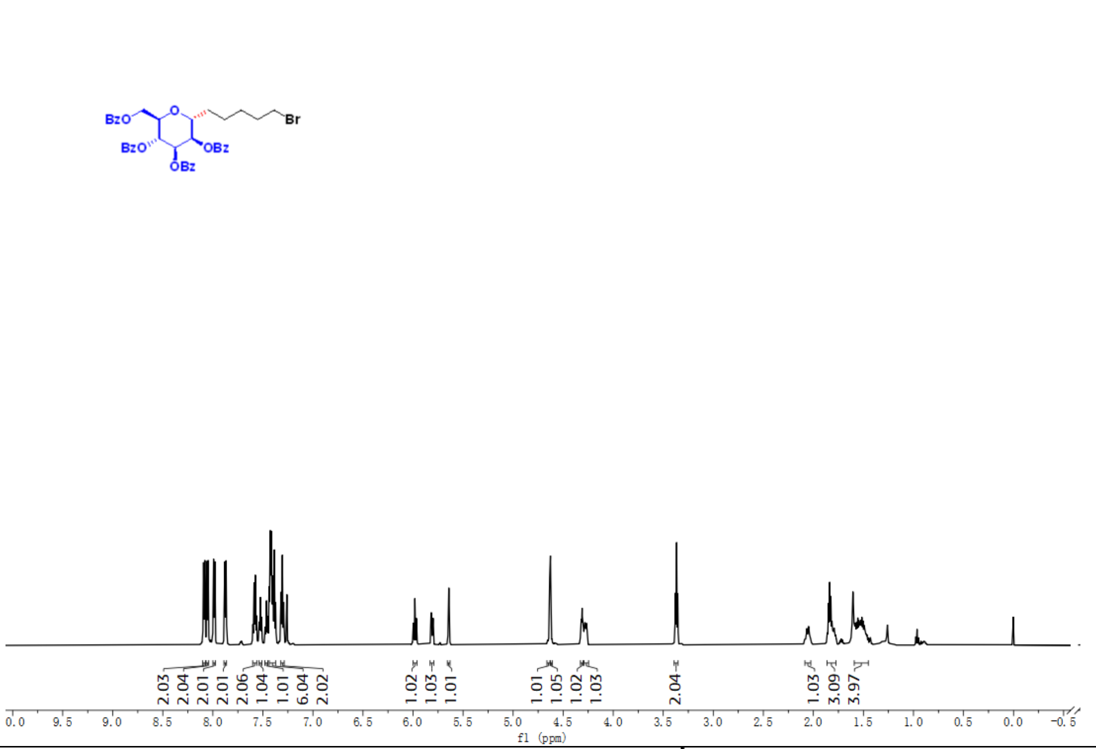

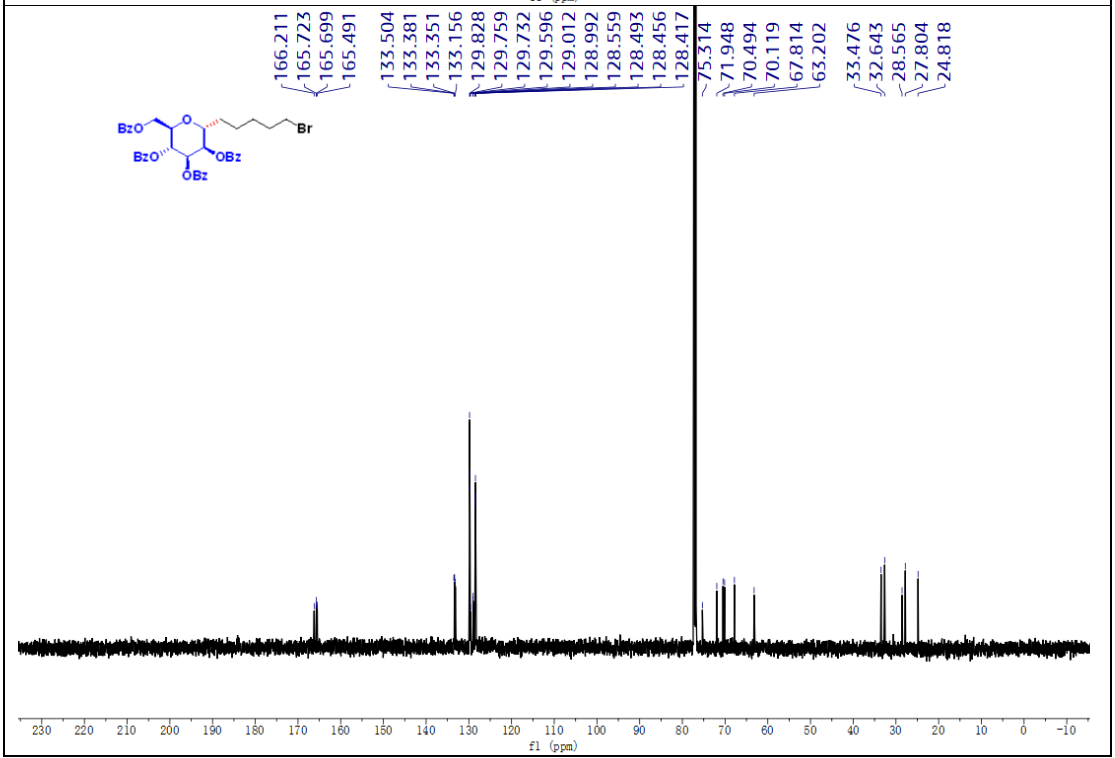

(2*R*,3*R*,4*R*,5*R*,6*R*)-2-((benzoyloxy)methyl)-6-(6-bromohexyl)tetrahydro-2*H*-pyran-3,4,5-triyl tribenzoate (3aj)

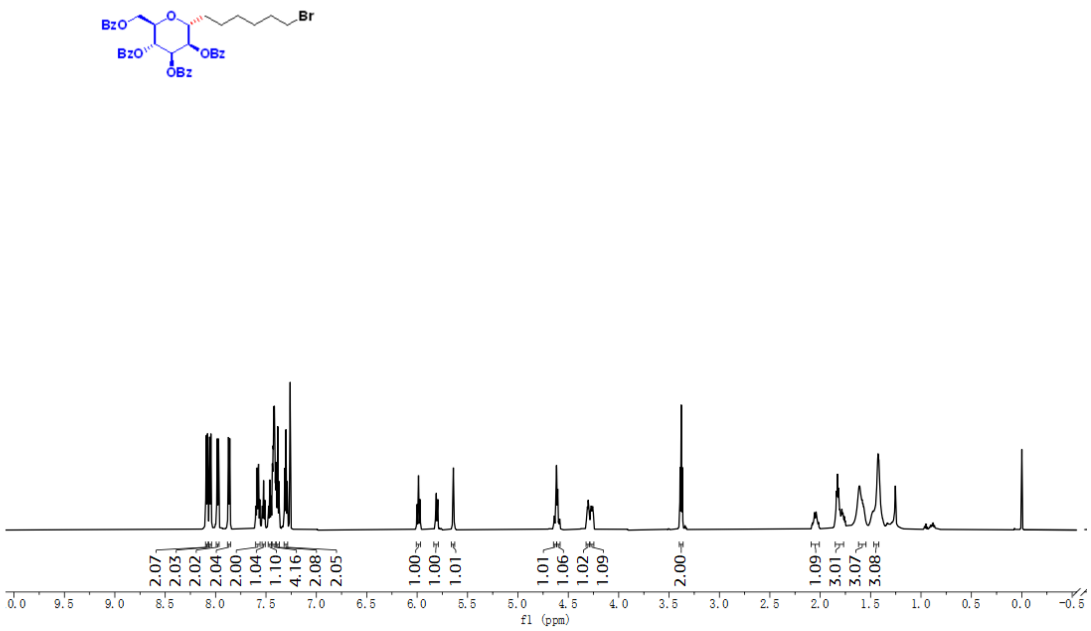

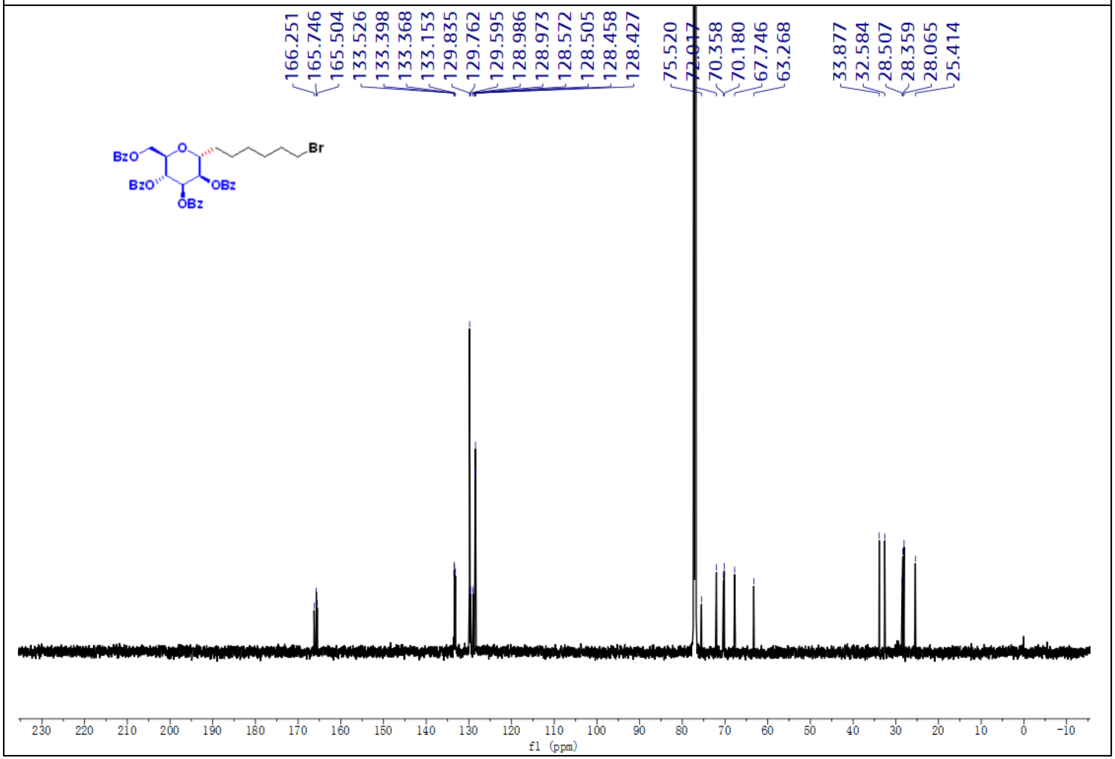

(2*R*,3*R*,4*R*,5*R*,6*R*)-2-((benzoyloxy)methyl)-6-(7-bromoheptyl)tetrahydro-2*H*-pyran-3,4,5-triyl tribenzoate (3ak)

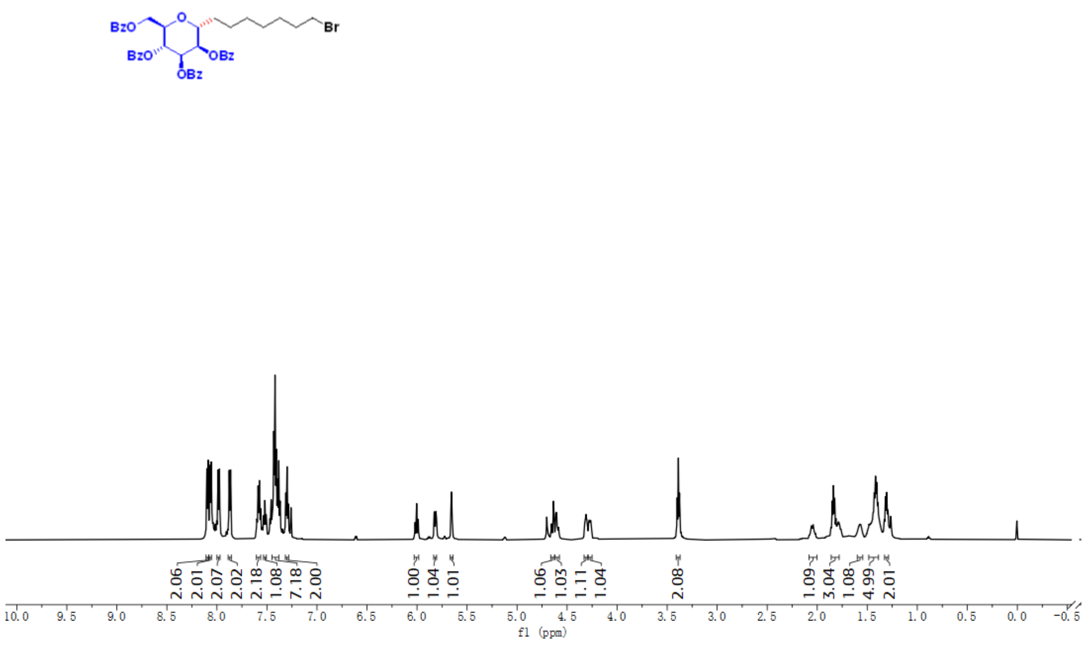

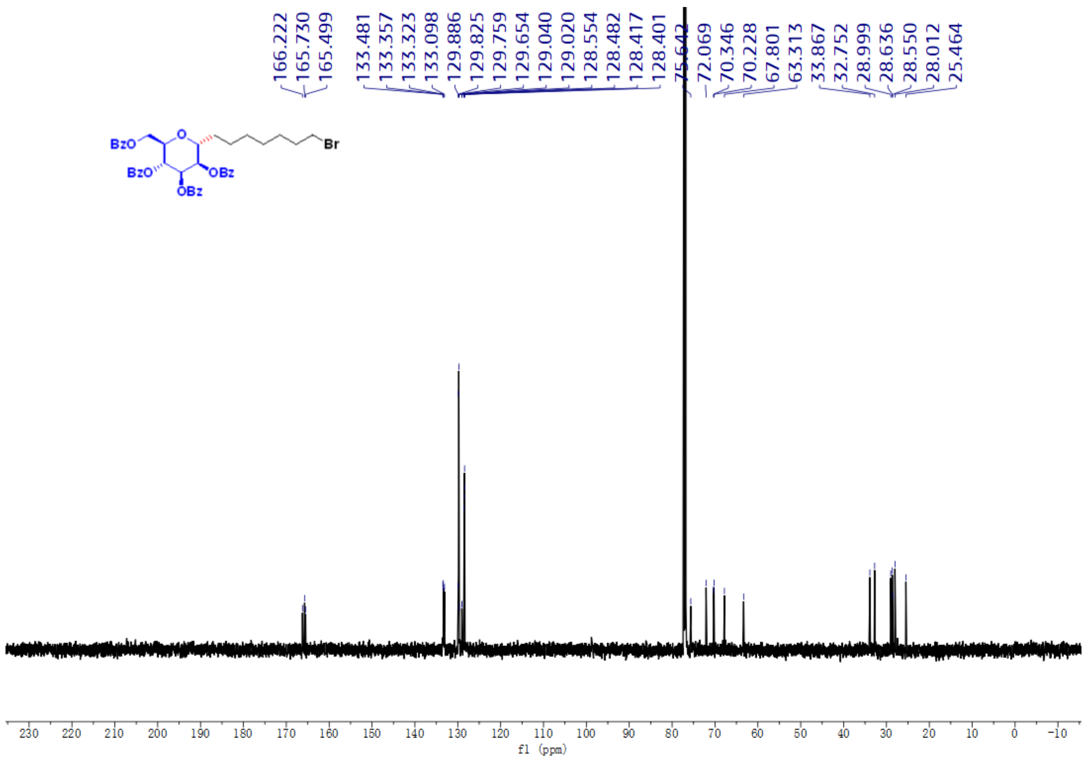

(2*R*,3*R*,4*R*,5*R*,6*R*)-2-((benzoyloxy)methyl)-6-(5-chloropentyl)tetrahydro-2*H*-pyran-3,4,5-triyl tribenzoate (3al)

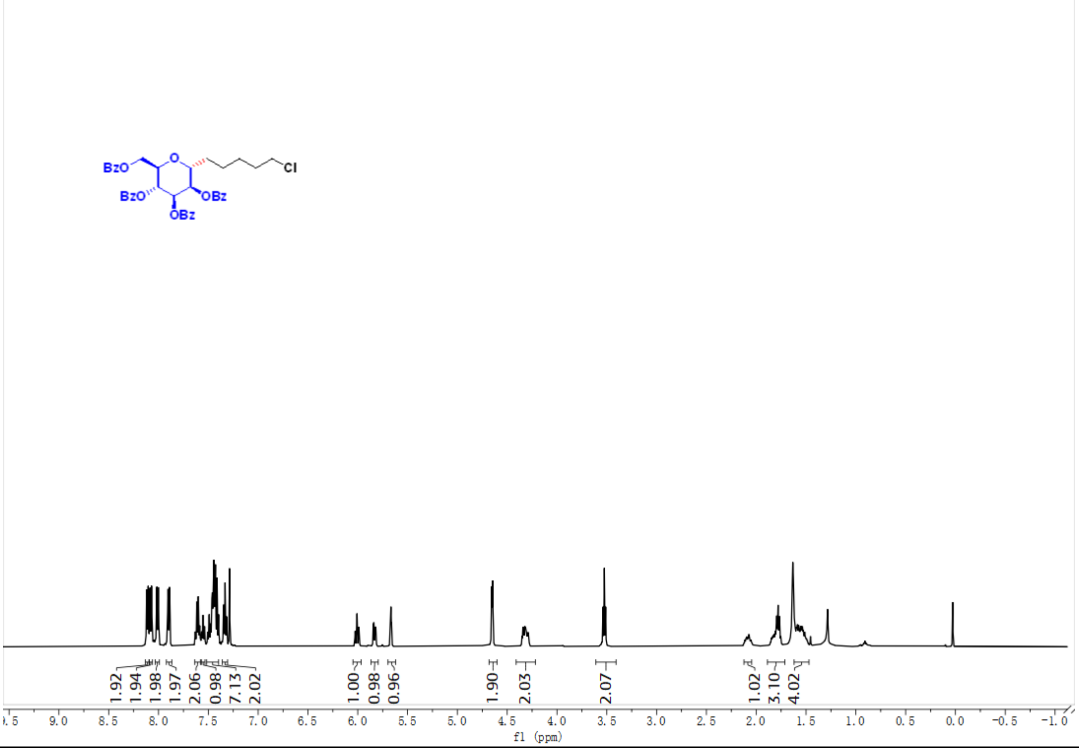

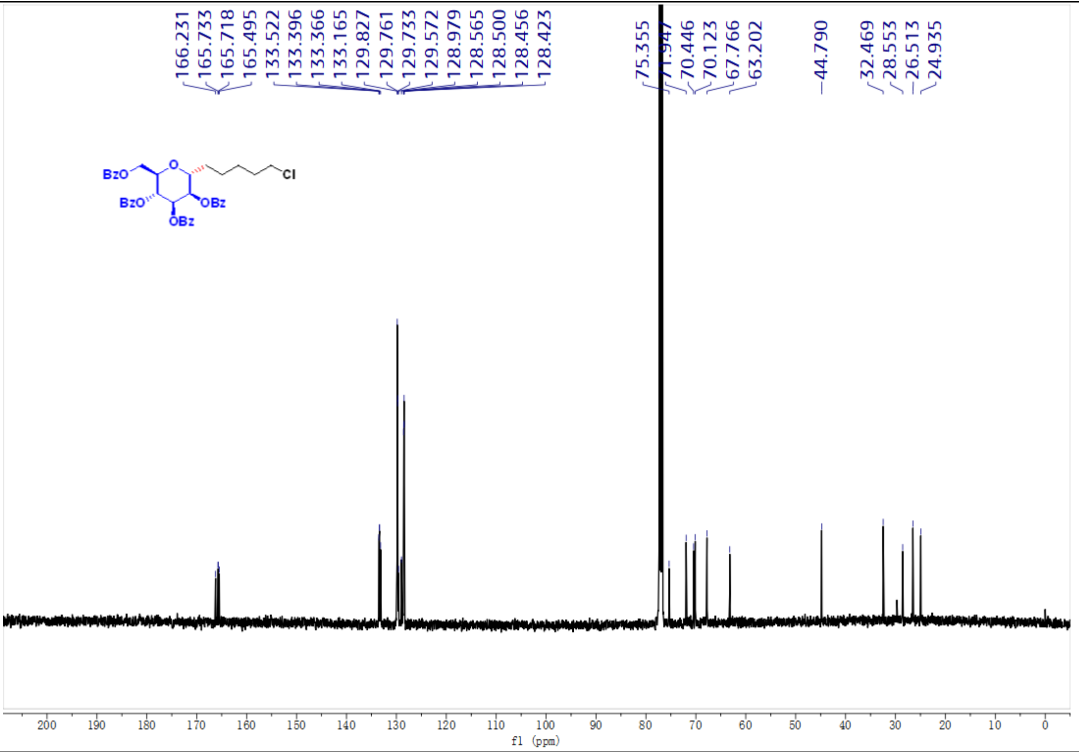

(2*R*,3*R*,4*R*,5*R*,6*R*)-2-((benzoyloxy)methyl)-6-(5-(4-cyanophenoxy)pentyl)tetrahydro-2*H*-pyran-3,4,5-triyl tribenzoate (3am)

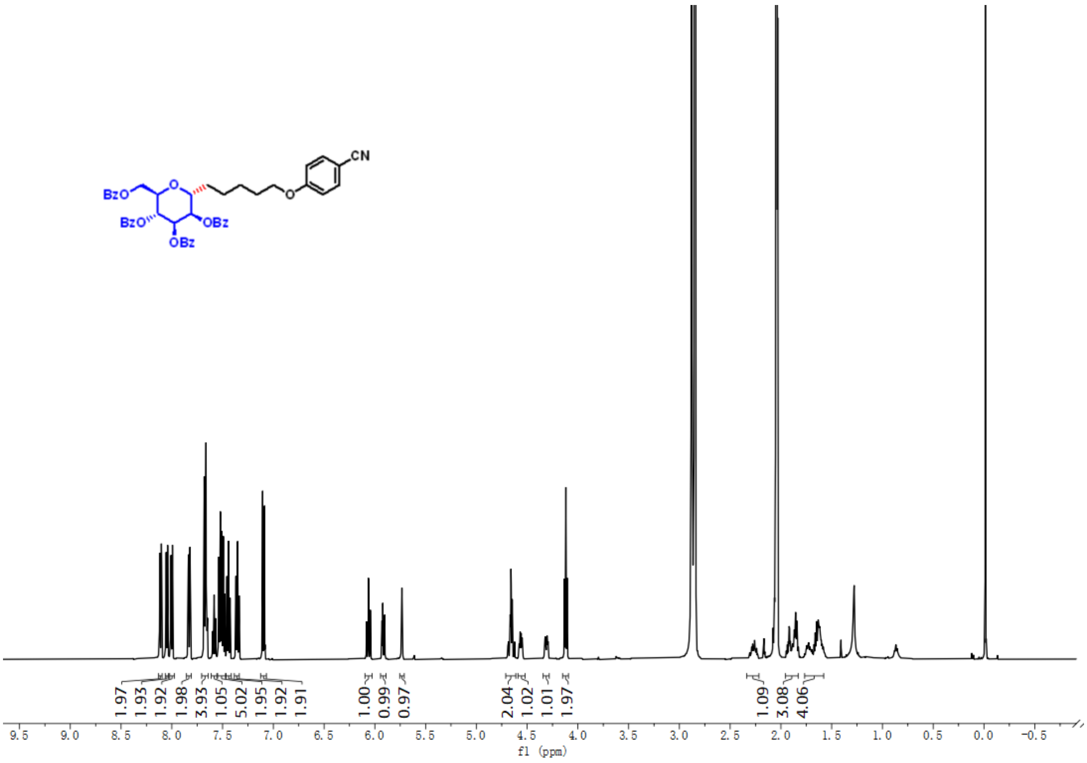

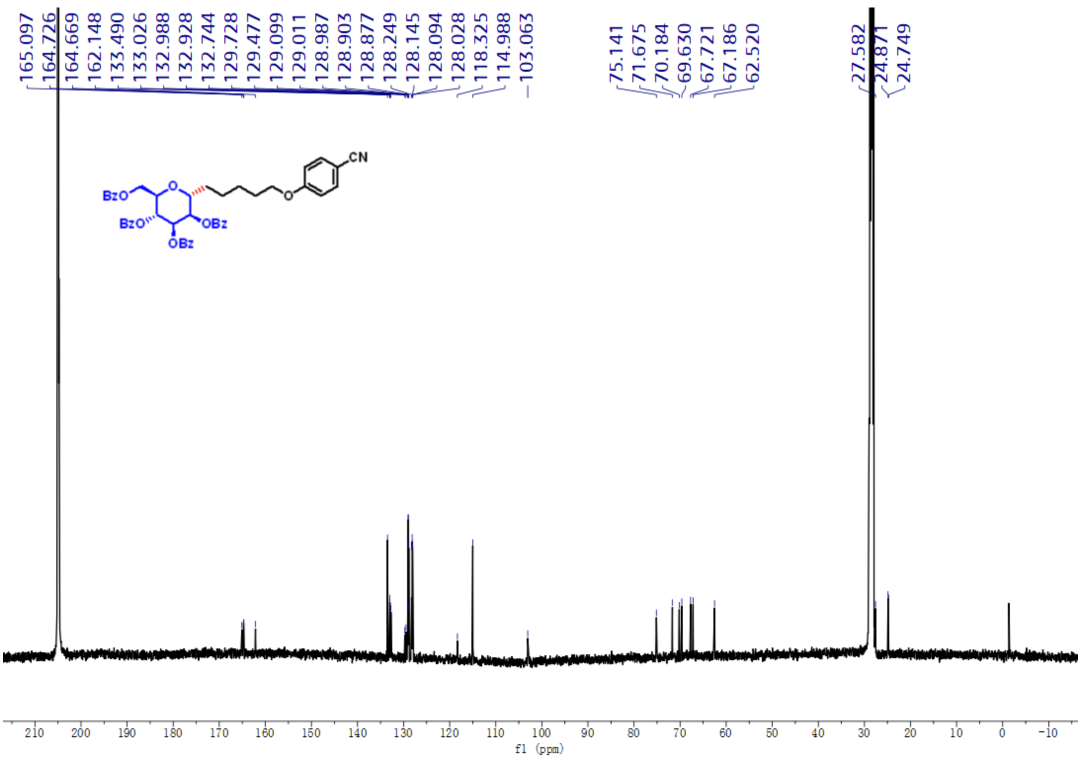

(2*R*,3*R*,4*R*,5*R*,6*R*)-2-(5-(4-acetamidophenoxy)pentyl)-6-((benzoyloxy)methyl)tetrahydro-2*H*-pyran-3,4,5-triyl tribenzoate (3an)

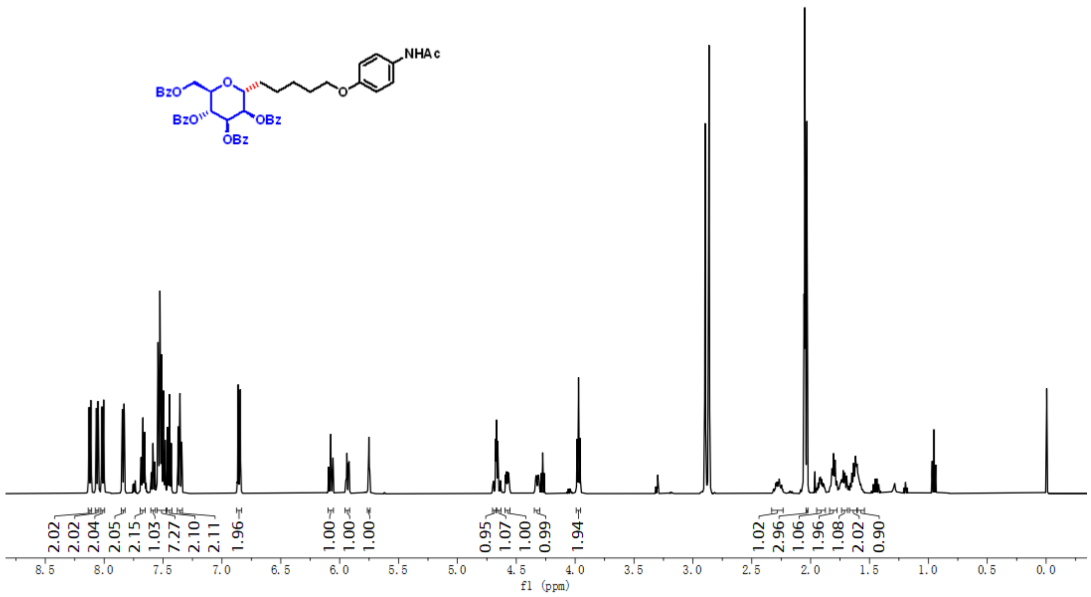

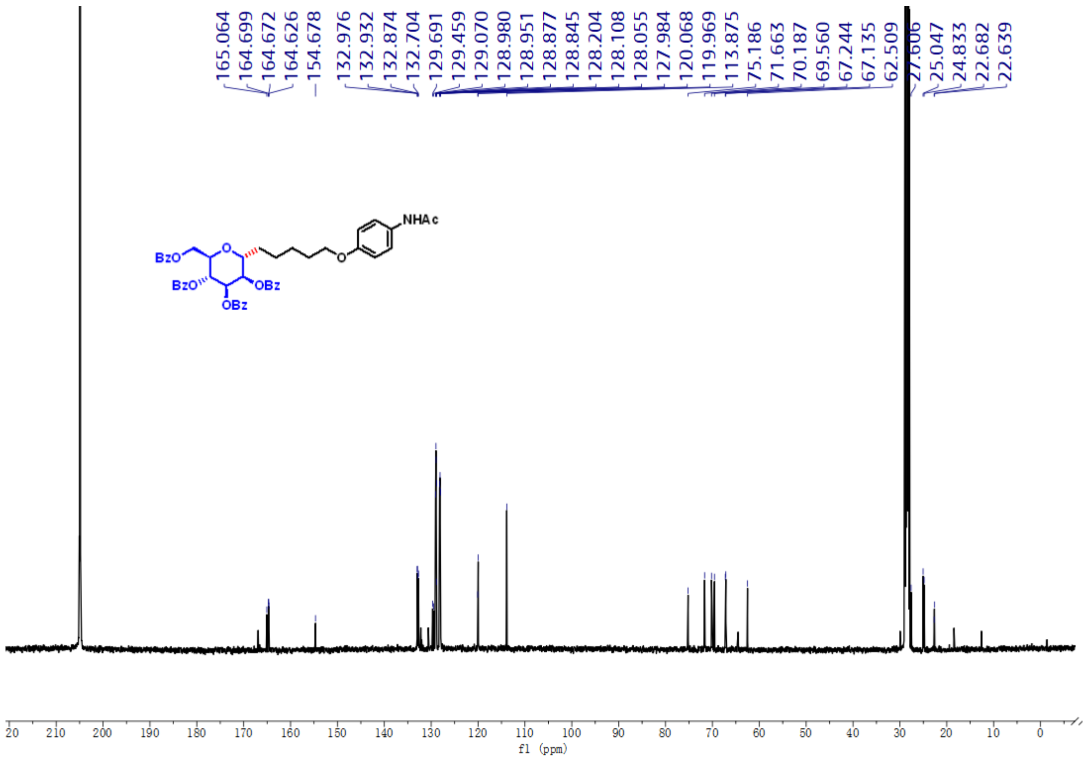

(2*R*,3*R*,4*R*,5*R*,6*R*)-2-((benzoyloxy)methyl)-6-(5-((furan-2-carbonyl)oxy)pentyl)tetrahydro-2*H*-pyran-3,4,5-triyl tribenzoate (3ao)

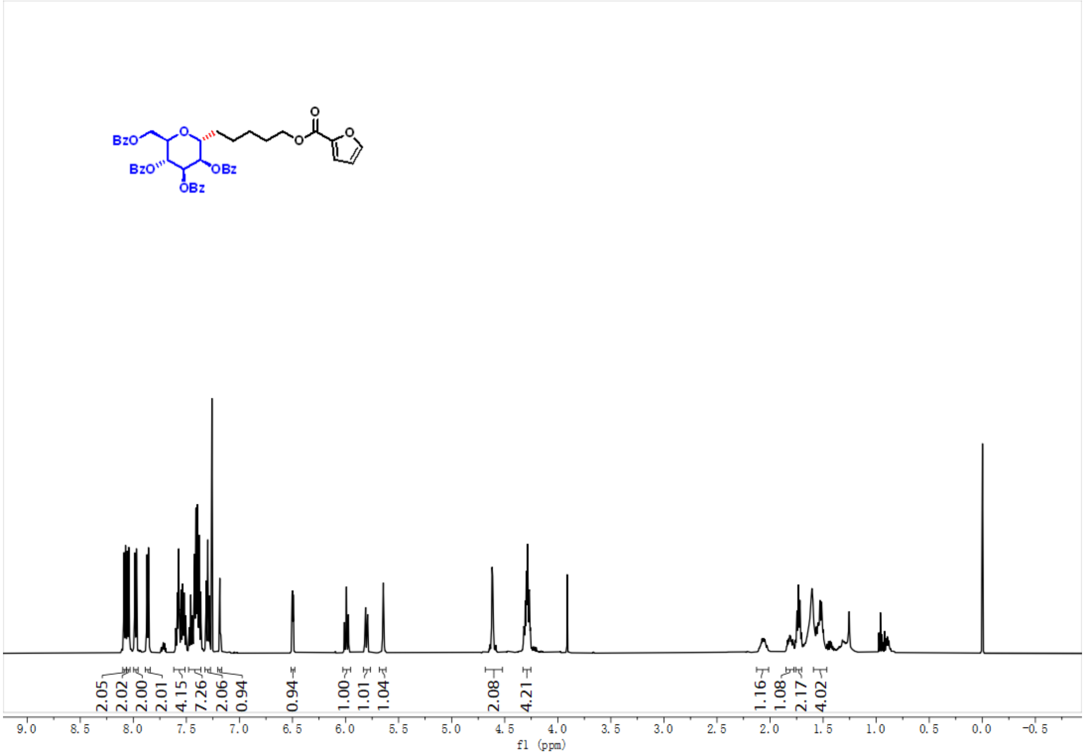

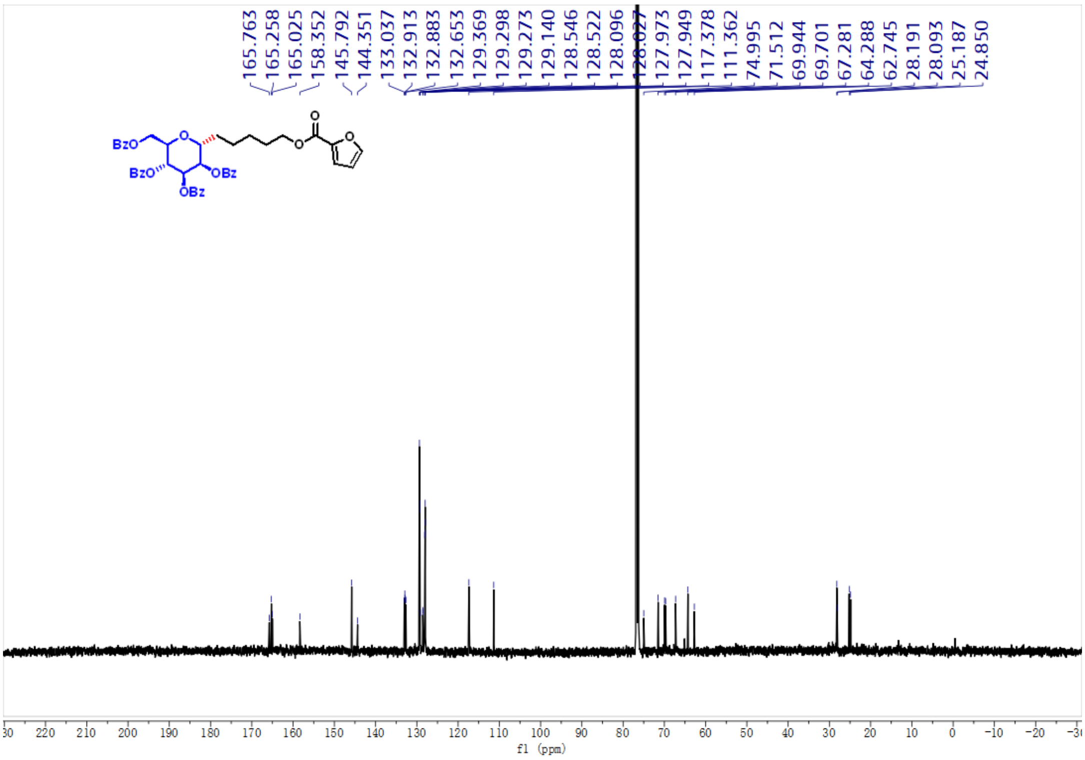

(2*R*,3*R*,4*R*,5*R*,6*R*)-2-((benzoyloxy)methyl)-6-(5-oxohexyl)tetrahydro-2*H*-pyran-3,4,5-triyl tribenzoate (3ap)

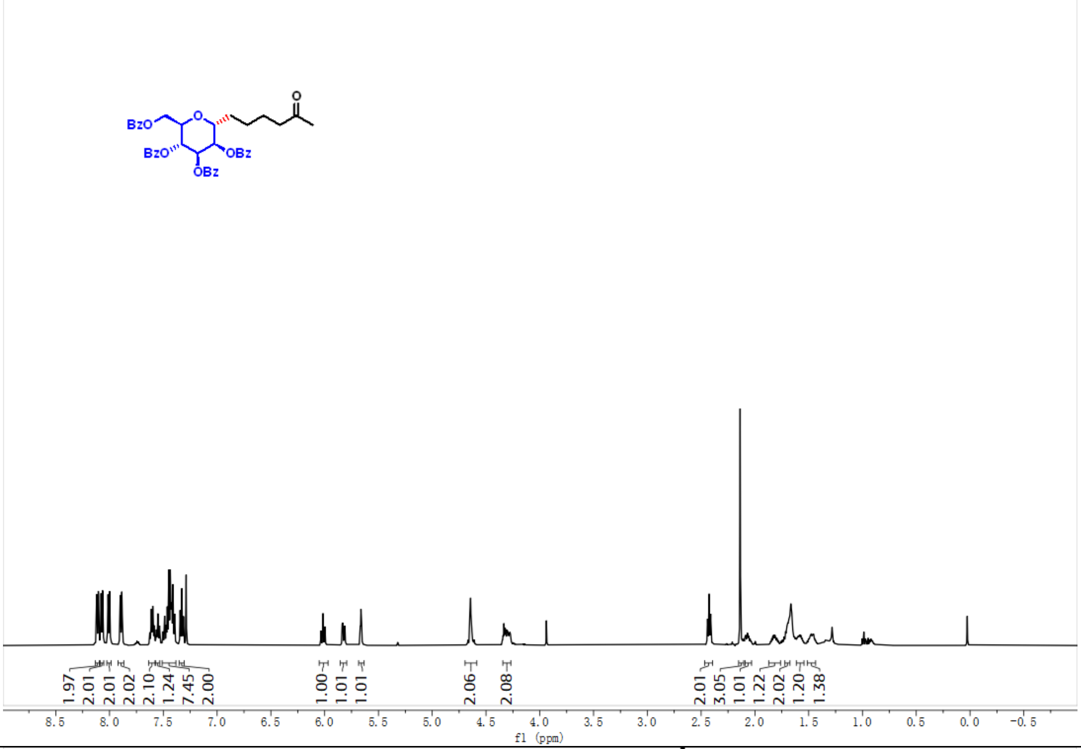

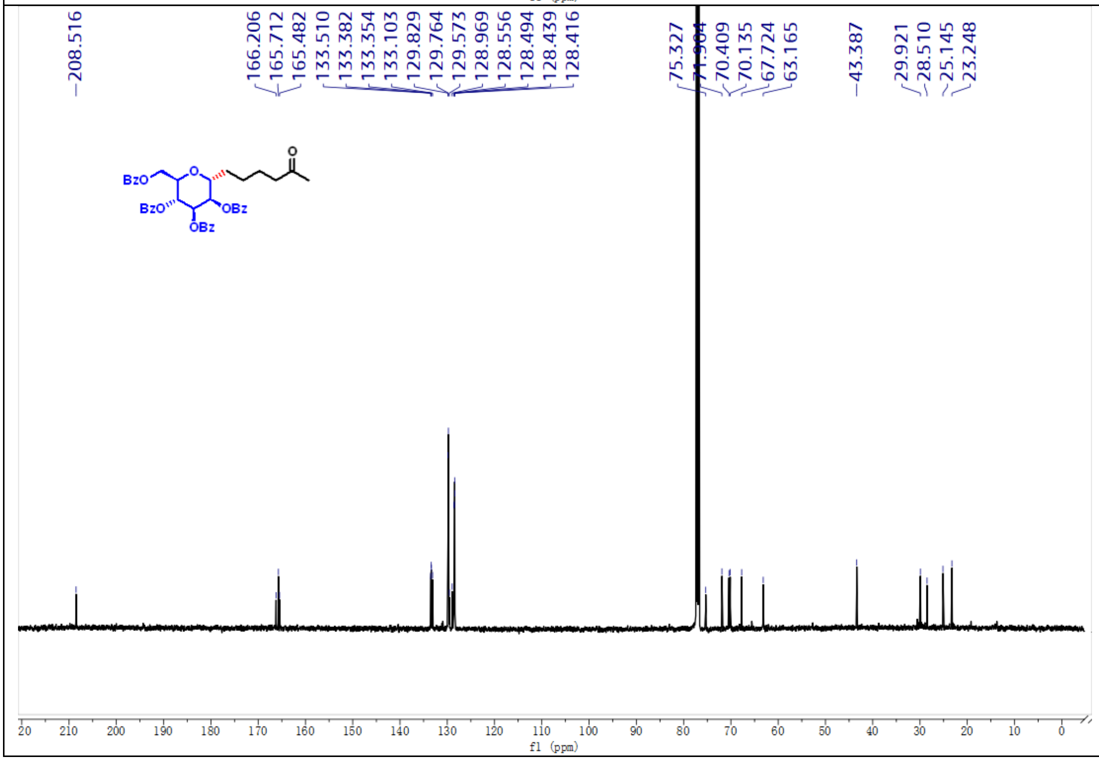

(2*R*,3*R*,4*R*,5*R*,6*R*)-2-((benzoyloxy)methyl)-6-(6-methoxy-6-oxohexyl)tetrahydro-2*H*-pyran-3,4,5-triyl tribenzoate (3aq)

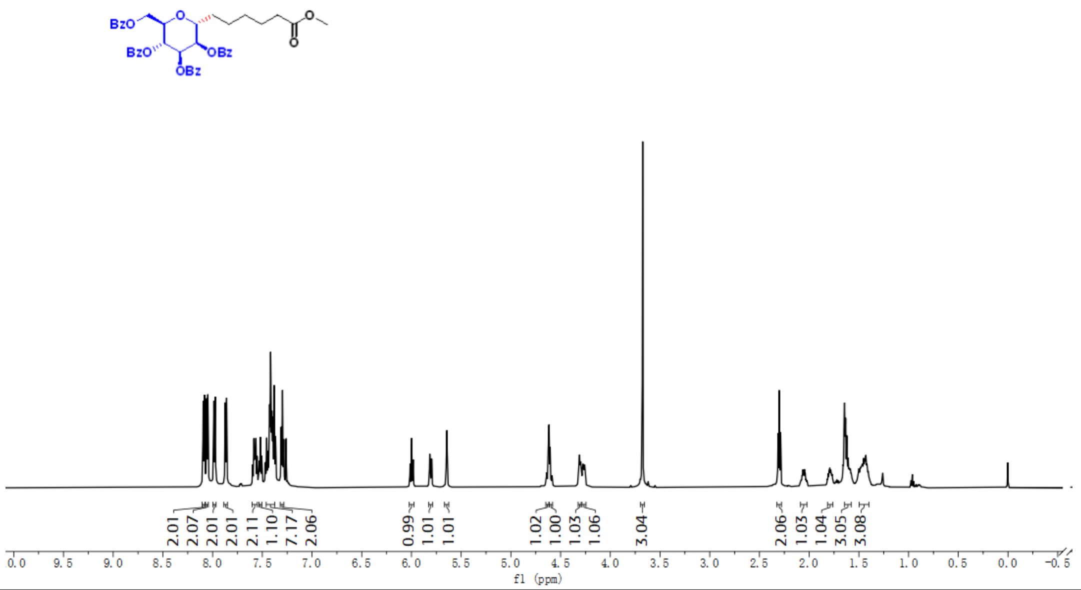

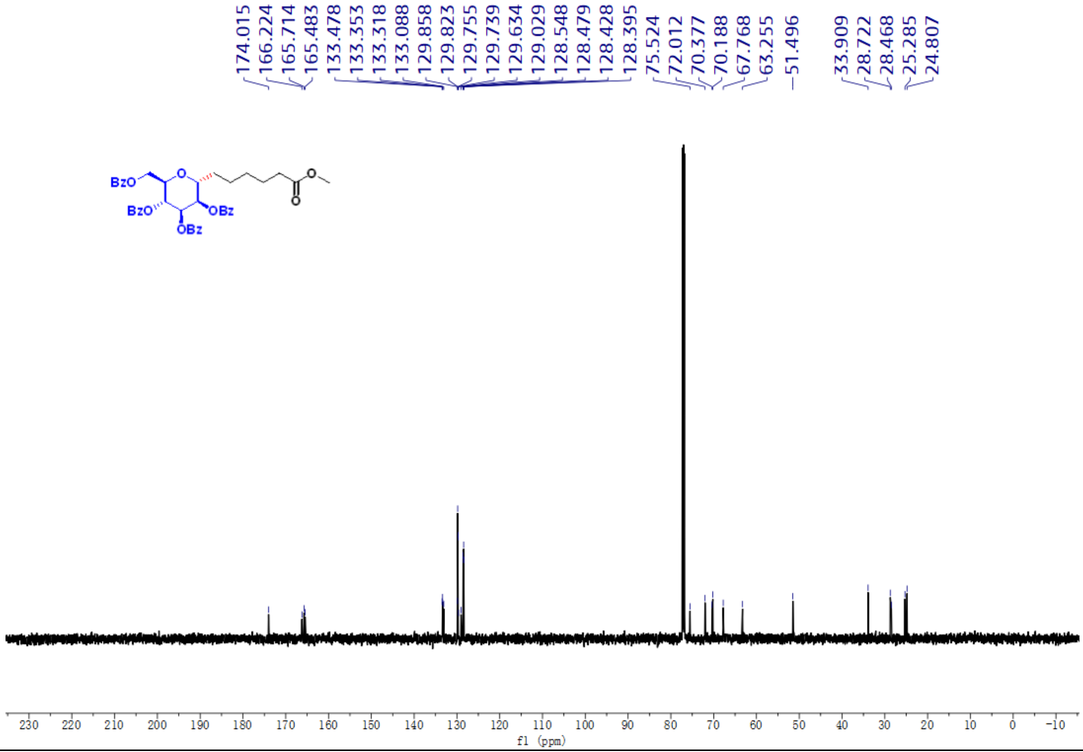

(2*R*,3*R*,4*R*,5*R*,6*R*)-2-((benzoyloxy)methyl)-6-(3-((methoxycarbonyl)oxy)propyl)tetrahydro-2*H*-pyran-3,4,5-triyl tribenzoate (3ar)

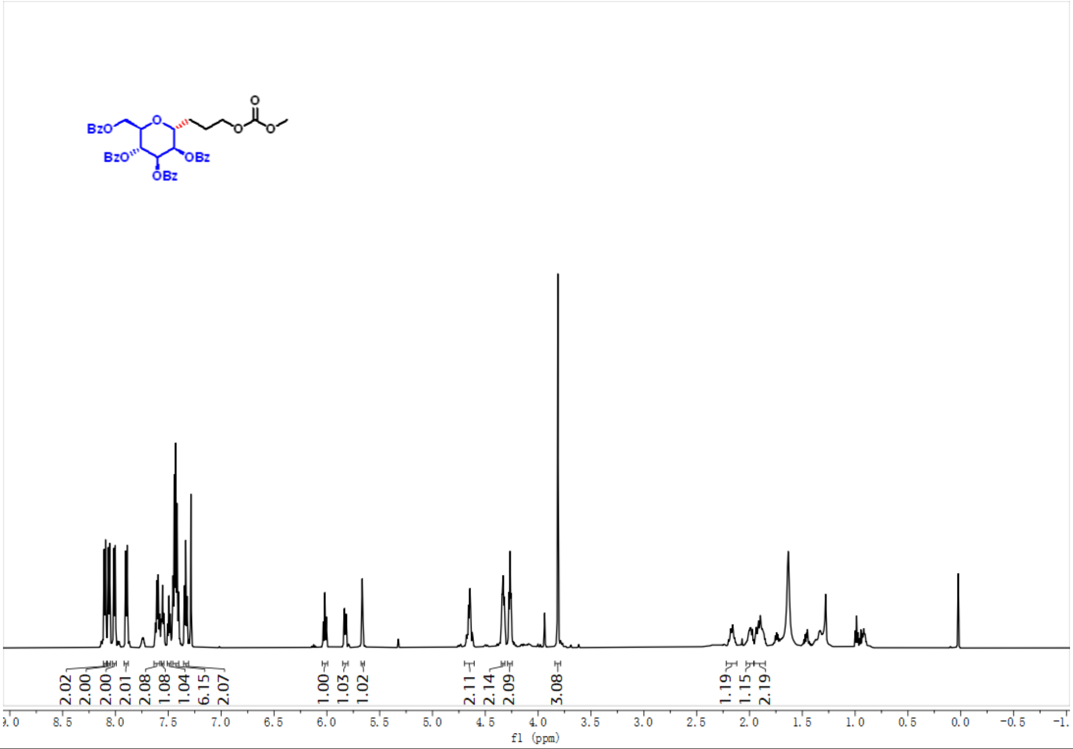

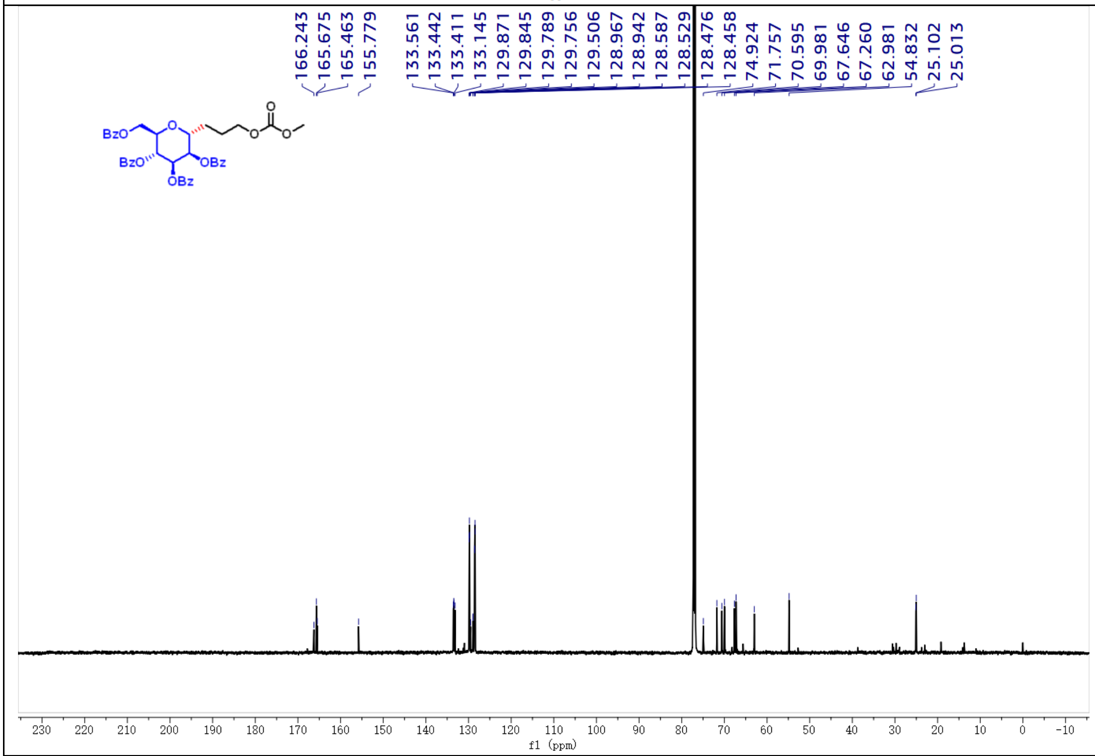

(2*R*,3*R*,4*R*,5*R*,6*R*)-2-((benzoyloxy)methyl)-6-(5-(4,4,5,5-tetramethyl-1,3,2-dioxaborolan-2-yl)pentyl)tetrahydro-2*H*-pyran-3,4,5-triyl tribenzoate (3as)

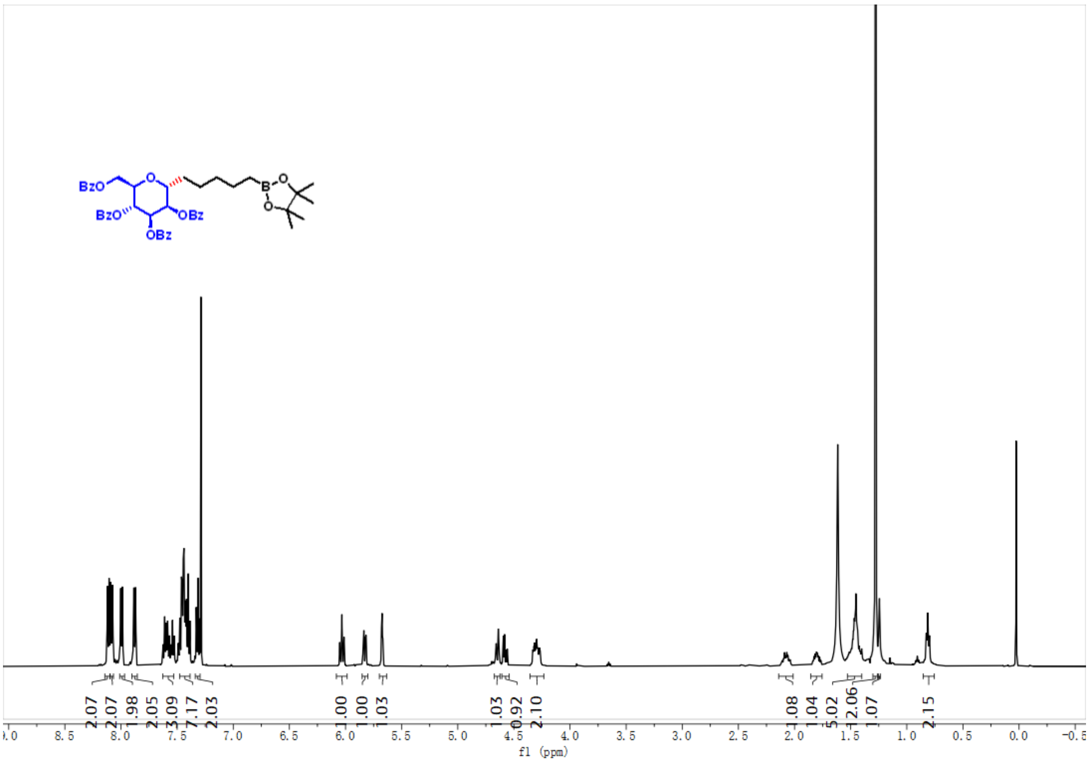

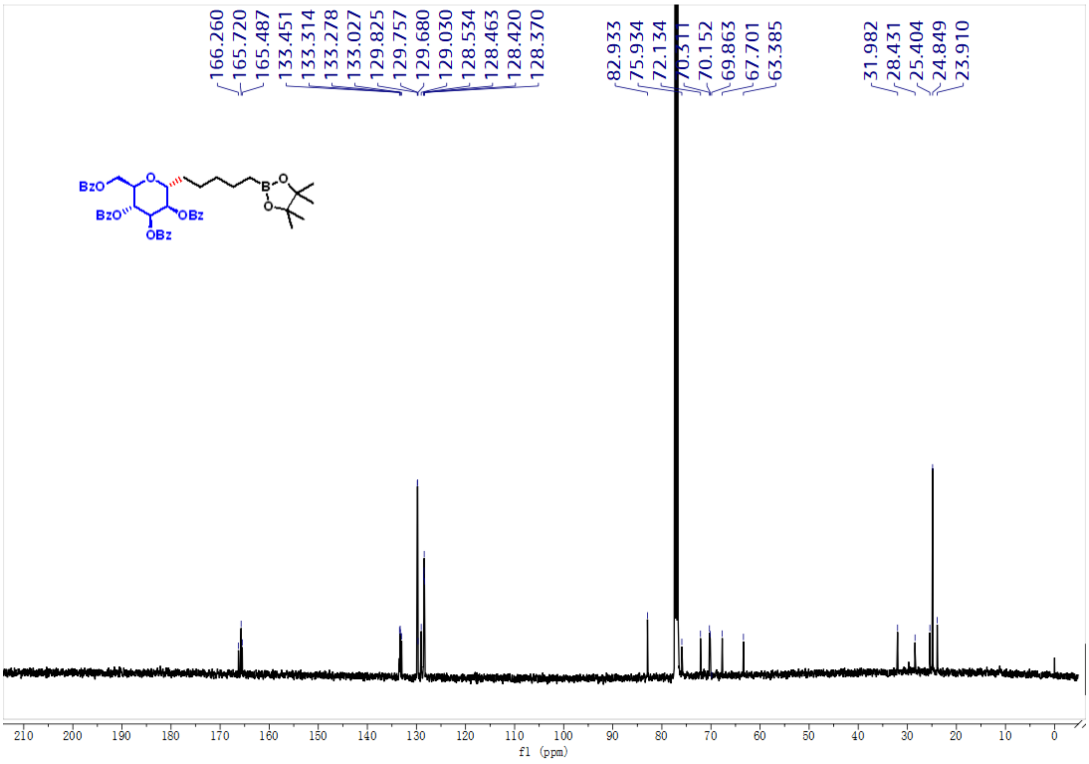

(2*R*,3*R*,4*R*,5*R*,6*R*)-2-((benzoyloxy)methyl)-6-(4-((*R*)-oxiran-2-yl)butyl)tetrahydro-2*H*-pyran-3,4,5-triyl tribenzoate (3at)

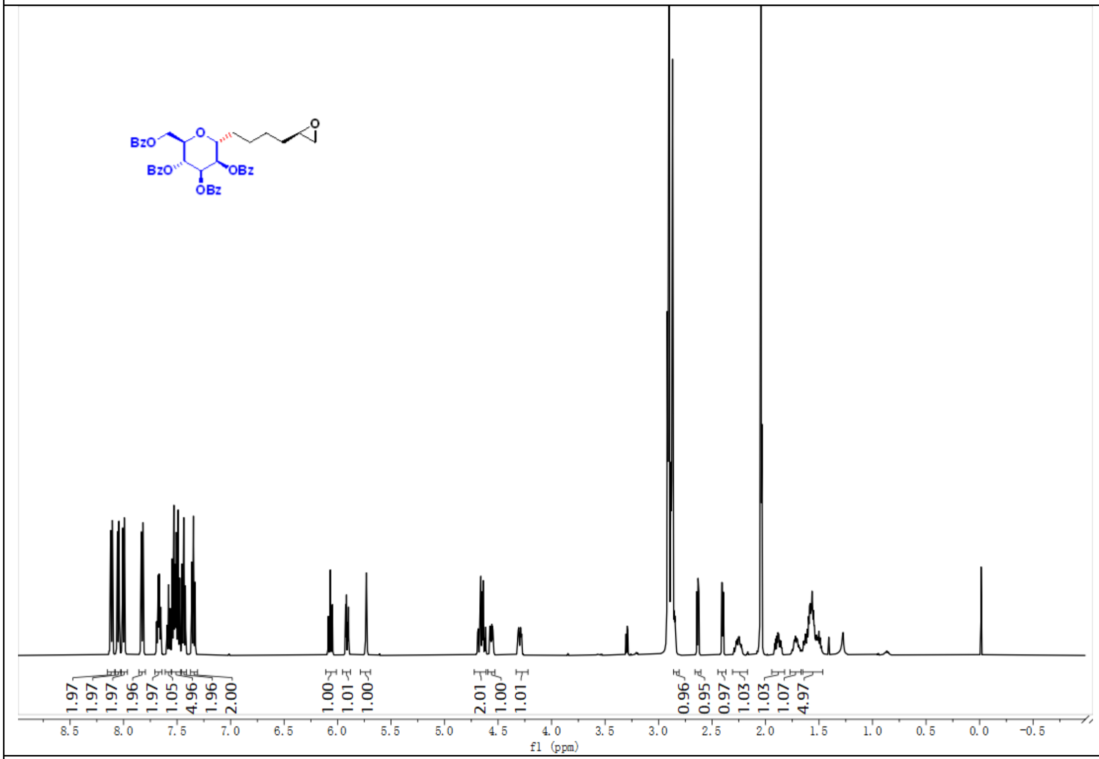

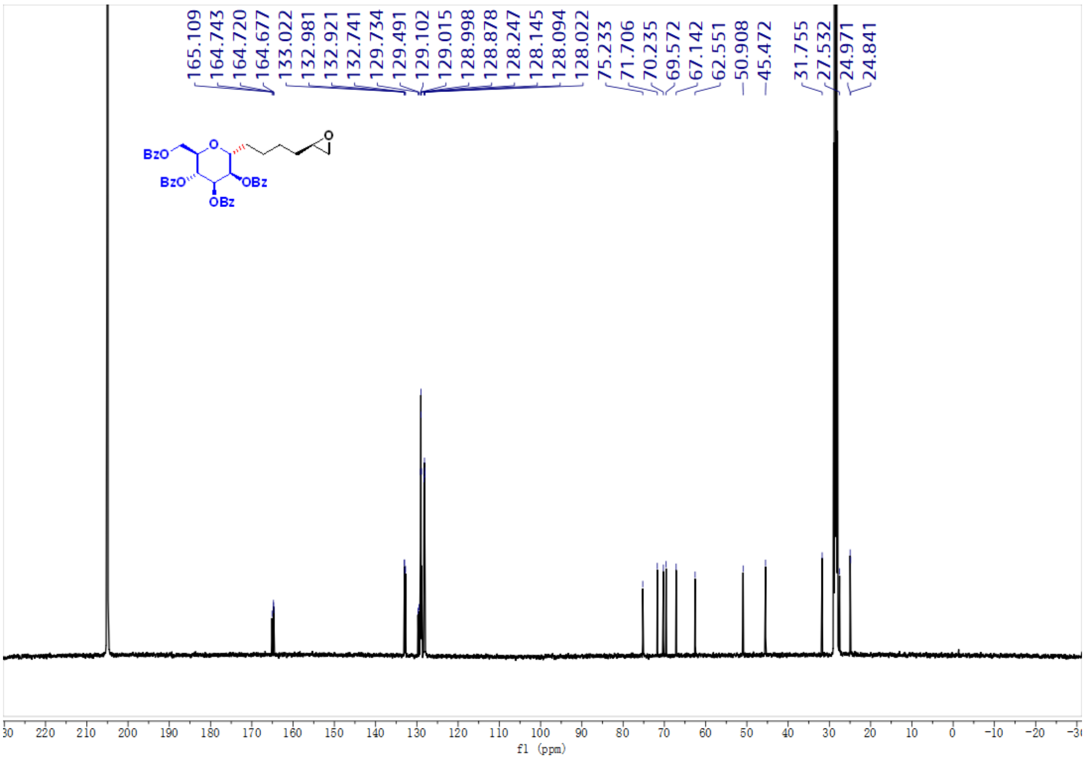

(2*R*,3*R*,4*R*,5*R*,6*R*)-2-((benzoyloxy)methyl)-6-(3-(trimethylsilyl)propyl)tetrahydro-2*H*-pyran-3,4,5-triyl tribenzoate (3au)

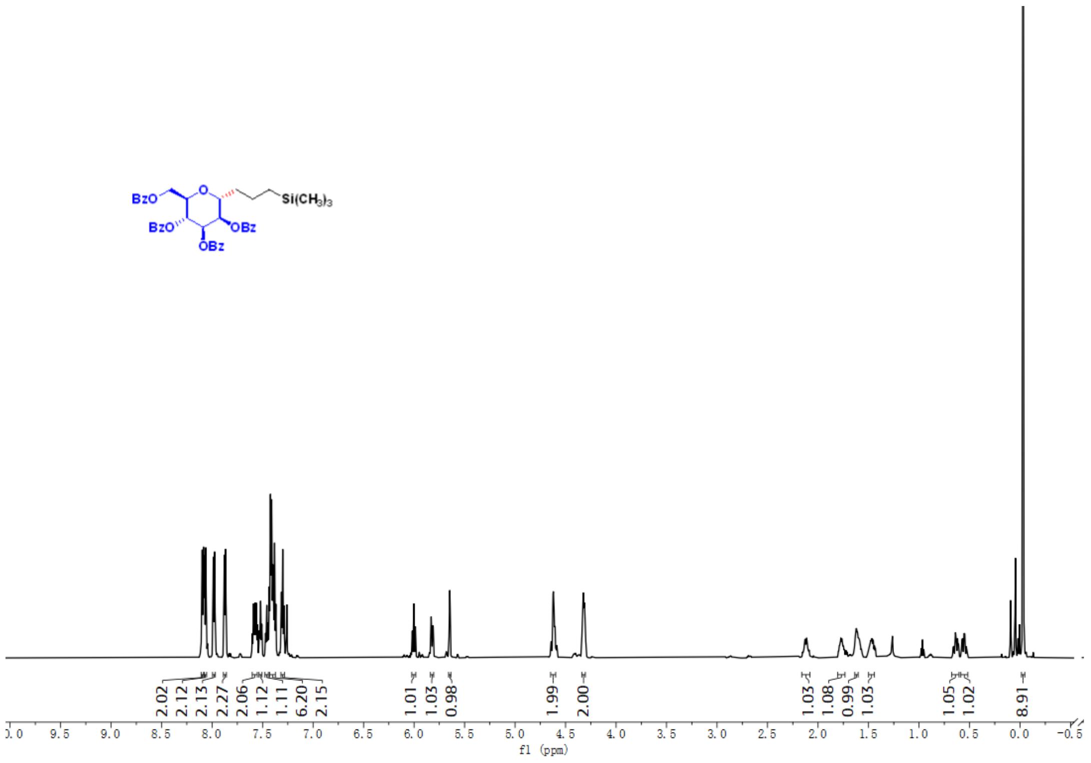

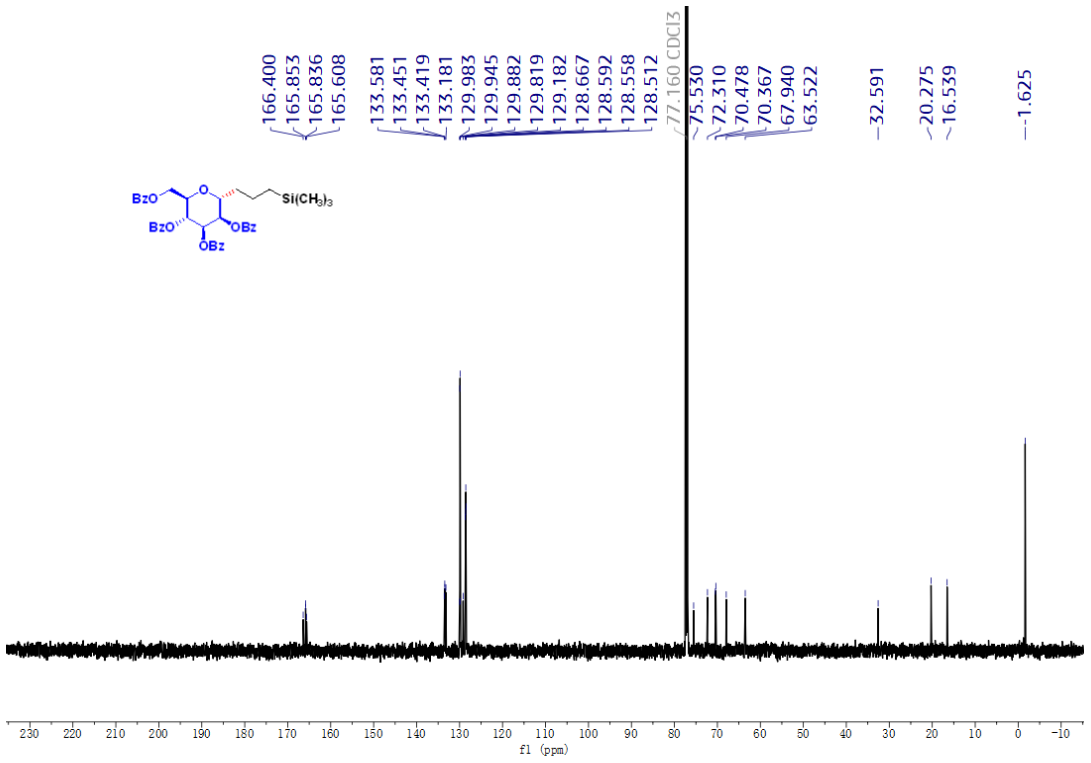

(2*R*,3*R*,4*R*,5*R*,6*R*)-2-((benzoyloxy)methyl)-6-(6-hydroxyhexyl)tetrahydro-2*H*-pyran-3,4,5-triyl tribenzoate (3av)

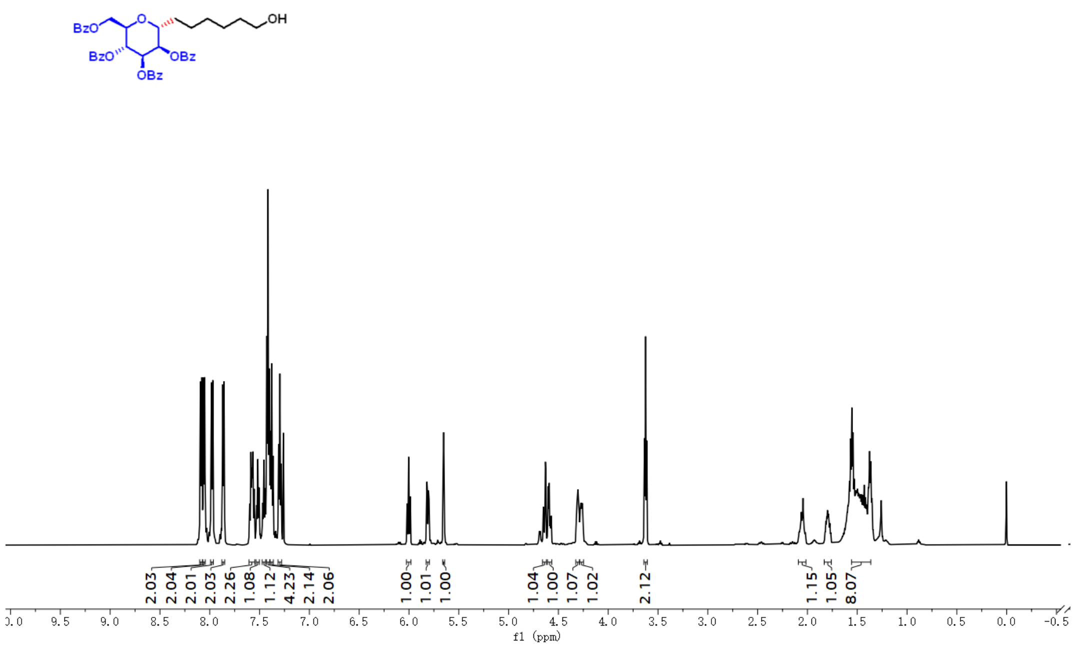

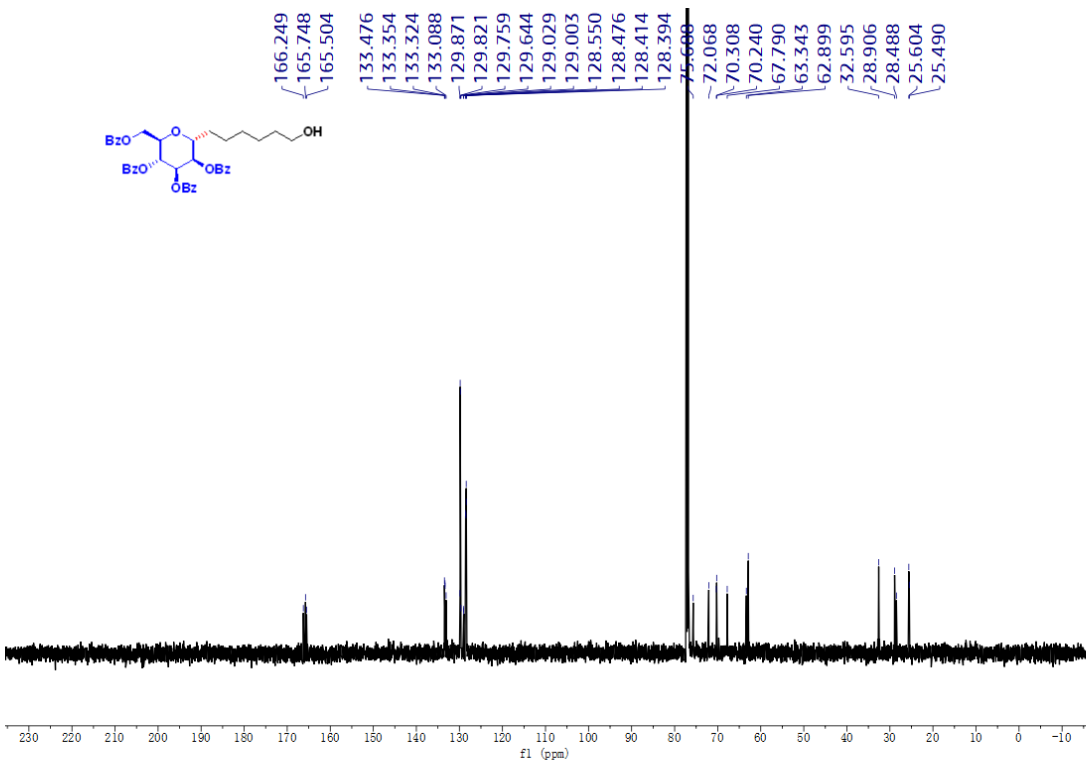

(2*R*,3*R*,4*R*,5*R*,6*R*)-2-((benzoyloxy)methyl)-6-((*S*)-4-((tert-

butoxycarbonyl)amino)-5-methoxy-5-oxopentyl)tetrahydro-2*H*-pyran-3,4,5-triyl tribenzoate (3aw)

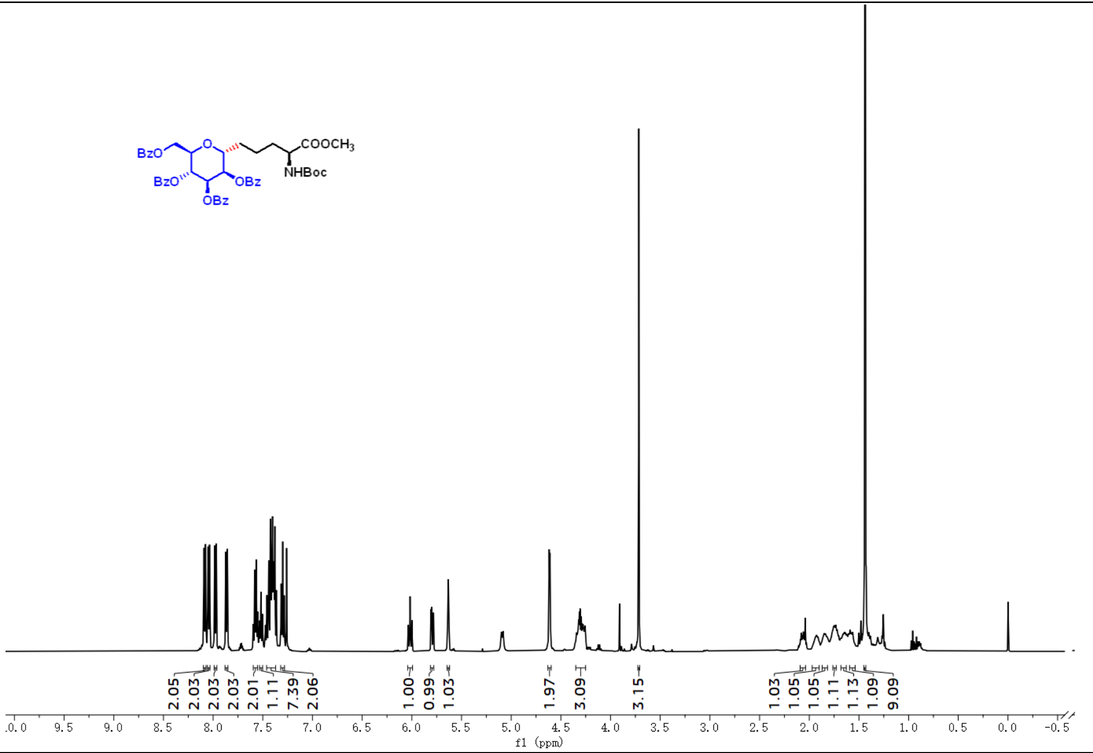

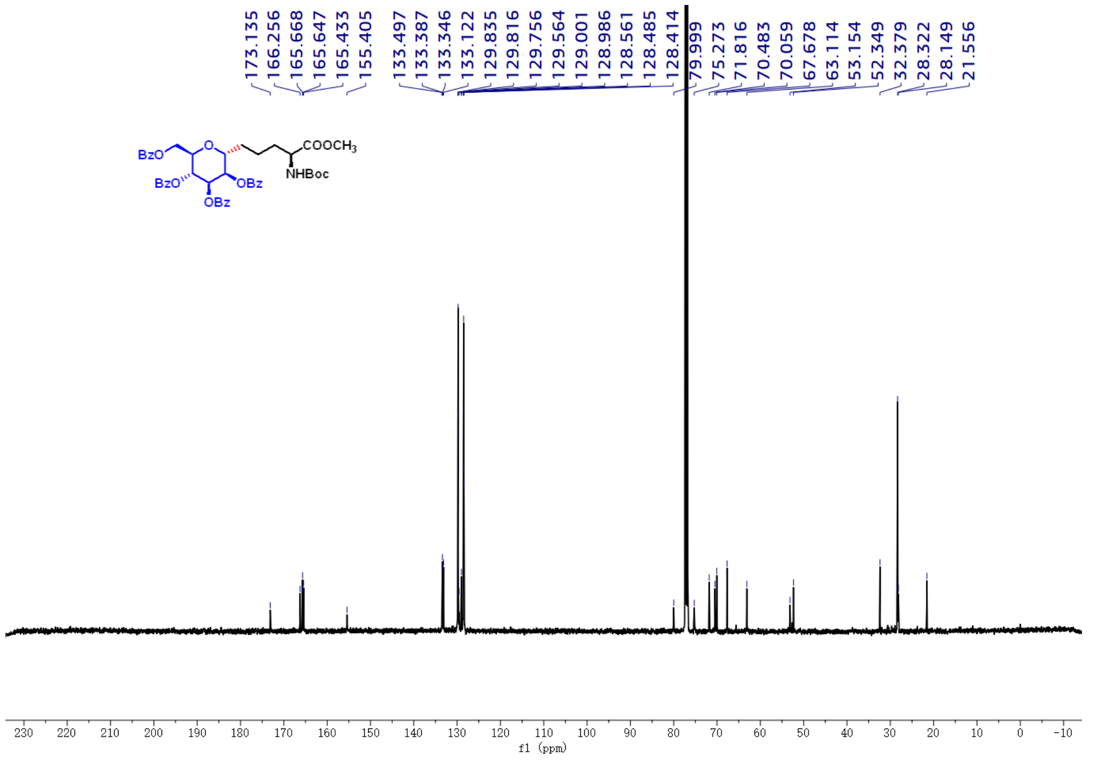

(2*R*,3*R*,4*R*,5*R*,6*R*)-2-((benzoyloxy)methyl)-6-cyclohexyltetrahydro-2*H*-pyran-3,4,5-triyl tribenzoate (3ax)

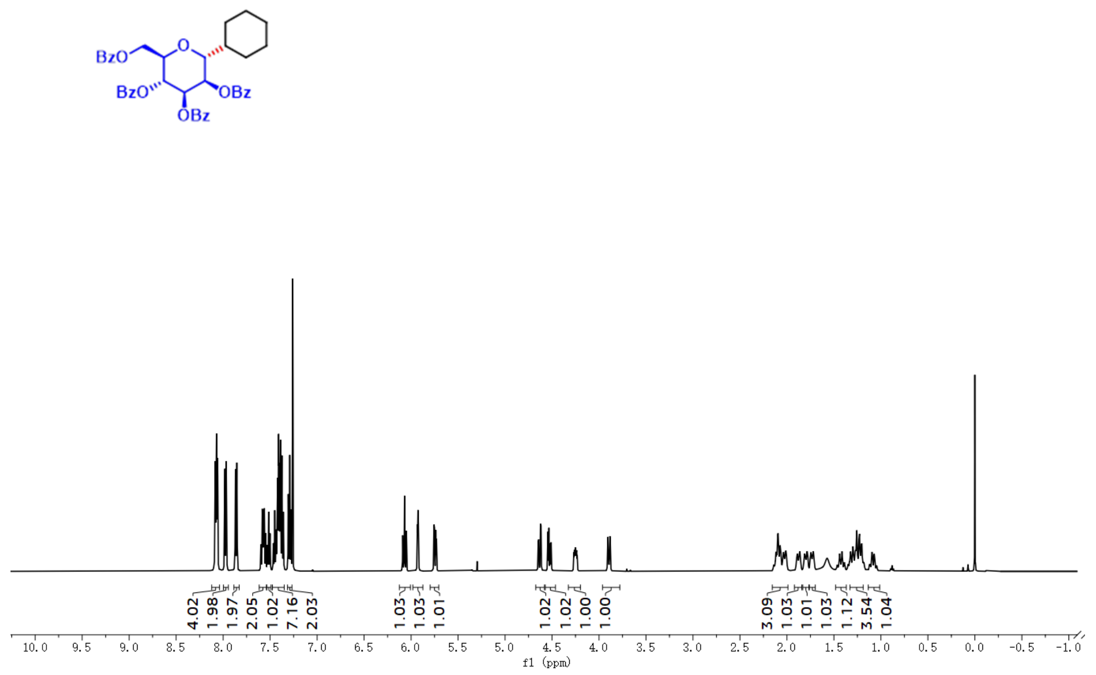

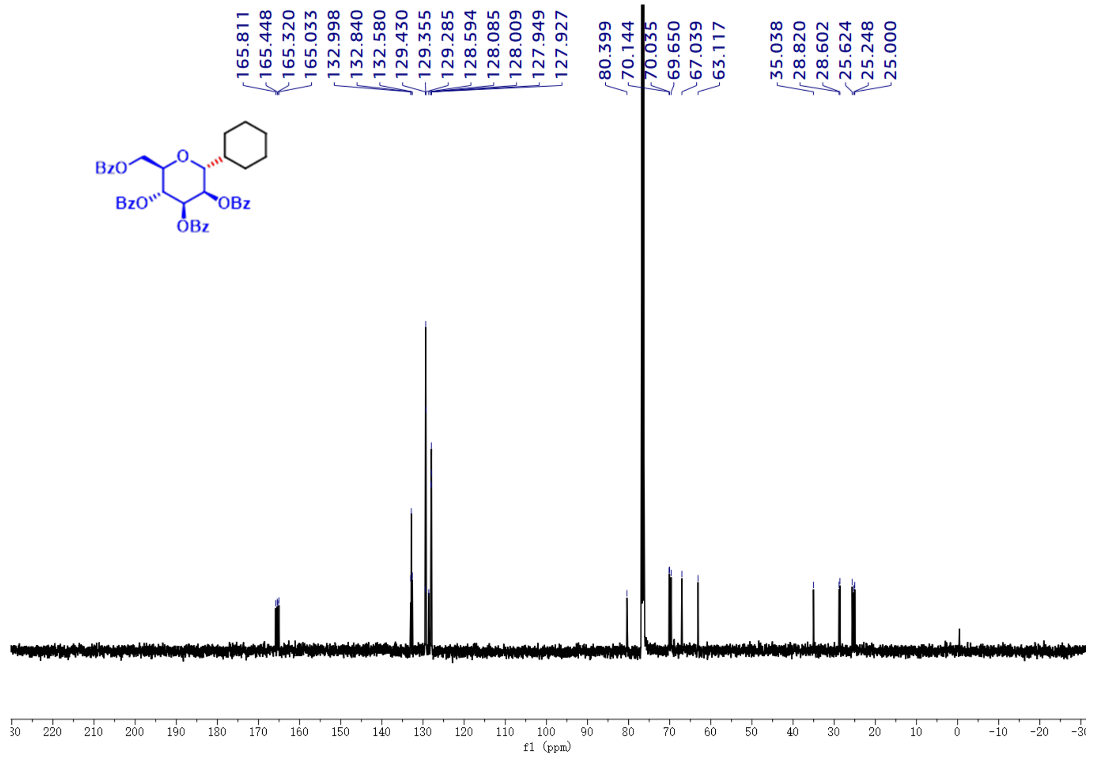

(2*R*,3*R*,4*R*,5*R*,6*R*)-2-(5-(4-acetamidophenoxy)pentyl)-6-(acetoxymethyl)tetrahydro-2*H*-pyran-3,4,5-triyl triacetate (3bn)

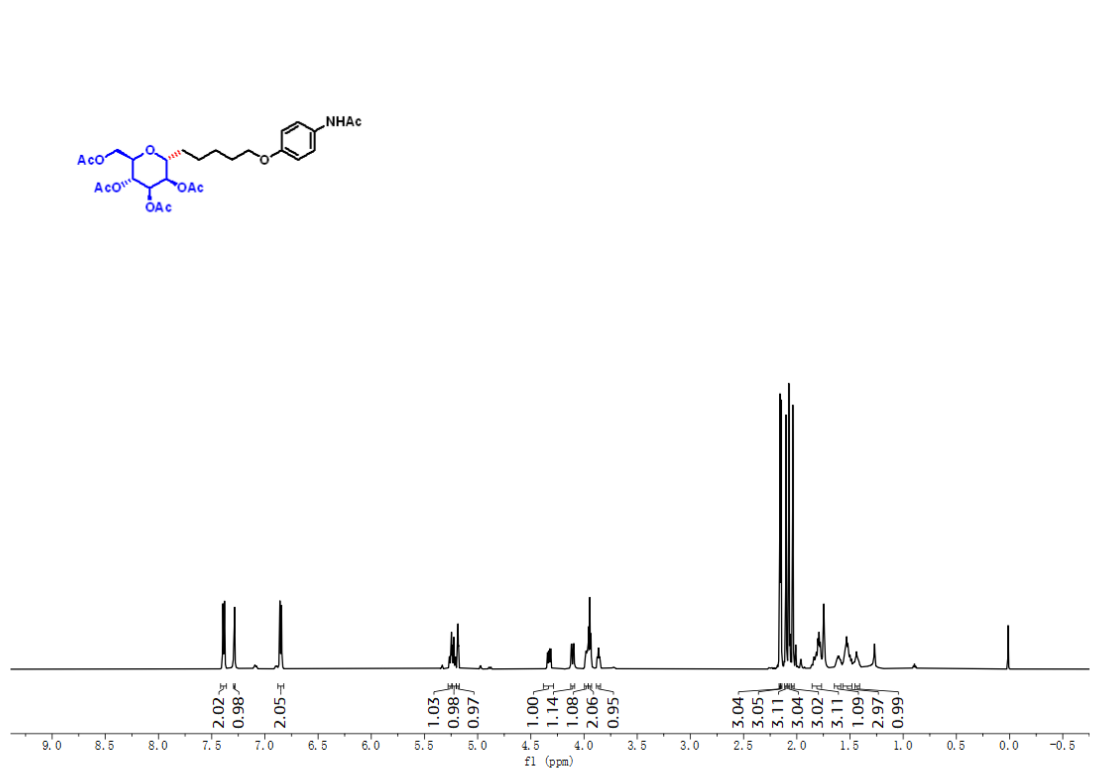

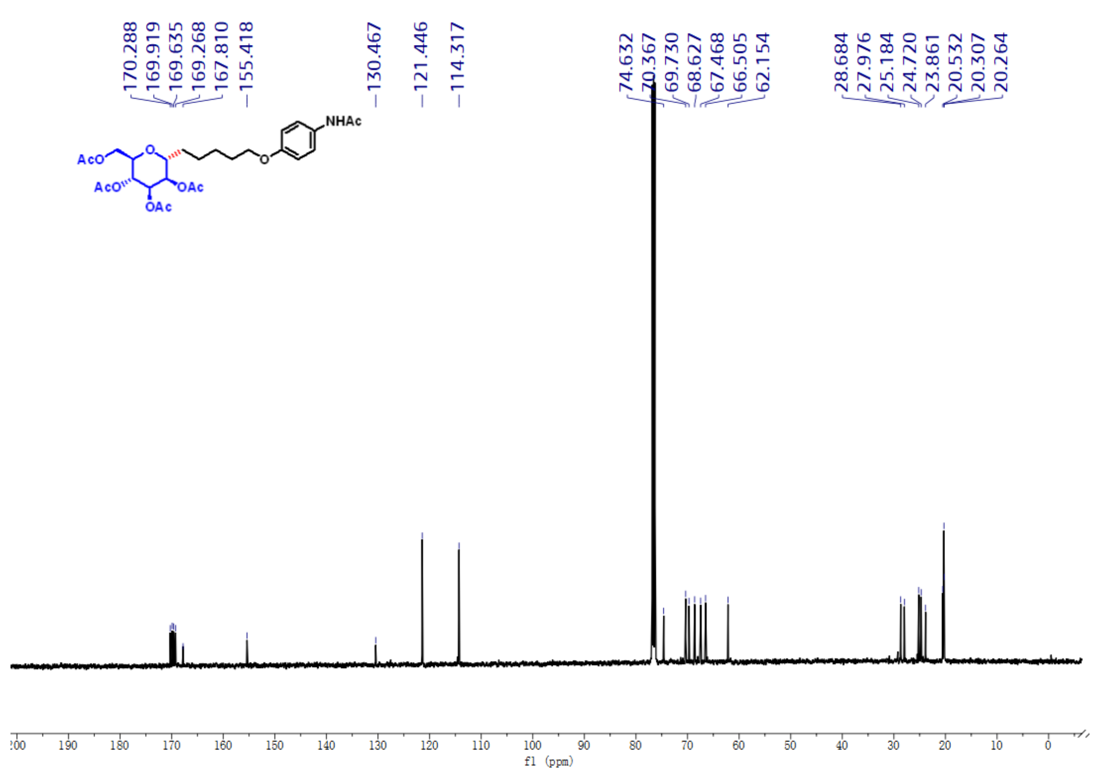

(2*R*,3*R*,4*R*,5*R*,6*R*)-2-(5-(4-acetamidophenoxy)pentyl)-6-((pivaloyloxy)methyl)tetrahydro-2*H*-pyran-3,4,5-triyl tris(2,2-dimethylpropanoate) (3cn)

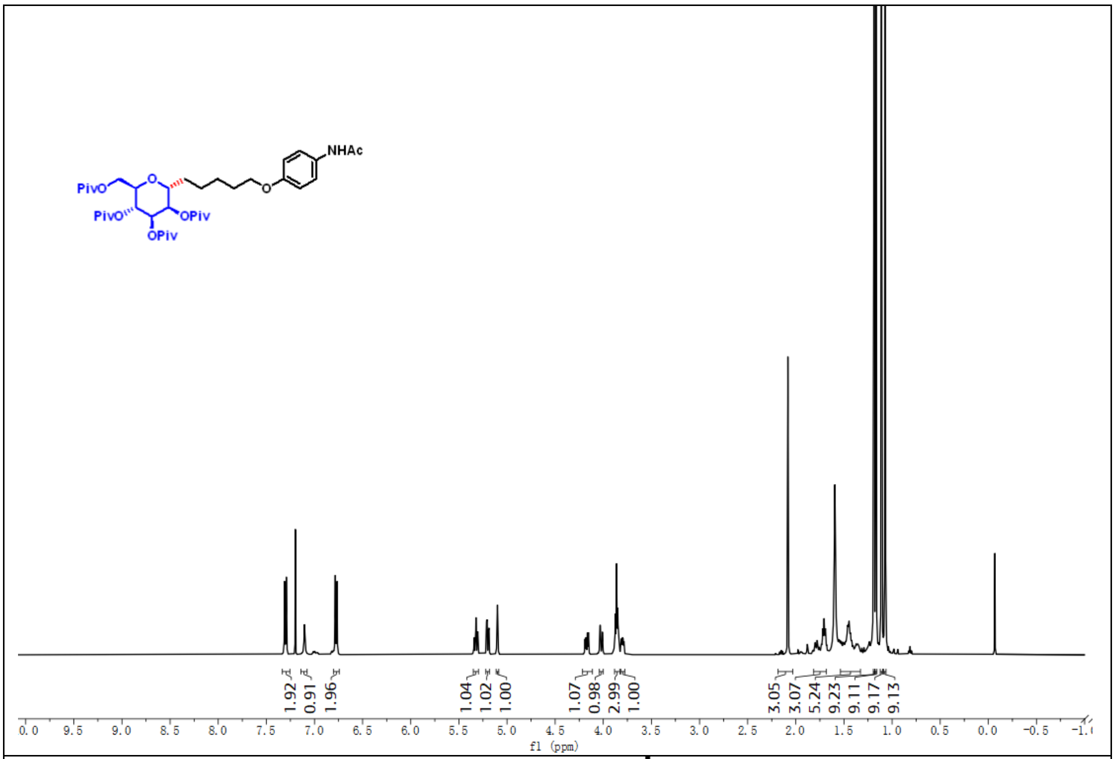

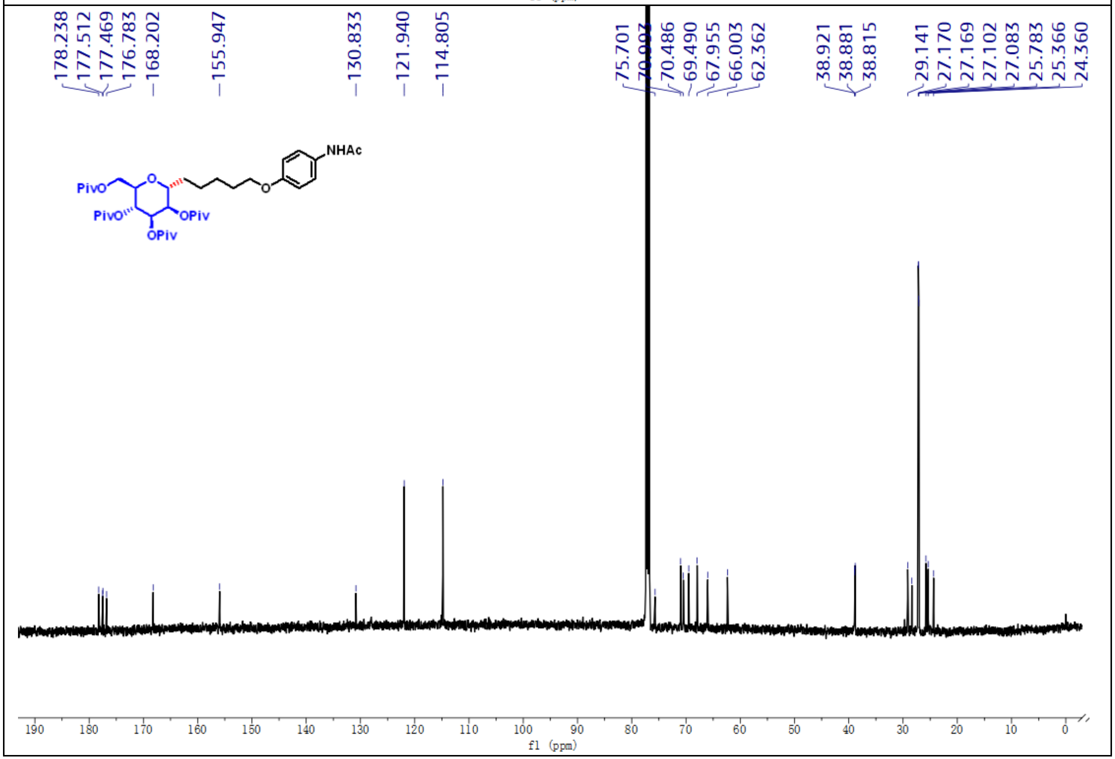

(2*R*,3*R*,4*R*,5*R*,6*R*)-2-(acetoxymethyl)-6-octyltetrahydro-2*H*-pyran-3,4,5-triyl tribenzoate (3da)

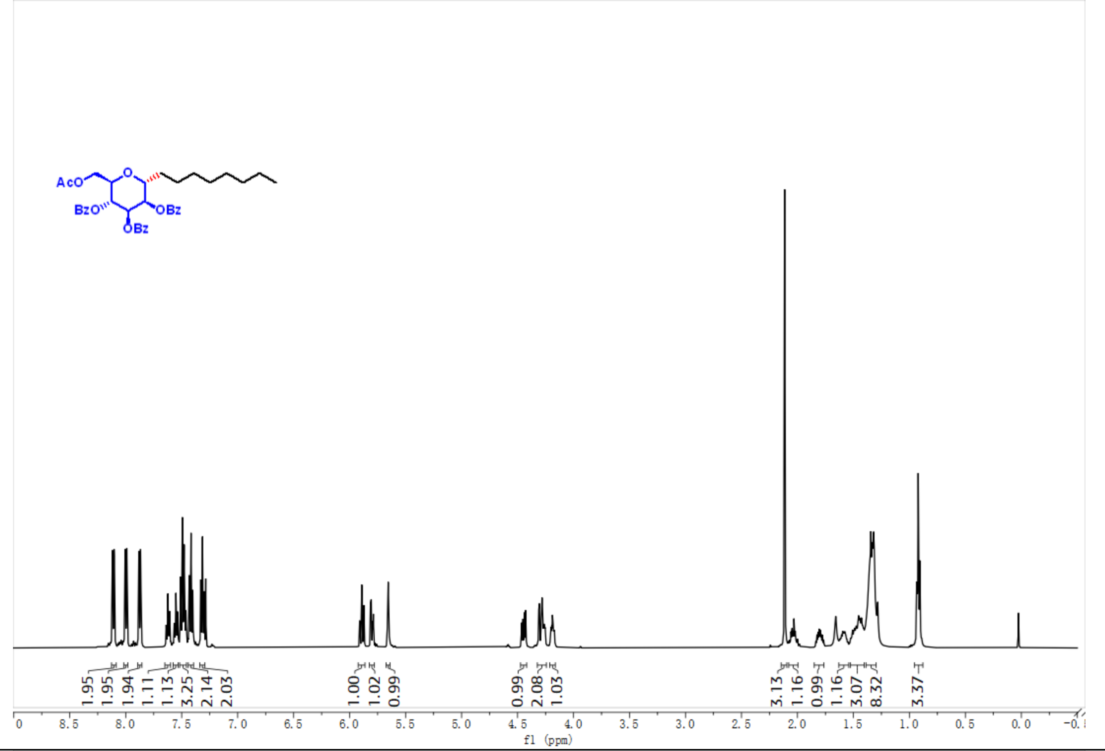

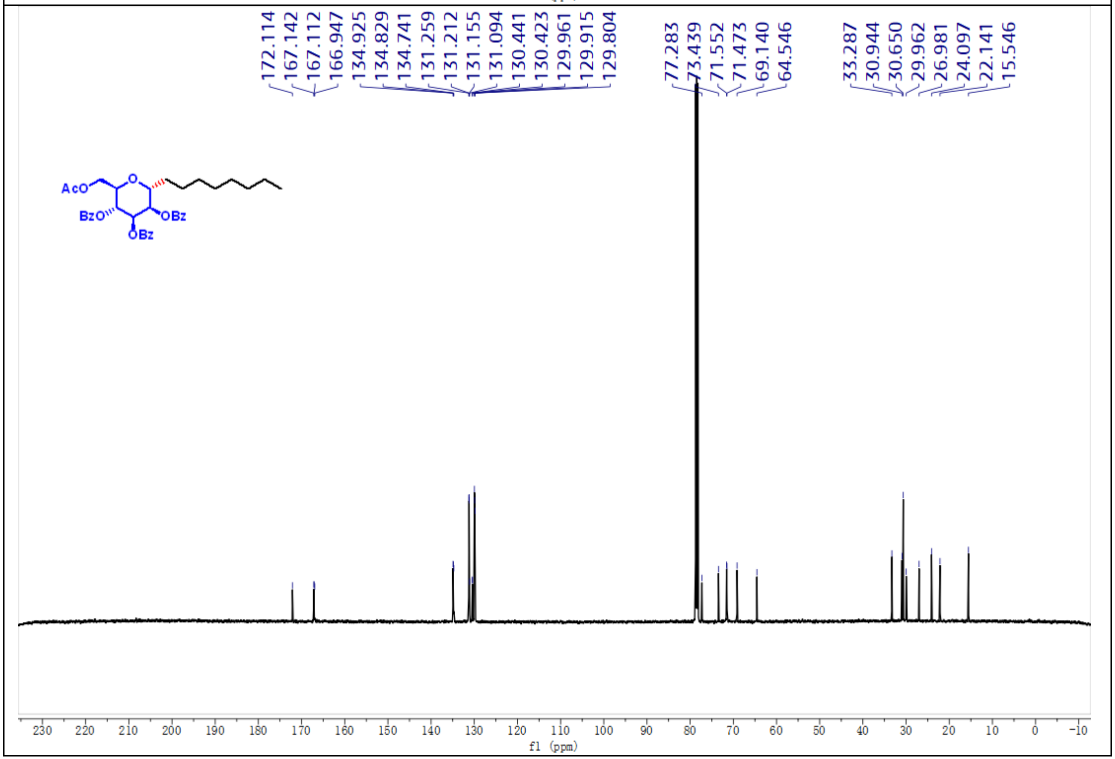

(2*R*,3*R*,4*R*,5*R*,6*R*)-2-(((4-methoxybenzoyl)oxy)methyl)-6-octyltetrahydro-2*H*-pyran-3,4,5-triyl tribenzoate (3ea)

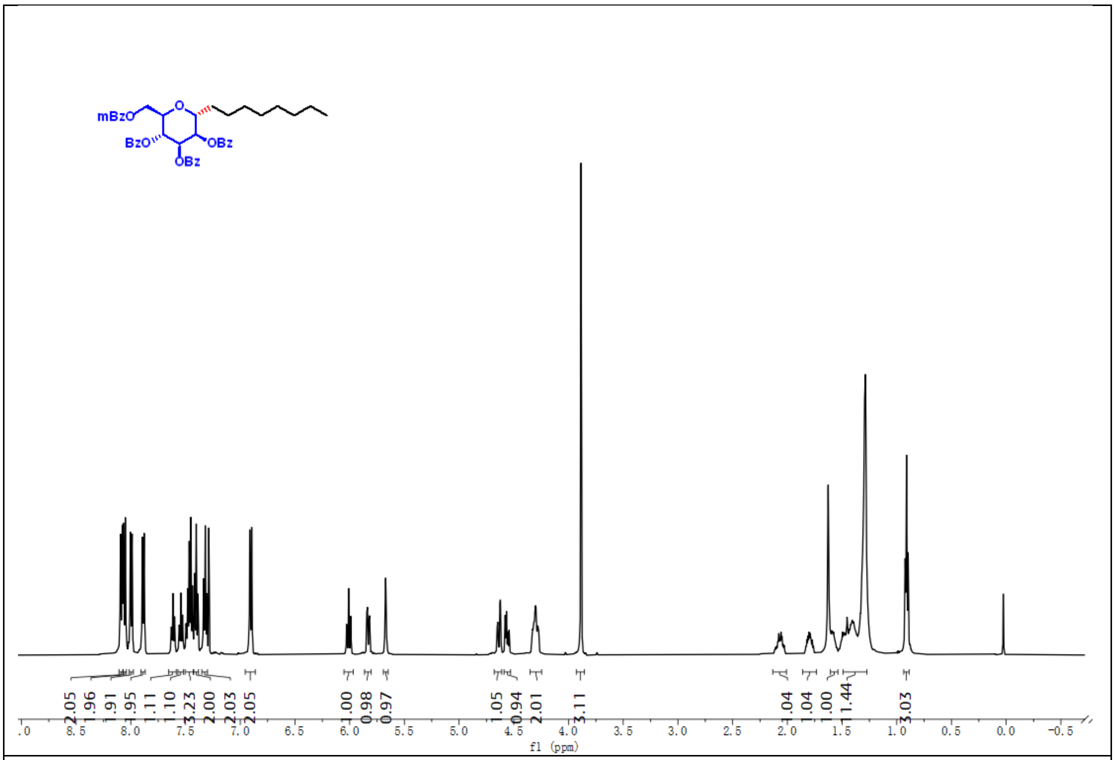

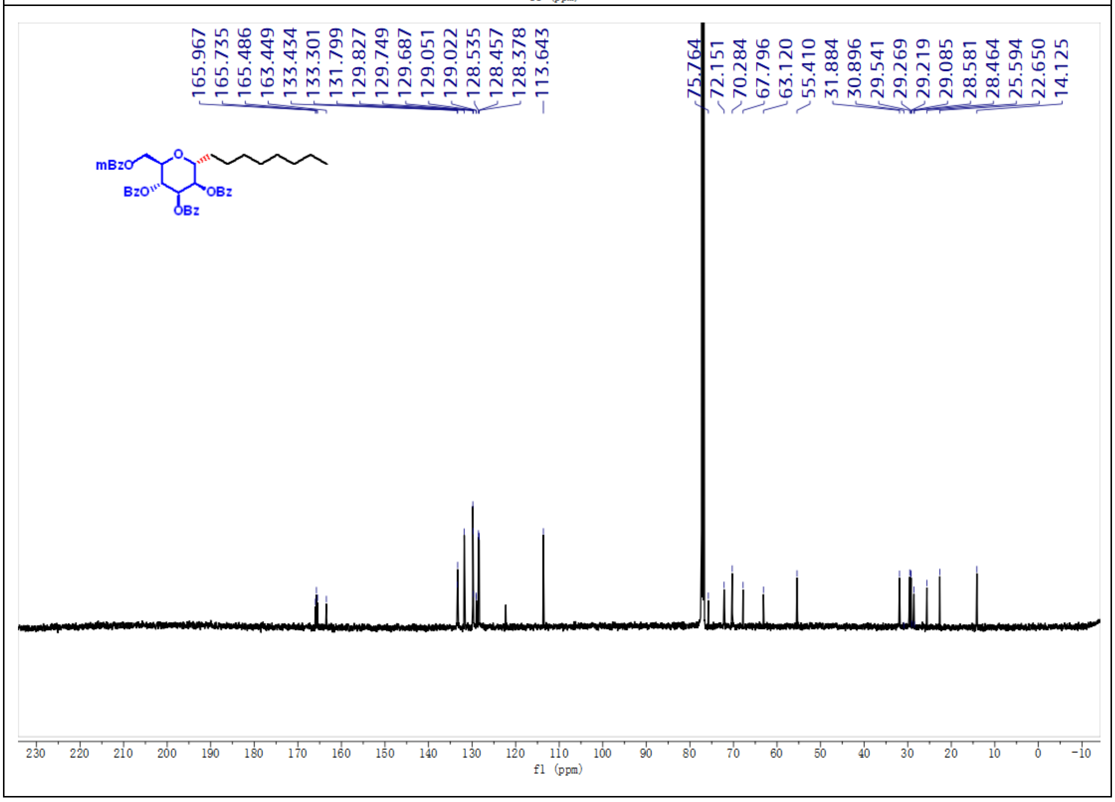

(2*S*,3*S*,4*R*,5*S*,6*S*)-2-methyl-6-octyltetrahydro-2*H*-pyran-3,4,5-triyl tribenzoate (3fa)

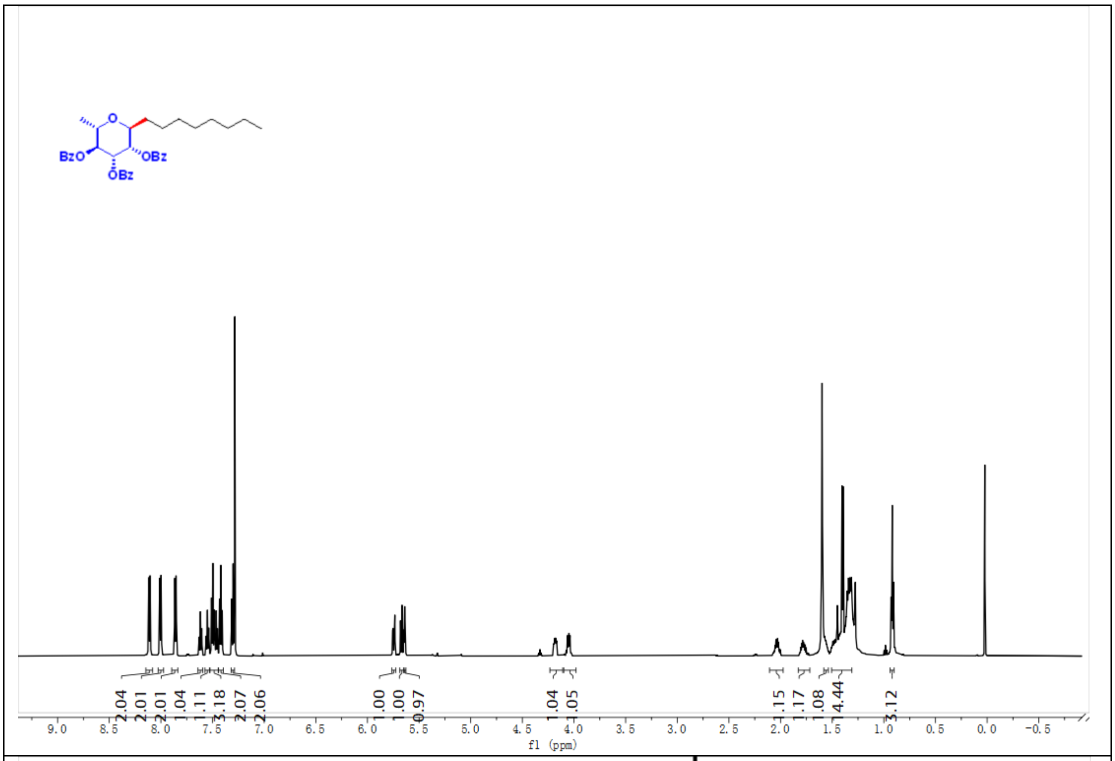

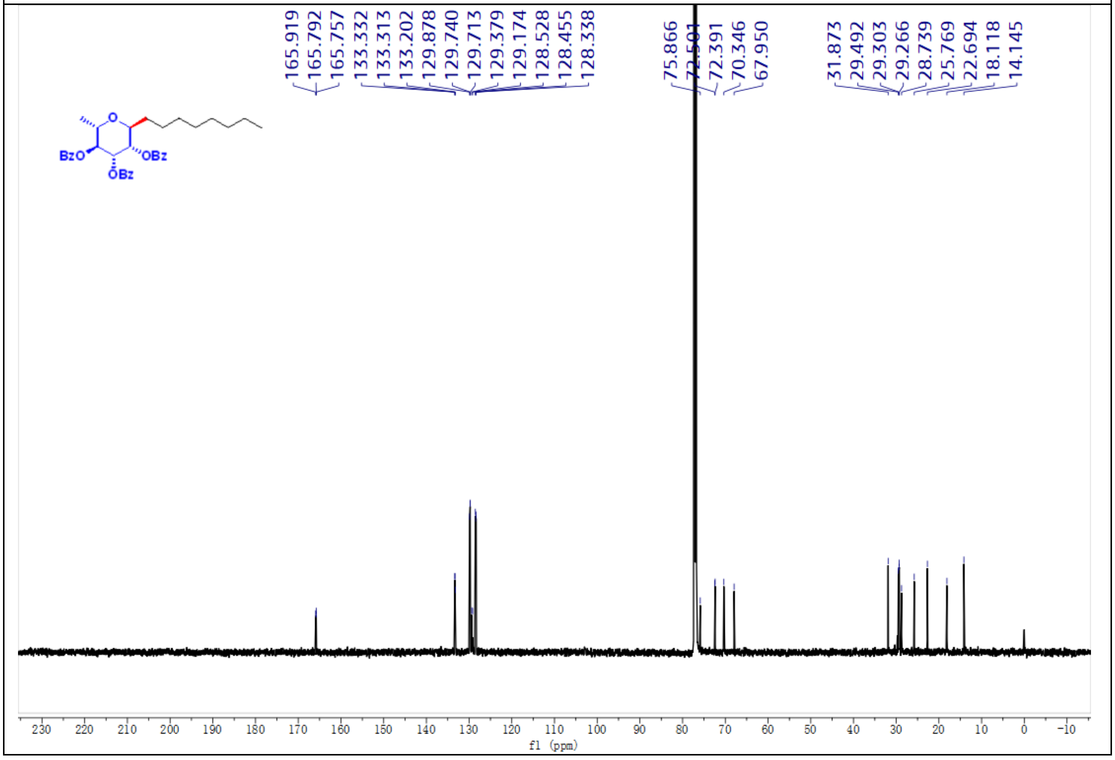

(2*S*,3*S*,4*R*,5*S*,6*S*)-2-(5-(4-acetamidophenoxy)pentyl)-6-methyltetrahydro-2*H*-pyran-3,4,5-triyl triacetate (3ga)

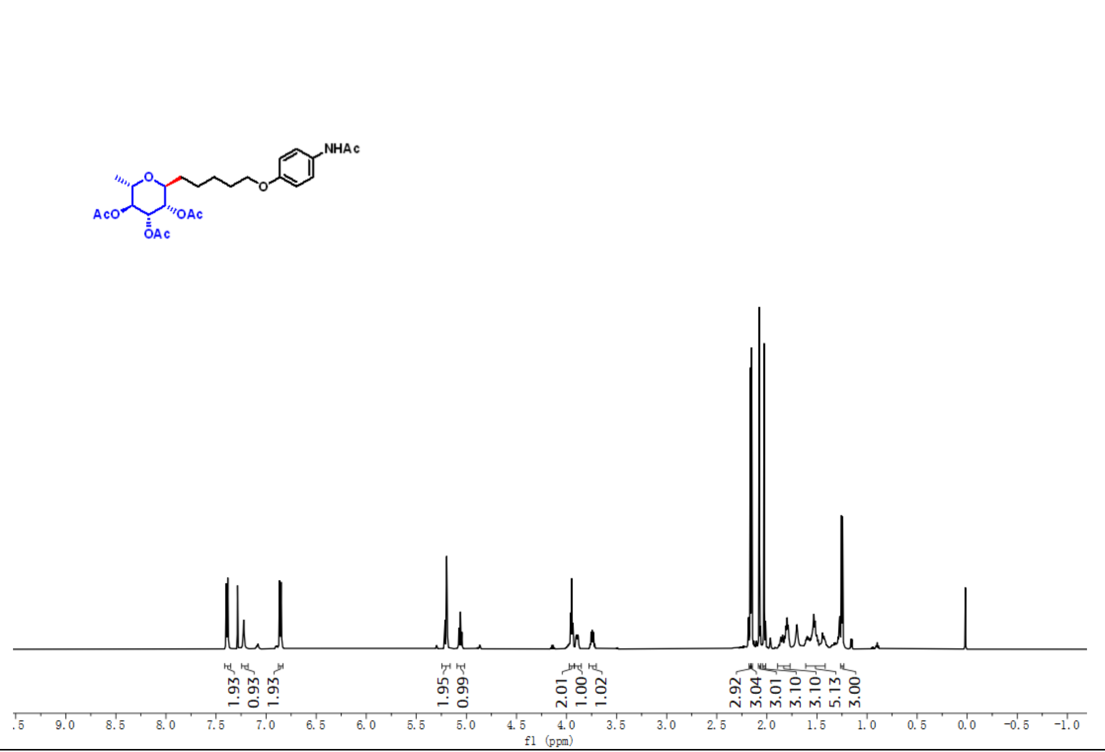

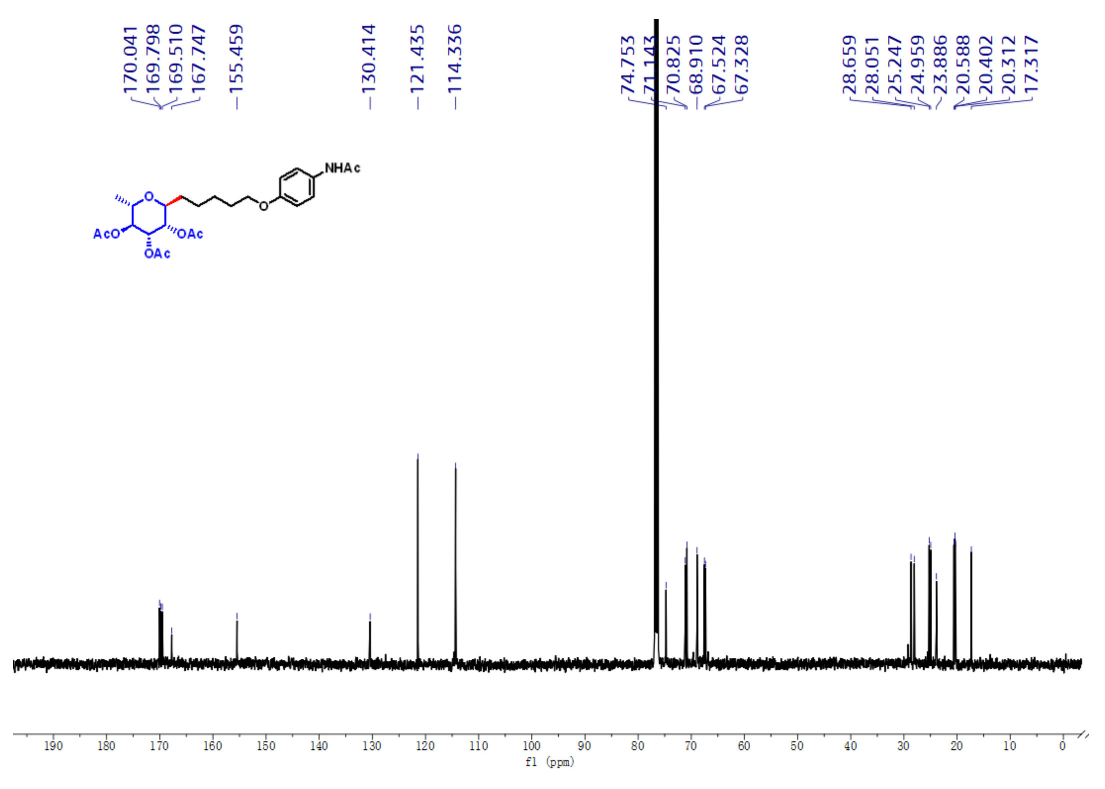

(2*S*,3*S*,4*R*,5*S*,6*S*)-2-(5-(4-acetamidophenoxy)pentyl)-6-methyltetrahydro-2*H*-pyran-3,4,5-triyl tris(2,2-dimethylpropanoate) (3hn)

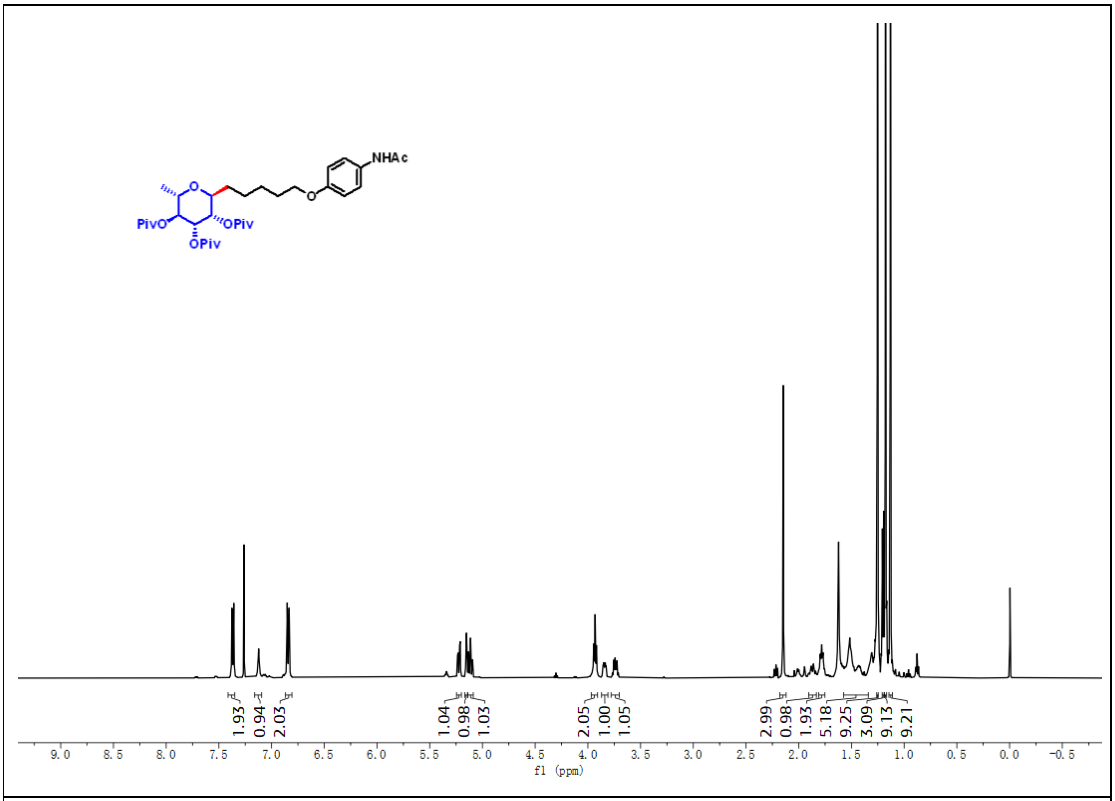

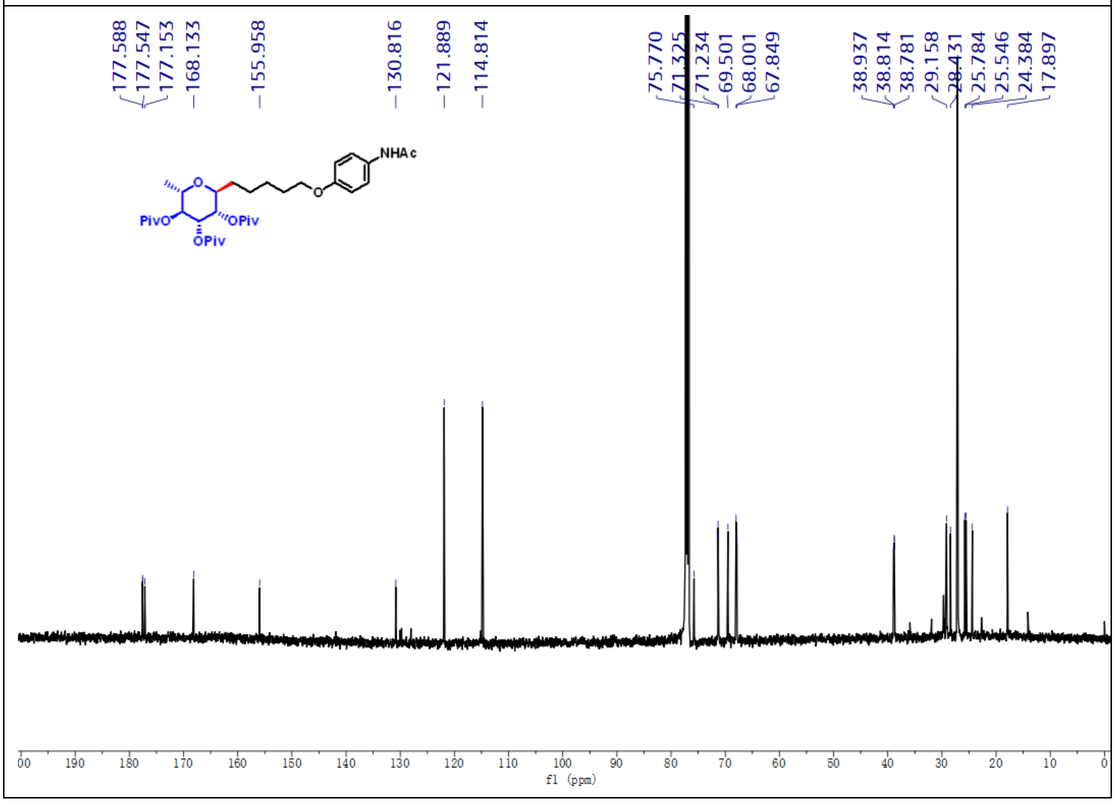

((2*R*,3*R*,4*S*,5*S*)-3-(benzoyloxy)-4-fluoro-5-octyltetrahydrofuran-2-yl)methyl benzoate (3ia)

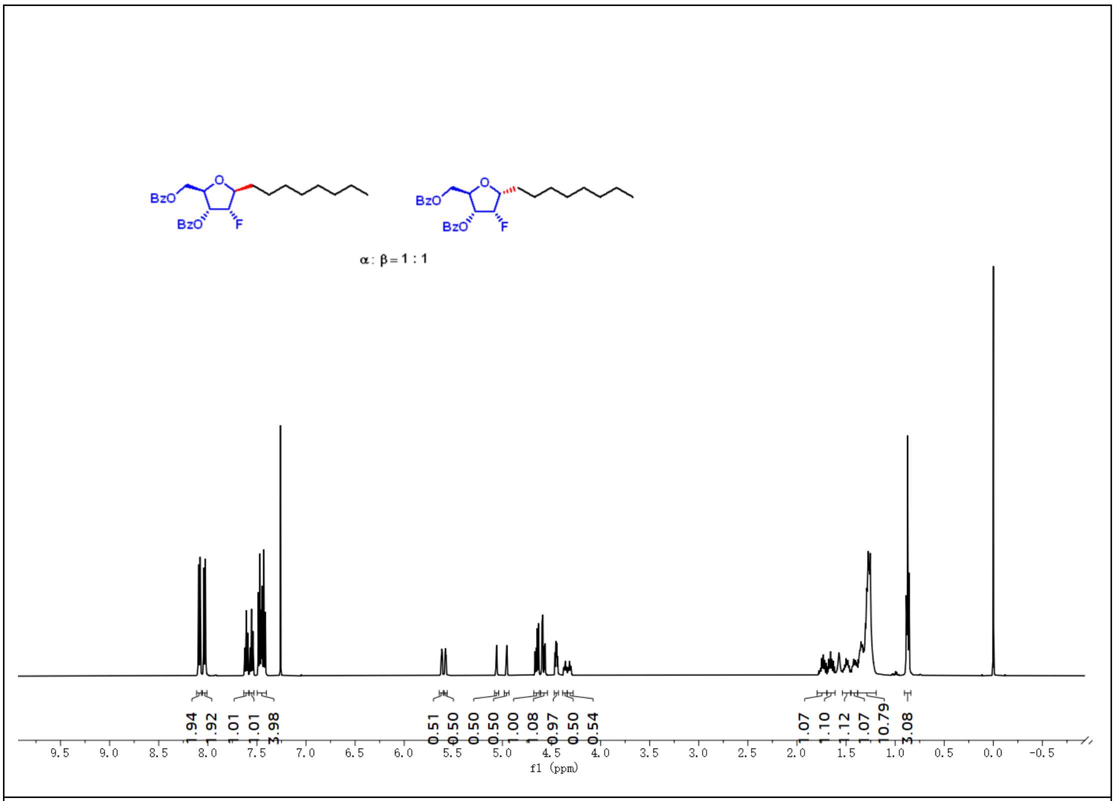

BzO
BzO
F
α : β = 1 : 1
1.94
1.92
1.01
1.01
3.98
0.51
0.50
0.50
0.50
1.00
1.08
0.97
0.50
0.54
1.07
1.10
1.12
1.07
10.79
3.08
f1 (ppm)

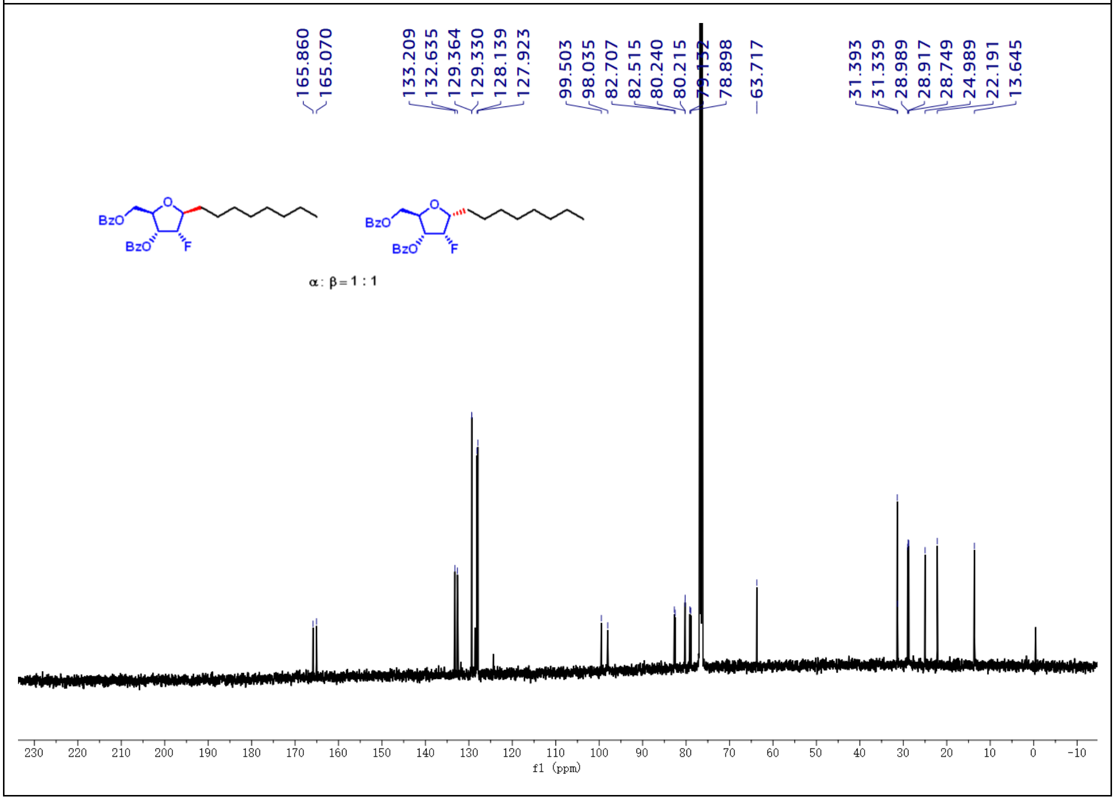

165.860
165.070
133.209
132.635
129.364
129.330
128.139
127.923
99.503
98.035
82.707
82.515
80.240
80.215
78.898
63.717
31.393
31.339
28.989
28.917
28.749
24.989
22.191
13.645
BzO
BzO
F
α : β = 1 : 1
f1 (ppm)

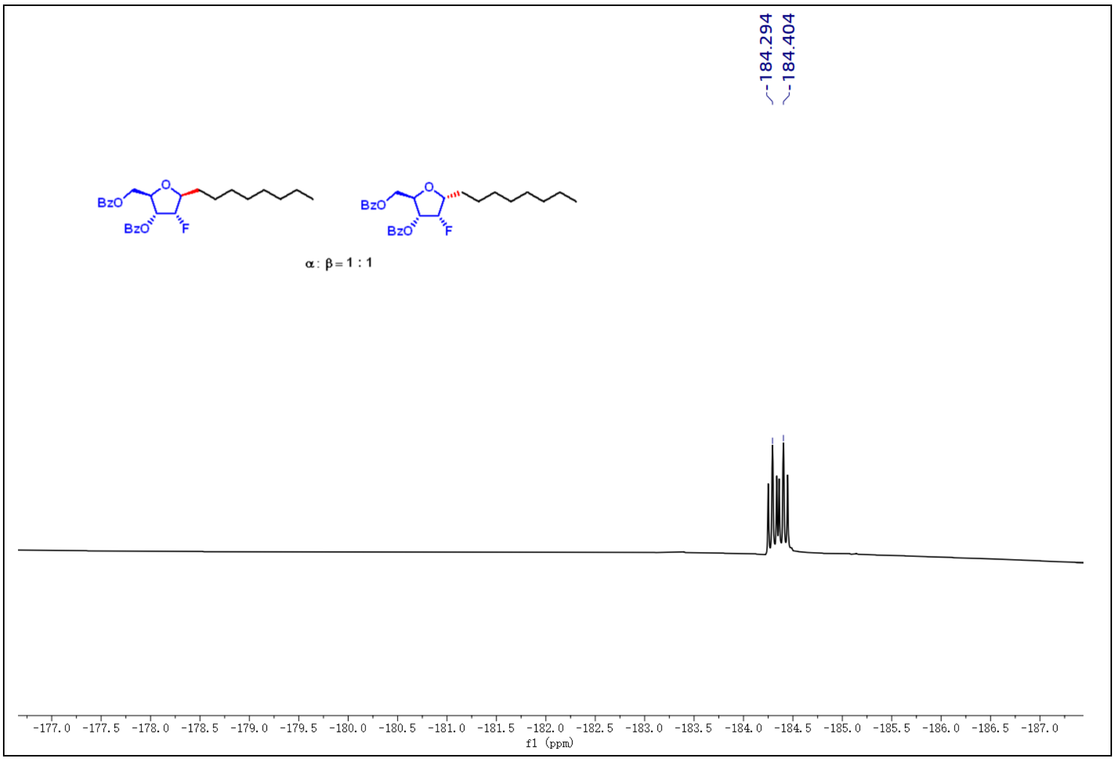
-184.294
-184.404
BzO
BzO
F
BzO
BzO
F
α : β = 1 : 1
-177.0 -177.5 -178.0 -178.5 -179.0 -179.5 -180.0 -180.5 -181.0 -181.5 -182.0 -182.5 -183.0 -183.5 -184.0 -184.5 -185.0 -185.5 -186.0 -186.5 -187.0
f1 (ppm)

(2*R*,3*R*,4*R*,5*R*,6*R*)-2-octyl-6-((((2*R*,3*R*,4*S*,5*R*,6*R*)-3,4,5-triacetoxy-6-(acetoxymethyl)tetrahydro-2*H*-pyran-2-yl)oxy)methyl)tetrahydro-2*H*-pyran-3,4,5-triyl tribenzoate (3ja)

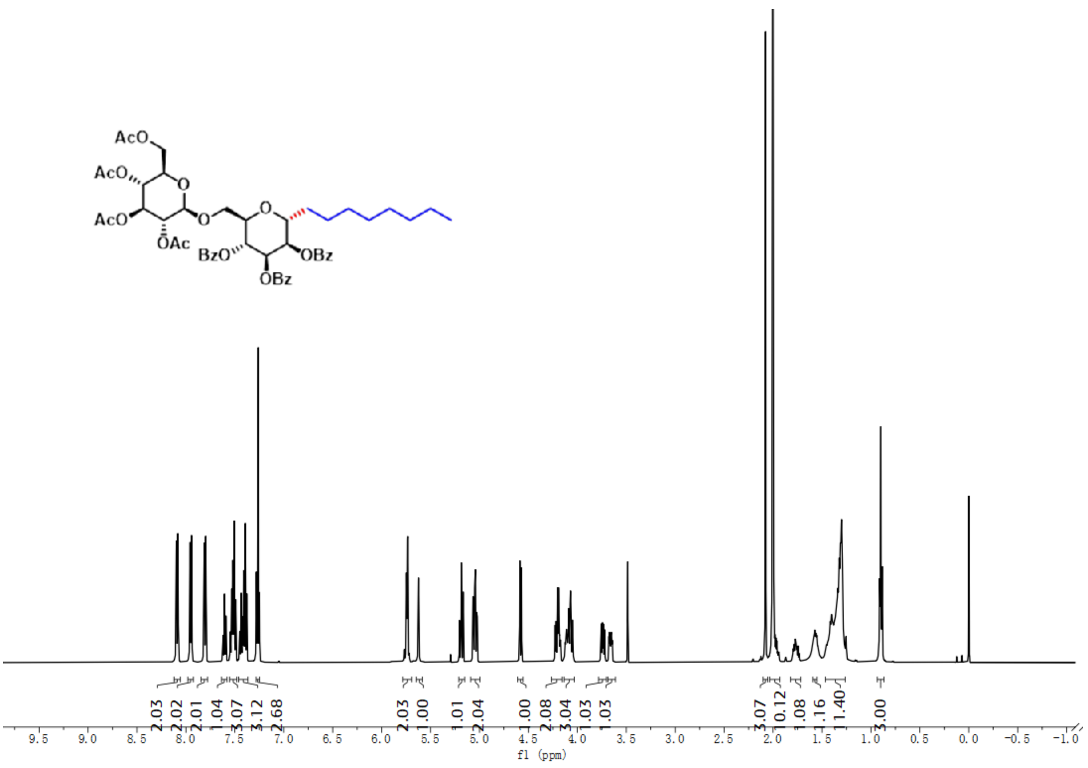

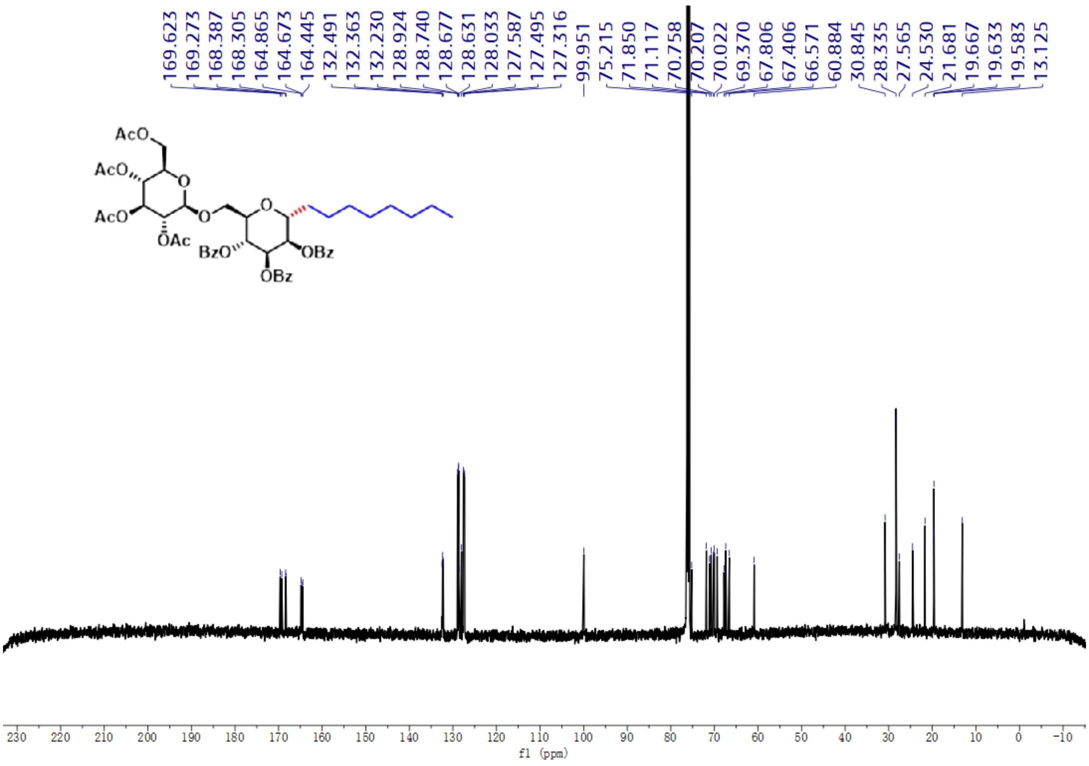

(2*R*,3*R*,4*R*,5*R*,6*R*)-2-octyl-6-((((2*R*,3*R*,4*S*,5*S*,6*R*)-3,4,5-triacetoxy-6-(acetoxymethyl)tetrahydro-2*H*-pyran-2-yl)oxy)methyl)tetrahydro-2*H*-pyran-3,4,5-triyl tribenzoate (3ka)

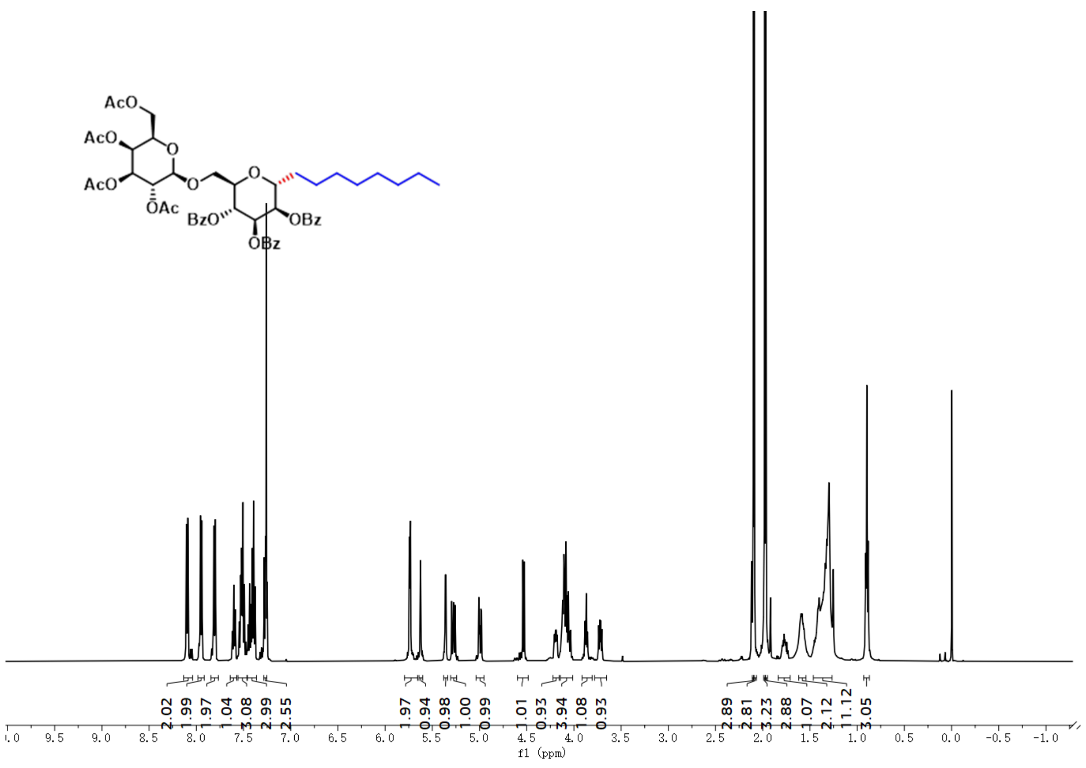

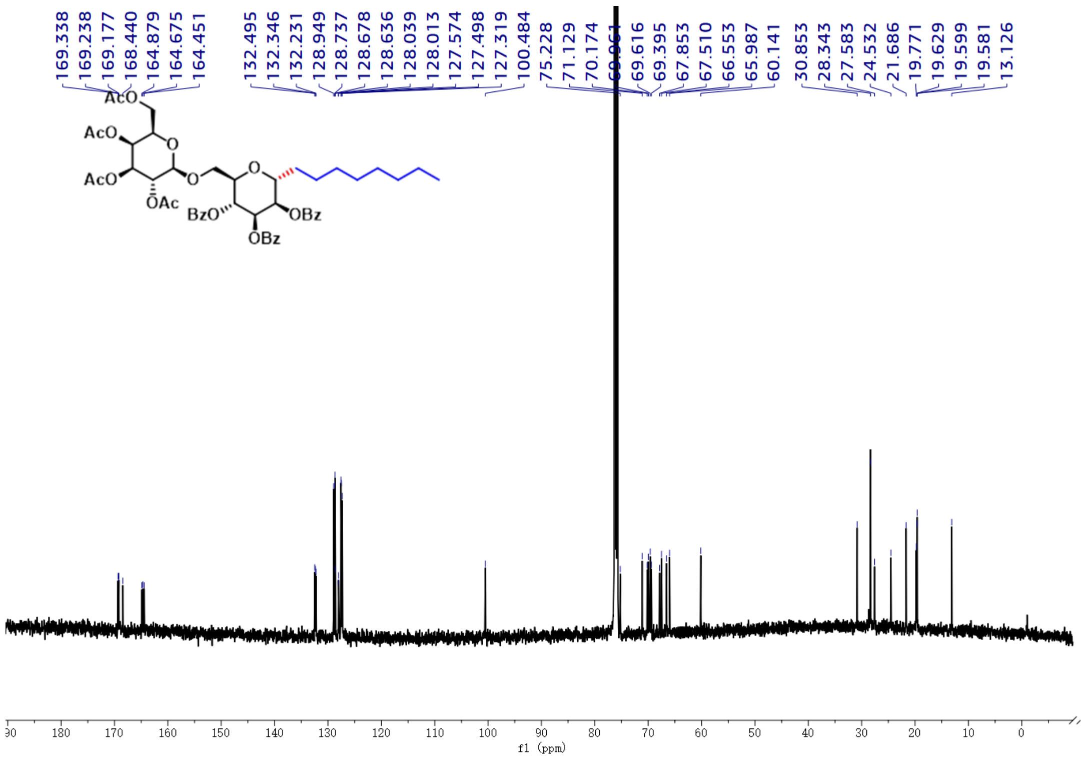

N-(4-((5-((2*R*,3*S*,4*R*,5*S*,6*R*)-3,4,5-trihydroxy-6-(hydroxymethyl)tetrahydro-2*H*-pyran-2-yl)pentyl)oxy)phenyl)acetamide (4an)

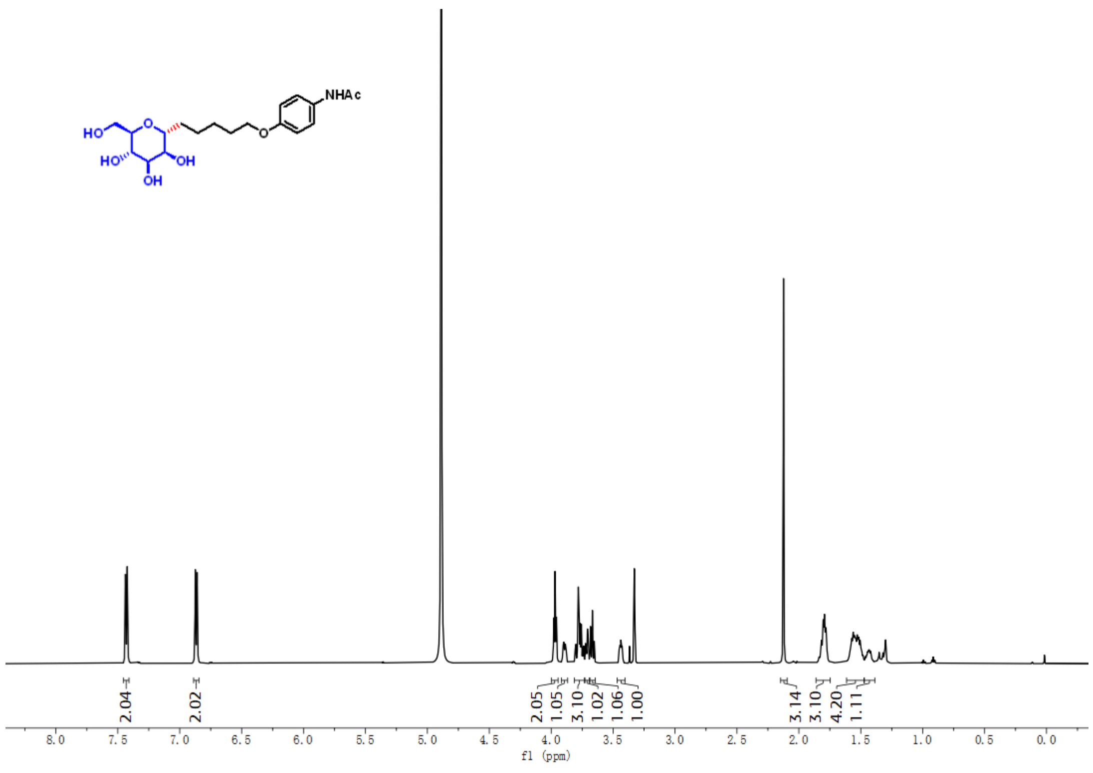

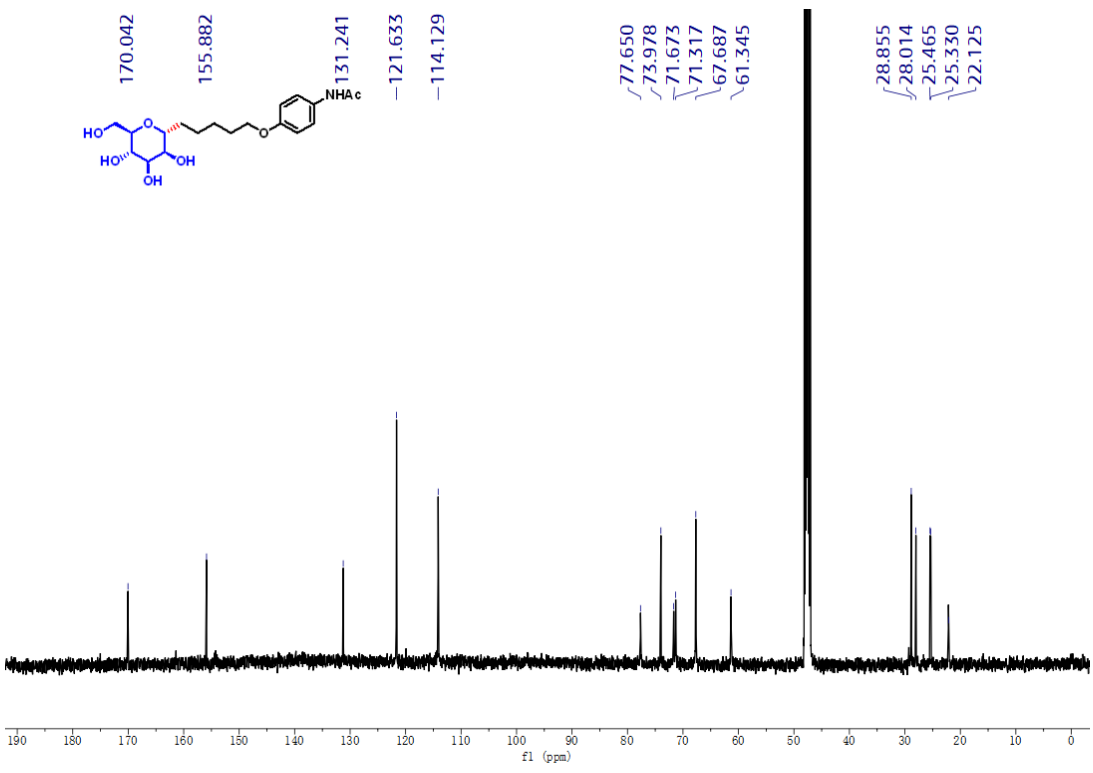

(2*R*,3*R*,4*R*,5*R*,6*R*)-2-((benzoyloxy)methyl)-6-(5-(((4a*S*,6a*S*,6b*R*,10*S*,12a*R*)-10-hydroxy-2,2,6a,6b,9,9,12a-heptamethyl-1,2,3,4,4a,5,6,6a,6b,7,8,8a,9,10,11,12,12a,12b,13,14b-icosahydropicene-4a-carbonyl)oxy)pentyl)tetrahydro-2*H*-pyran-3,4,5-triyl tribenzoate

(5aa)

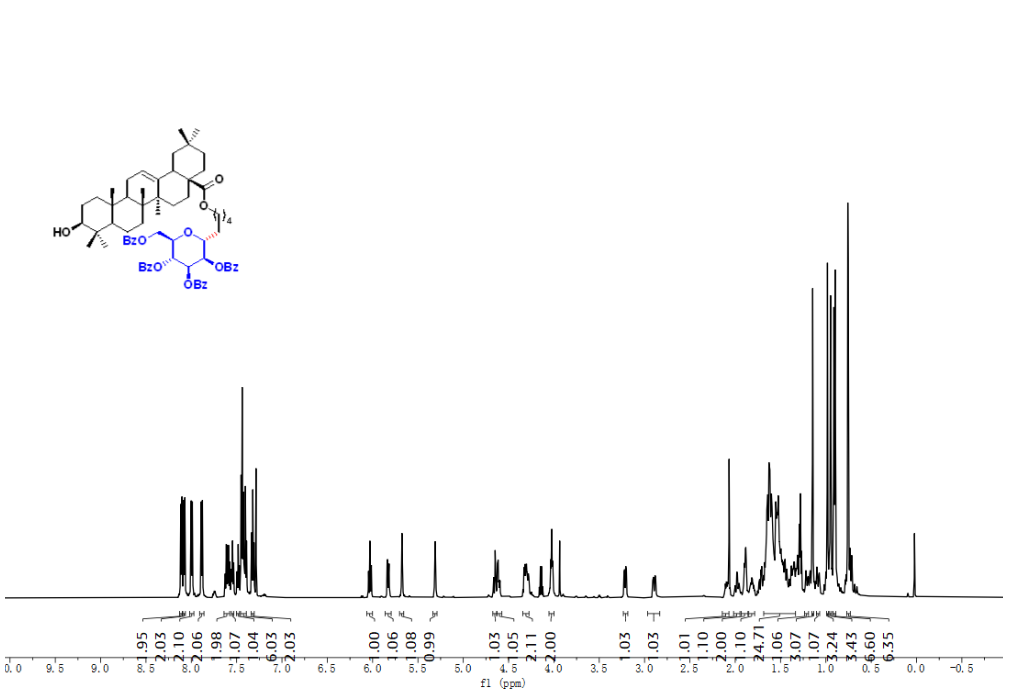

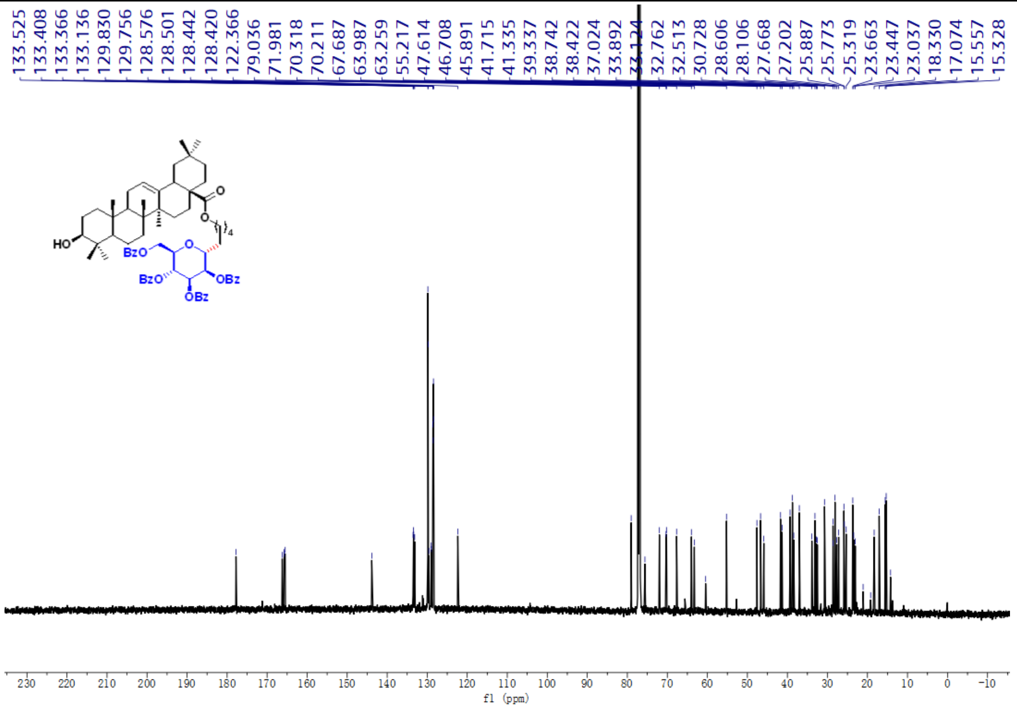

(2*R*,3*R*,4*R*,5*R*,6*R*)-2-((benzoyloxy)methyl)-6-((*R*)-4-((tert-

butoxycarbonyl)amino)-5-(((*S*)-1-methoxy-1-oxopropan-2-yl)amino)-5-oxopentyl)tetrahydro-2*H*-pyran-3,4,5-triyl tribenzoate (6aa)

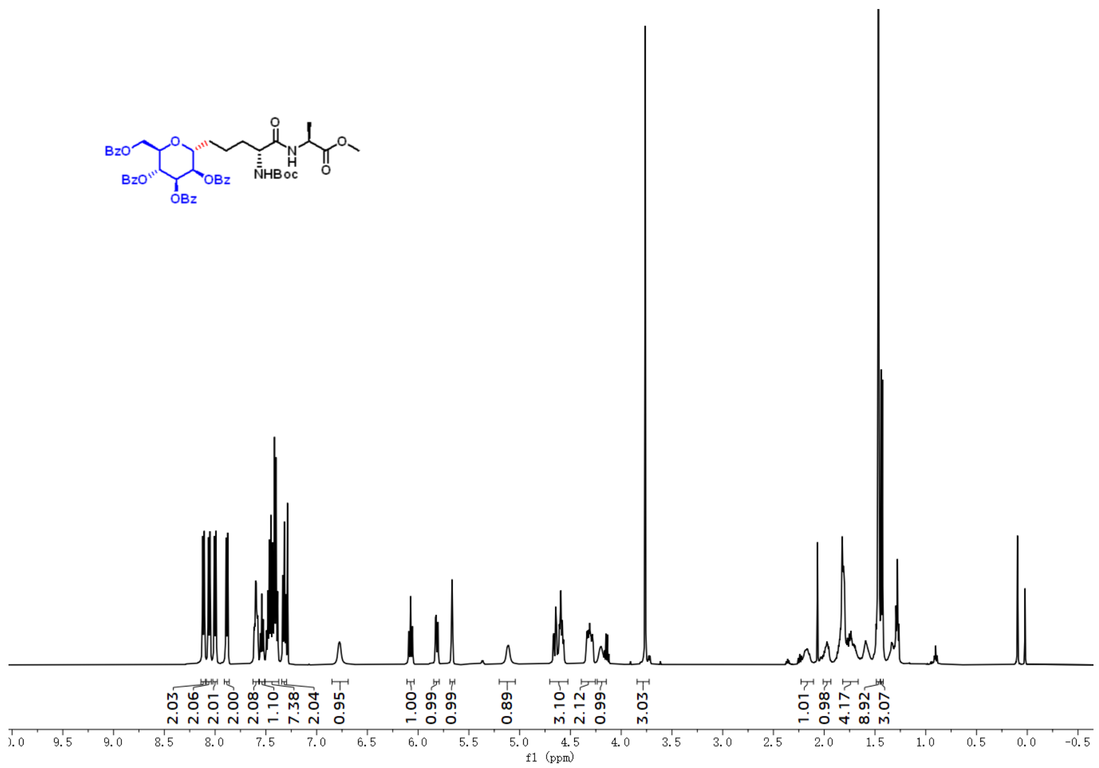

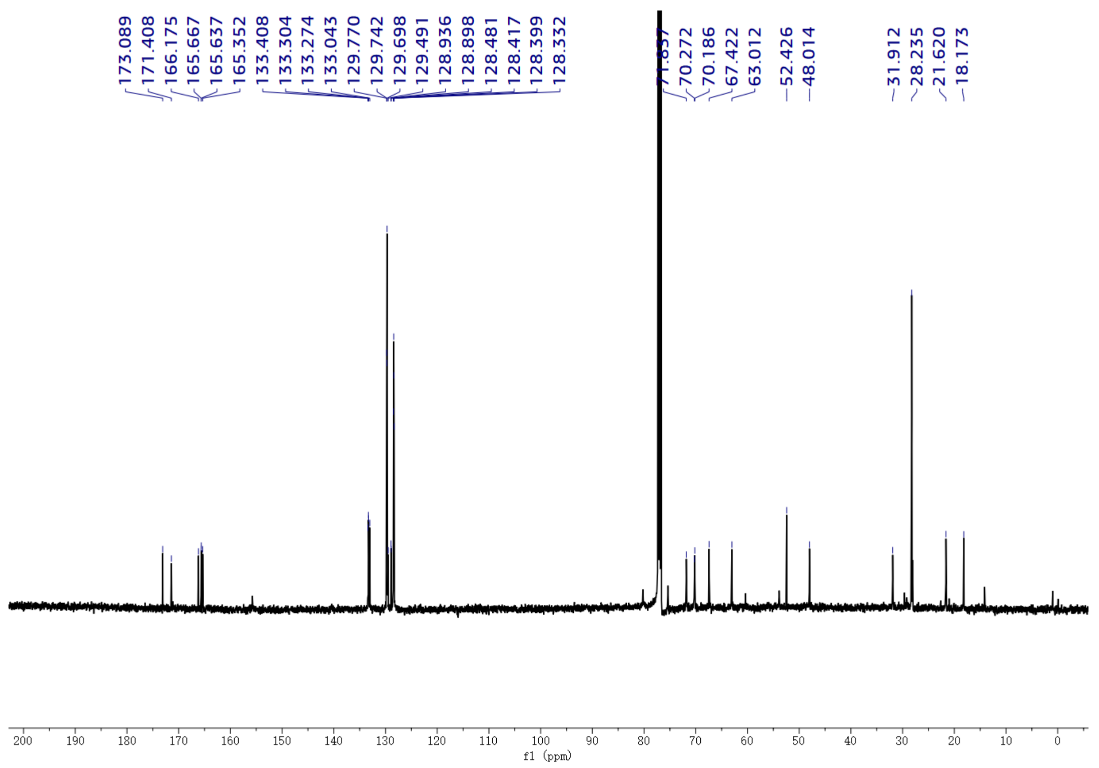

(2*R*,3*R*,4*R*,5*R*,6*R*)-2-((benzoyloxy)methyl)-6-((*R*)-4-((tert-butoxycarbonyl)amino)-5-((2-((2-methoxy-2-oxoethyl)amino)-2-oxoethyl)amino)-5-oxopentyl)tetrahydro-2*H*-pyran-3,4,5-triyl tribenzoate (7aa)

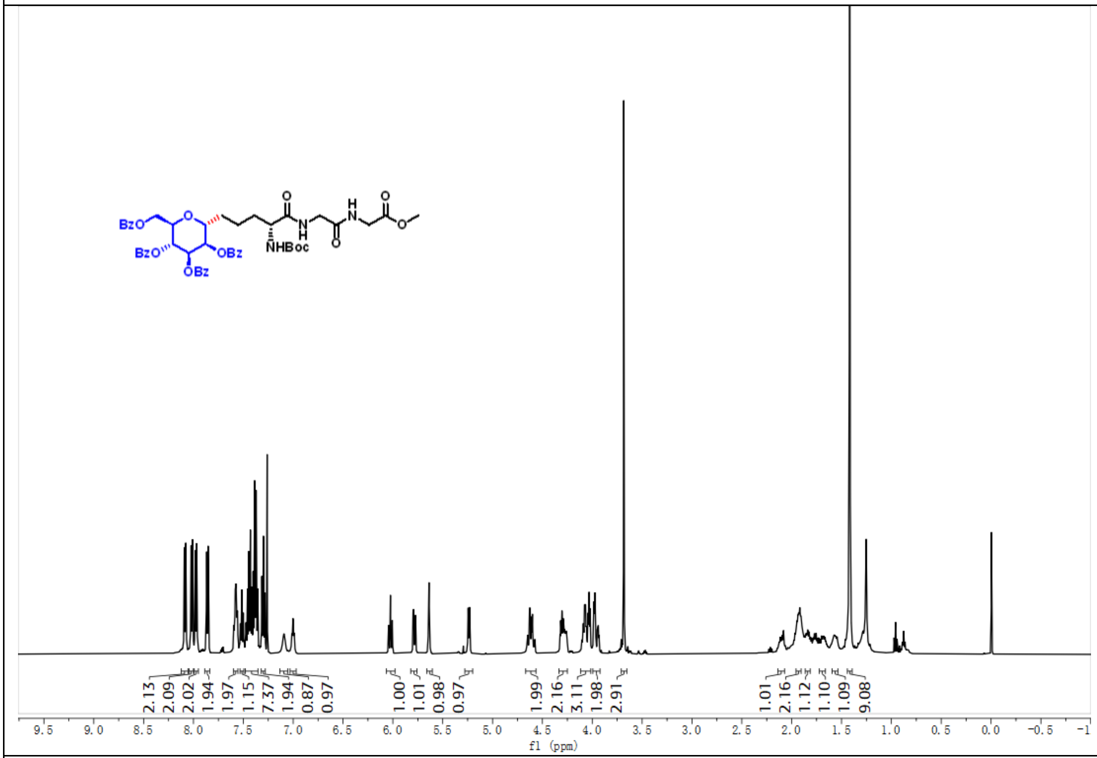

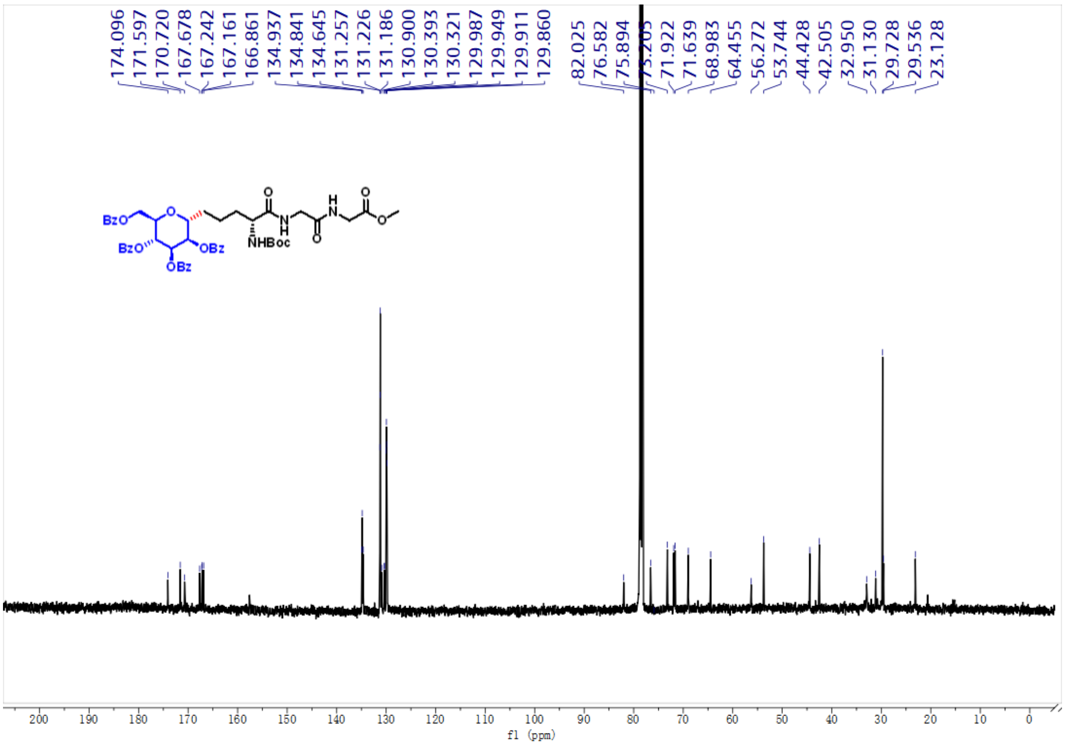

2-((benzoyloxy)methyl)-6-(octyl-2-d)tetrahydro-2*H*-pyran-3,4,5-triyl tribenzoate (d-3aa)

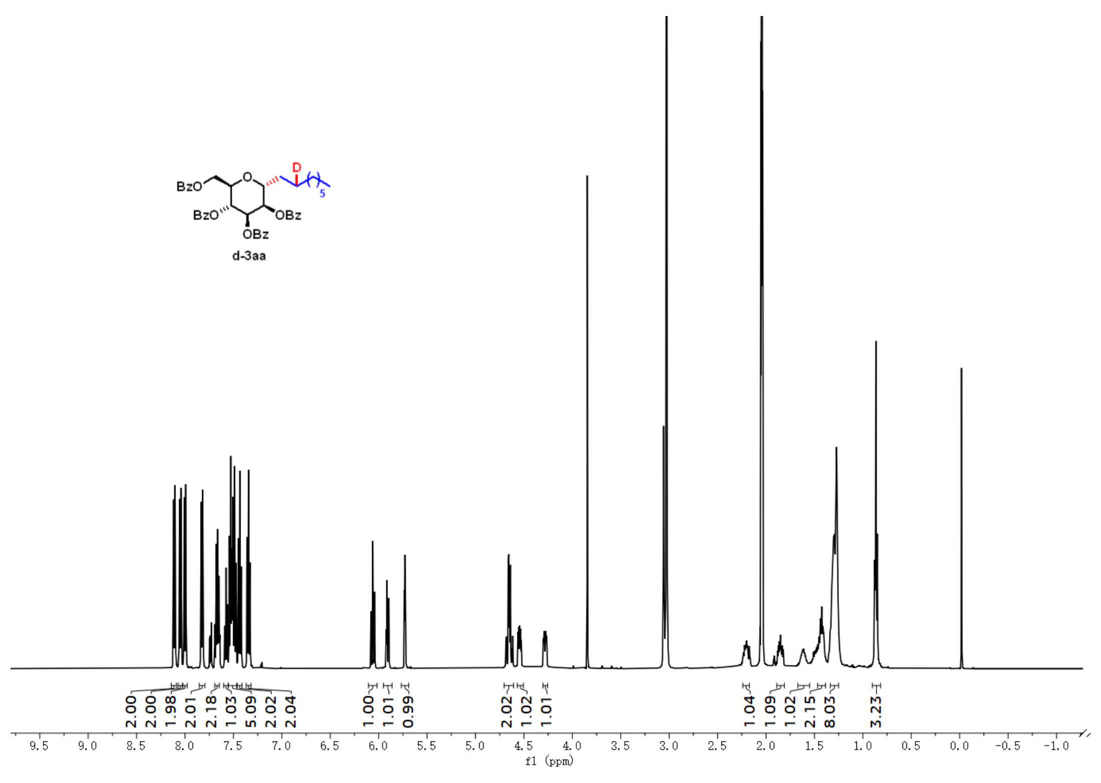

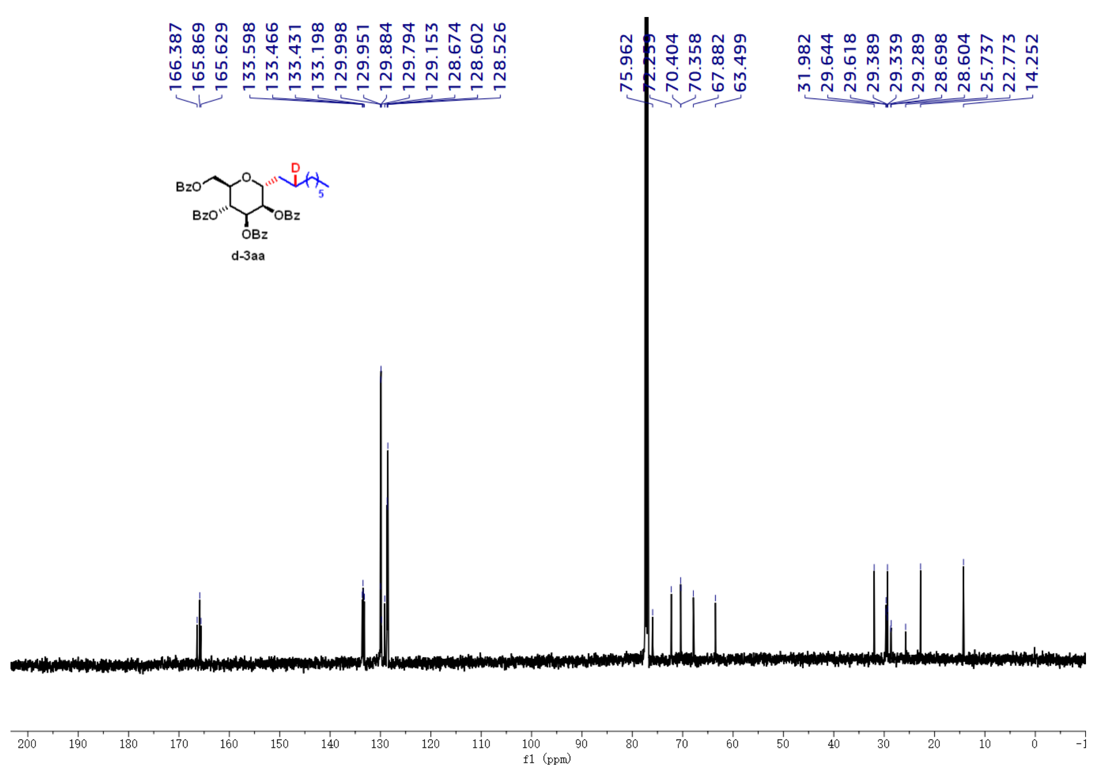

Supplement: Supplementary file 1 — Supporting Information [file ADVS-11-2307226-s001.pdf]
